# Action of m6A-related gene signatures on the prognosis and immune microenvironment of colonic adenocarcinoma

**DOI:** 10.1016/j.heliyon.2024.e31441

**Published:** 2024-05-24

**Authors:** Han Shugao, Wu Yinhang, Zhuang Jing, Qu Zhanbo, Da Miao

**Affiliations:** a Department of Radiology, The Second Affiliated Hospital of Zhejiang University School of Medicine, Hangzhou, China; b Huzhou Central Hospital, Affiliated Central Hospital Huzhou University, Huzhou, China; c Huzhou Central Hospital, Fifth School of Clinical Medicine of Zhejiang Chinese Medical University, Huzhou, China; d Key Laboratory of Multiomics Research and Clinical Transformation of Digestive Cancer of Huzhou, Huzhou, China; e Huzhou Third Municipal Hospital, the Affiliated Hospital of Huzhou University, Huzhou, China

**Keywords:** Colonic adenocarcinoma, N6-methyladenosine, Prognostic model, Immune microenvironment, Gene signature

## Abstract

N6-methyladenosine (m6A) modification in human tumor cells exerts considerable influence on crucial processes like tumorigenesis, invasion, metastasis, and immune response. This study aims to comprehensively analyze the impact of m6A-related genes on the prognosis and immune microenvironment (IME) of colonic adenocarcinoma (COAD). Public data sources, predictive algorithms identified m6A-related genes and differential gene expression in COAD. Subtype analysis and assessment of immune cell infiltration patterns were performed using consensus clustering and the CIBERSORT algorithm. The Least Absolute Shrinkage and Selection Operator (LASSO) regression analysis determined gene signatures. Independent prognostic factors were identified using univariate and multivariate Cox proportional hazards models. The findings indicate that 206 prognostic m6A-related DEGs contribute to the m6A regulatory network along with 8 m6A enzymes. Based on the expression levels of these genes, 438 COAD samples from The Cancer Genome Atlas (TCGA) were classified into 3 distinct subtypes, showing marked differences in survival prognosis, clinical characteristics, and immune cell infiltration profiles. Subtype 3 and 2 displayed reduced levels of infiltrating regulatory T cells and M0 macrophages, respectively. A six-gene signature, encompassing KLC3, SLC6A15, AQP7 JMJD7, HOXC6, and CLDN9, was identified and incorporated into a prognostic model. Validation across TCGA and GSE39582 datasets exhibited robust predictive specificity and sensitivity in determining the survival status of COAD patients. Additionally, independent prognostic factors were recognized, and a nomogram model was developed as a prognostic predictor for COAD. In conclusion, the six target genes governed by m6A mechanisms offer substantial potential in predicting COAD outcomes and provide insights into the unique IME profiles associated with various COAD subtypes.

## 1 Background

Colorectal cancer (CRC) stands as a prominent global gastrointestinal malignancy characterized by its substantial cancer incidence and mortality rates [1]. The global estimate suggests a yearly increase of 4.2 % in CRC incidence, with over 1.4 million new cases identified in 2020 alone [2]. Among the diverse pathological forms of CRC, colonic adenocarcinoma (COAD) predominates, often arising from dysregulated hyperplasia in the colonic mucosal epithelium that evolves into malignancy through invasive progression [3,4]. Enhanced insights into the pathophysiology of COAD have broadened therapeutic avenues, contributing to a twofold increase in the overall survival rate of advanced-stage patients [5]. Despite notable advancements in therapeutic approaches encompassing surgical interventions, chemotherapy, and targeted treatments, COAD patients remain susceptible to a 50 % likelihood of distal metastasis, and the five-year survival rate plummets to 8.1 % post-metastasis [6]. Consequently, augmenting early-stage detection, timely intervention, and the ability to predict prognostic risk holds profound implications for enhancing clinical outcomes among COAD patients.

Epigenetic aberrations within the normal colonic epithelium may precipitate the development of colorectal adenomas and invasive adenocarcinomas, wherein methylation emerges as a pivotal epigenetic regulator in COAD [7,8]. Notably, the perturbation of gene expression via epigenetic mechanisms incites changes in the phenotype of tumor-associated stromal cells, integral players within the tumor microenvironment [9]. Moreover, hypermethylation of several tumor suppressor genes, such as INK4a, is also associated with a poor prognosis of COAD [10]. N6-methyladenosine (m6A), a form of methylation modification, has recently captivated extensive research attention. It occurs at the N6 site of adenosine and is recognized as the most abundant and conserved internal transcriptional modification of messenger RNA in eukaryotic cells [11,12]. m6A is catalyzed by m6A methyltransferases (writers), removed by m6A demethylases (erasers), and recognized by binding proteins (readers) [13]. m6A methylation affects various aspects of RNA regulation, including RNA expression, splicing, nuclear output, translation, and RNA-protein interactions [14]. It is also believed that m6A modification in tumor cells has significant effects on tumor genesis, proliferation, invasion, metastasis, and immune response by regulating a variety of oncogenes and tumor suppressor genes [15]. As a promising biomarker, m6A has been increasingly used for the detection and prevention of cancer. The YT521-B homology (YTH) domain family of m6A readers, encompassing YTHDF1, YTHDF3, and YTHDC2, may contribute to the detection, progression, and prognosis of COAD [16]. Besides, investigations spotlight ALKBH5 and YTHDF1 as agents influencing immune contexture within COAD patients [17]. However, a comprehensive understanding of how m6A modifications orchestrate COAD prognosis and shape the immune microenvironment remains partially illuminated.

**Fig. 1:**
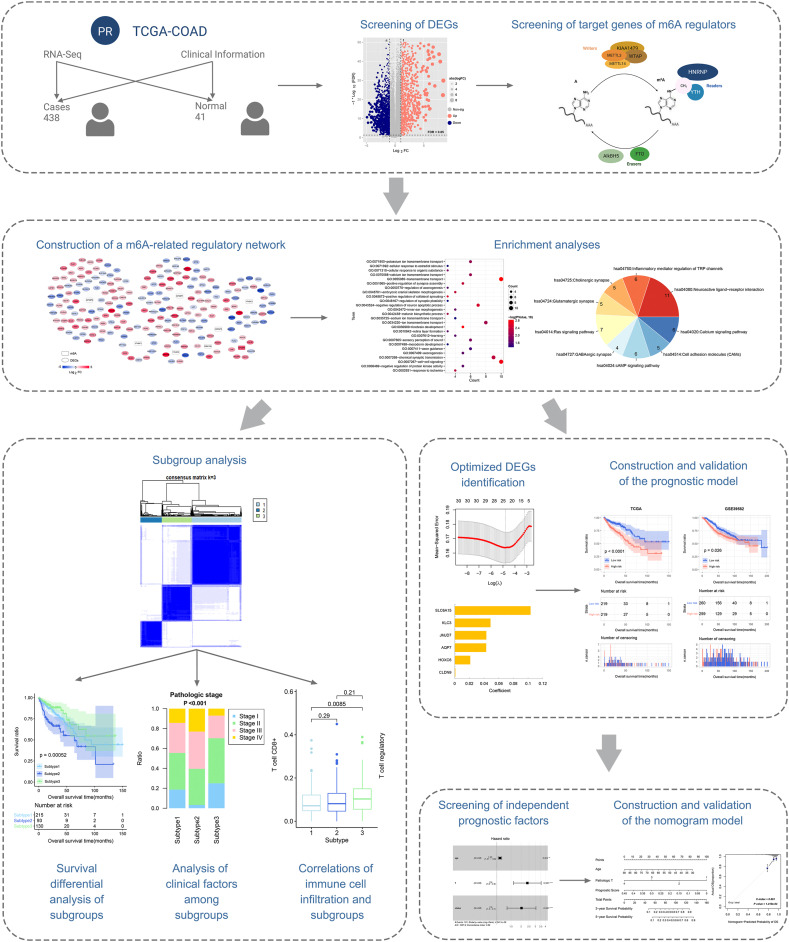
The flow layout of this study.

Presently, the realm of bioinformatics has yielded methodological advancements aimed at scrutinizing potential prognostic biomarkers and therapeutic targets for malignancies. Liu et al., for instance, unearthed m6A RNA methylation regulators (YTHDF1 and HNRNPC) as indicative of the prognostic risk inherent in COAD [18]. In this inquiry, we navigate the prognostic landscape of m6A-related genes, harnessing the power of bioinformatics methodologies. However, our endeavor distinguishes itself through its focus on the target genes governed by m6A methylation regulators, integrating them as the subjects of our investigation. These gene candidates were subsequently culled by intersecting with the pool of differentially expressed genes (DEGs) associated with COAD. It merits highlighting that our exploration extends beyond mere gene identification. We ventured to erect a prognostic model, coupled with a histogram prediction model, both designed to substantiate the prognostic utility embedded within these gene signatures. Concurrently, we embarked on a comprehensive analysis of the intricate interplay between m6A-related prognostic genes, the tumor immune microenvironment, and distinct disease subtypes. This collective effort serves to amplify our comprehension of the m6A regulatory network, a central focal point of our inquiry. The encapsulation of our research workflow is depicted schematically in Fig. 1. Of particular note, the mined m6A-related gene signatures, gleaned from public repositories, can be harnessed as a pioneering biomarker. Their potential deployment lies in the realm of prognostic risk prediction for COAD, thereby ushering in avenues to augment the clinical outcomes for individuals grappling with this affliction.

## 2 Methods

### 2.1 Data sources

The RNA-seq data and clinical information for 438 COAD samples and 41 control samples were extracted from TCGA database, forming the foundation of our training dataset. Meanwhile, using thekeywords “colon adenocarcinoma” and “*Homo sapiens*”, microarrays were obtained from the gene expression omnibus (GEO) database [19] with the following criteria: 1) Incorporation of tumor tissue samples of COAD patients; 2) A COPD sample size exceeding 200; 3) Incorporation of COAD samples endowed with clinical prognostic particulars. According to the above standards, only the GSE39582 dataset [20] was selected as the training set. The GSE39582 dataset was detected using the GPL570 Affymetrix Human Genome U133 Plus 2.0 Array, and included 585 samples in total, of which 519 COAD samples had survival prognostic information.

### 2.2 Screening of DEGs

DEGs from the COAD samples and controls were screened using the limma package (version 3.34.4) [21] with marginal values of false discovery rate (FDR) < 0.05, and |log_2_fold change (FC)| > 1 for inclusion. Subsequently, based on the centered Pearson correlation algorithm [22], a two-way hierarchical clustering was applied to the expression values of the identified DEGs. This operation was carried out using the R3.6.1 pheatmap package (version 1.0.8) [23], ensuring an effective visualization of the clustering patterns.

### 2.3 Regulatory network construction of m6A-related genes

To obtain downstream genes regulated by m6A, m6A2Target (http://m6a2target.canceromics.org/) [24] was used to predict m6A-related mRNAs. By comparing DEGs with m6A-related genes, the intersecting genes were retained as m6A-related DEGs. Subsequently, the univariate Cox regression analysis from the R-3.6.1 survival package (version 2.41.1) [25] was used to screen m6A-related DEGs that demonstrated significant correlation with survival prognosis. The relationships between m6A enzymes and m6A-related prognostic DEGs were constructed into a network and visualized using Cytoscape3.6.1 (https://cytoscape.org/) [26]. To further decipher the biological processes and pathways potentially implicated by the DEGs within the network, enrichment analysis was performed through DAVID 6.8 [27,28], with a significance threshold of P < 0.05 employed for the screening of meaningful correlations.

### 2.4 Subgroup analysis

Utilizing the expression profiles of m6A-associated prognostic DEGs derived from the aforementioned regulatory network within COAD tumor samples, we leveraged ConsensusClusterPlus (version 1.54.0) [29] for conducting a comprehensive analysis to delineate tumor subtypes across all samples. Employing the R3.6.1 survival package, we crafted Kaplan–Meier (KM) survival curves to discern variations in survival prognosis among distinct subtype samples. Following this, a thorough statistical comparison of clinical attributes within divergent subtype samples ensued. Notably, a significance threshold of P < 0.05 was judiciously employed to identify pertinent correlations.

### 2.5 Correlation analysis of subgroup and immune infiltration

The deconvolution algorithm CIBERSORT [30] was employed to meticulously compute the proportions of distinct immune cell types within each individual sample. Subsequently, utilizing the R3.6.1 aov function, we performed a robust analysis to ascertain disparities in immune cell proportions across diverse subtypes.

### 2.6 Construction of a prognostic model

Building upon the m6A-related prognostic DEGs within the regulatory network, we conducted multivariate Cox analysis to meticulously identify independent prognostic m6A-related DEGs, utilizing the R3.6.1 survival package. A significance level of Log-rank P < 0.05 was deemed as statistically meaningful. Subsequently, we performed a robust least absolute shrinkage and selection operator (LASSO) regression analysis on the pool of independent prognostic m6A-related DEGs. This analysis aimed to pinpoint an optimal subset of gene signatures. Following this, we calculated the prognostic score (PS) for each sample employing the subsequent formula:

PS = ∑Coef_genes_ × Exp_genes_

Within this equation, "Coef_genes_" represents the LASSO regression coefficient associated with the gene signatures, while "Exp_genes_" embodies the expression levels of these gene signatures within the training set. To gauge the connection between the expression of gene signatures and the observed survival prognosis of COAD samples, we conducted Kaplan-Meier (KM) analysis. The calculated prognostic score (PS) was then applied to both the TCGA training set and the GSE39582 dataset, categorizing samples into high-risk and low-risk groups, aligned with their respective median PS values. We also investigated the disparity in survival prognosis between these risk-defined groups, and the findings were depicted through Kaplan-Meier (KM) curves.

### 2.7 Principal component analysis (PCA)

To observe the difference in m6A status among patients at different risk, PCA analysis was further generated using the R3.6.1 psych package (version1.7.8) [31,32] based on the expression levels of m6A-related genes in COAD samples from TCGA dataset.

### 2.8 Construction of a nomogram prediction model

To assess the self-reliant prognostic capability of the prognostic score (PS) in tandem with clinical attributes, univariate and multivariate Cox proportional hazards regression models were established utilizing the R3.6.1 survival package, with statistical significance determined at log-rank P < 0.05. Building on the identified independent prognostic factors, we developed a nomogram model to prognosticate survival probabilities, employing the R3.6.1 rms package (version 5.1-2) [33,34]. Subsequently, the R3.6.1 survcomp (version 1.34.0) was applied to compute the C-index, thereby appraising the model's predictive capability [[35], [36], [37]].

### 2.9 Statistical analysis

SPSS V22.0 (SPSS Inc., Chicago, IL) was selected to conduct statistical analysis. Independent sample *t*-test was used for continuous variables, and Pearson's chi-square test was used for inter-group categorical variables according to hypothesized validity.

## 3 Results

### 3.1 Screening of DEGs between COAD and control groups

**Fig. 2:**
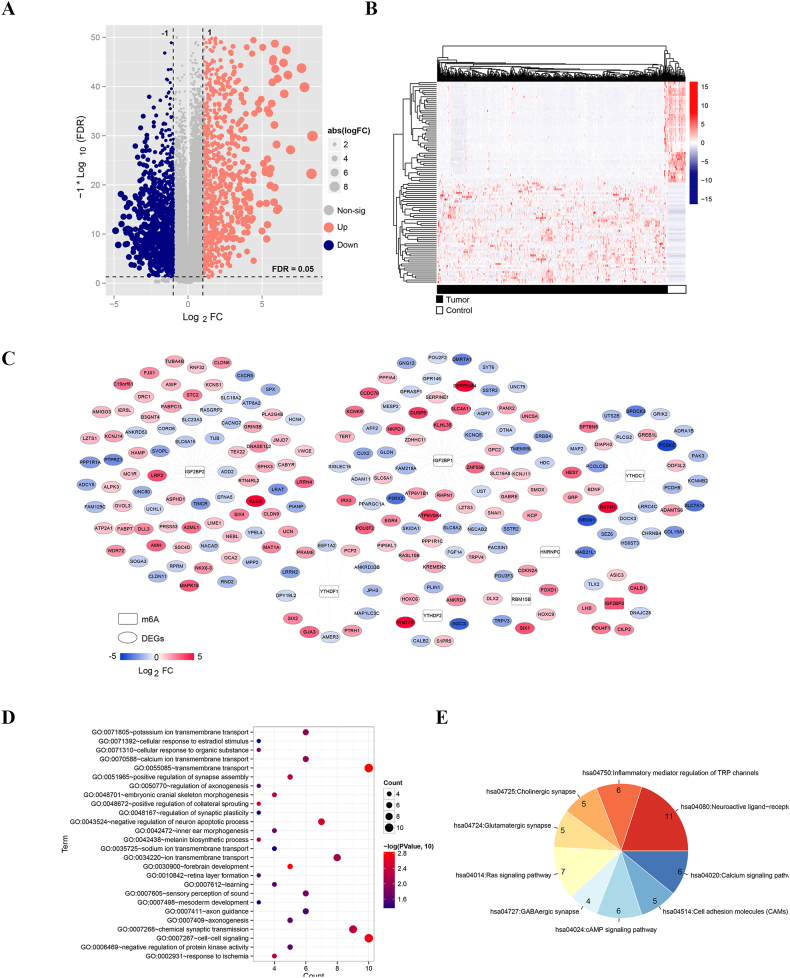
Screening of prognostic m6A-related DEGs and enrichment analyses. A: The volcano plot illustrates the differential expression of upregulated and downregulated DEGs between the two groups, determined by a cutoff of FDR <0.05 and |log2FC| > 1. B: The heatmap showcases the expression profiles of the top 50 upregulated and downregulated DEGs, ranked according to Log2FC values. C: The m6A regulatory network encompasses eight m6A enzymes and 206 prognostic m6A-related DEGs. M6A enzymes are represented by squares, while prognostic m6A-related DEGs are depicted by ovals. D: The bubble diagram visually presents the 26 enriched biological processes associated with prognostic m6A-related DEGs. The x-axis corresponds to gene count, and the y-axis denotes the range of biological process terms. E: The pie chart provides insight into the distribution of the nine enriched KEGG pathways linked to prognostic m6A-related DEGs.

By analyzing the expression data of 438 tumor samples and 41 control samples, 2422 DEGs satisfying the set threshold were screened, of which 1120 DEGs (including DAO, SLC17A8, VSTM2A, etc.) were upregulated in COAD group and 1302 DEGs (C8orf74, IGFL1, ZIC5, etc.) were downregulated in COAD group, as shown in the volcano plot in Fig. 2A and Table S1. Afterwards, the top 50 upregulated and downregulated DEGs ranked by Log_2_FC were selected for bidirectional hierarchical clustering analysis, and the heatmap in Fig. 2B showed that the expression of these DEGs was significantly different between the two groups.

### 3.2 Analyses of prognostic m6A-related DEGs

To compare the DEGs and the m6A-related mRNAs predicted in the public databases, 1269 m6A-related DEGs were obtained from public databases. Totally, 206 prognostic m6A-related DEGs were screened out by applying univariate Cox regression analysis. The regulatory network of prognostic m6A-related DEGs and m6A enzymes was constructed and is shown in Fig. 2C. This network contains 8 m6A enzymes and 206 prognostic m6A-related DEGs. Subsequently, enrichment analyses were performed on these prognostic m6A-related DEGs, and 26 biological processes and nine Kyoto Encyclopedia of Genes and Genomes (KEGG) pathways were obtained (Table 1). The results showed that the feature genes were most significantly associated with BP of "transmembrane transport" (GO:0055085), "cell-cell signaling" (GO:0007267), and "chemical synaptic transmission" (GO:0007268) (Fig. 2D). Notably, these genes were mainly enriched in the following 9 KEGG pathways such as "neuroactive ligand-receptor interaction", "Ras signaling pathway" etc. (Fig. 2E)

**Table 1:** Biological processes and KEGG pathways enriched on 206 prognostic m6A-related DEGs.

Category	Term	Count	*P*-value
Biology Process	GO:0055085∼transmembrane transport	10	1.40E-03
	GO:0030900∼forebrain development	5	1.53E-03
	GO:0007267∼cell-cell signaling	10	1.83E-03
	GO:0048672∼positive regulation of collateral sprouting	3	3.13E-03
	GO:0043524∼negative regulation of neuron apoptotic process	7	3.17E-03
	GO:0048701∼embryonic cranial skeleton morphogenesis	4	4.50E-03
	GO:0051965∼positive regulation of synapse assembly	5	4.57E-03
	GO:0007268∼chemical synaptic transmission	9	4.72E-03
	GO:0002931∼response to ischemia	4	5.38E-03
	GO:0034220∼ion transmembrane transport	8	8.04E-03
	GO:0042438∼melanin biosynthetic process	3	8.42E-03
	GO:0070588∼calcium ion transmembrane transport	6	9.58E-03
	GO:0071805∼potassium ion transmembrane transport	6	1.02E-02
	GO:0071310∼cellular response to organic substance	3	1.43E-02
	GO:0007605∼sensory perception of sound	6	1.49E-02
	GO:0042472∼inner ear morphogenesis	4	1.88E-02
	GO:0007409∼axonogenesis	5	2.19E-02
	GO:0006469∼negative regulation of protein kinase activity	5	2.27E-02
	GO:0010842∼retina layer formation	3	2.34E-02
	GO:0007612∼learning	4	2.39E-02
	GO:0050770∼regulation of axonogenesis	3	2.55E-02
	GO:0007411∼axon guidance	6	2.97E-02
	GO:0071392∼cellular response to estradiol stimulus	3	4.42E-02
	GO:0035725∼sodium ion transmembrane transport	4	4.49E-02
	GO:0007498∼mesoderm development	3	4.69E-02
	GO:0048167∼regulation of synaptic plasticity	3	4.69E-02
KEGG Pathway	hsa04080:Neuroactive ligand-receptor interaction	11	9.59E-04
	hsa04750:Inflammatory mediator regulation of TRP channels	6	4.66E-03
	hsa04725:Cholinergic synapse	5	3.54E-02
	hsa04724:Glutamatergic synapse	5	3.84E-02
	hsa04014:Ras signaling pathway	7	4.03E-02
	hsa04727:GABAergic synapse	4	4.69E-02
	hsa04024:cAMP signaling pathway	6	4.70E-02
	hsa04514:Cell adhesion molecules (CAMs)	5	4.74E-02
	hsa04020:Calcium signaling pathway	6	4.96E-02

KEGG: Kyoto Encyclopedia of Genes and Genomes; DEG: differential expressed gene.

*P* value < 0.05 indicates statistical significances.

### 3.3 Analysis of COAD subtypes

**Fig. 3:**
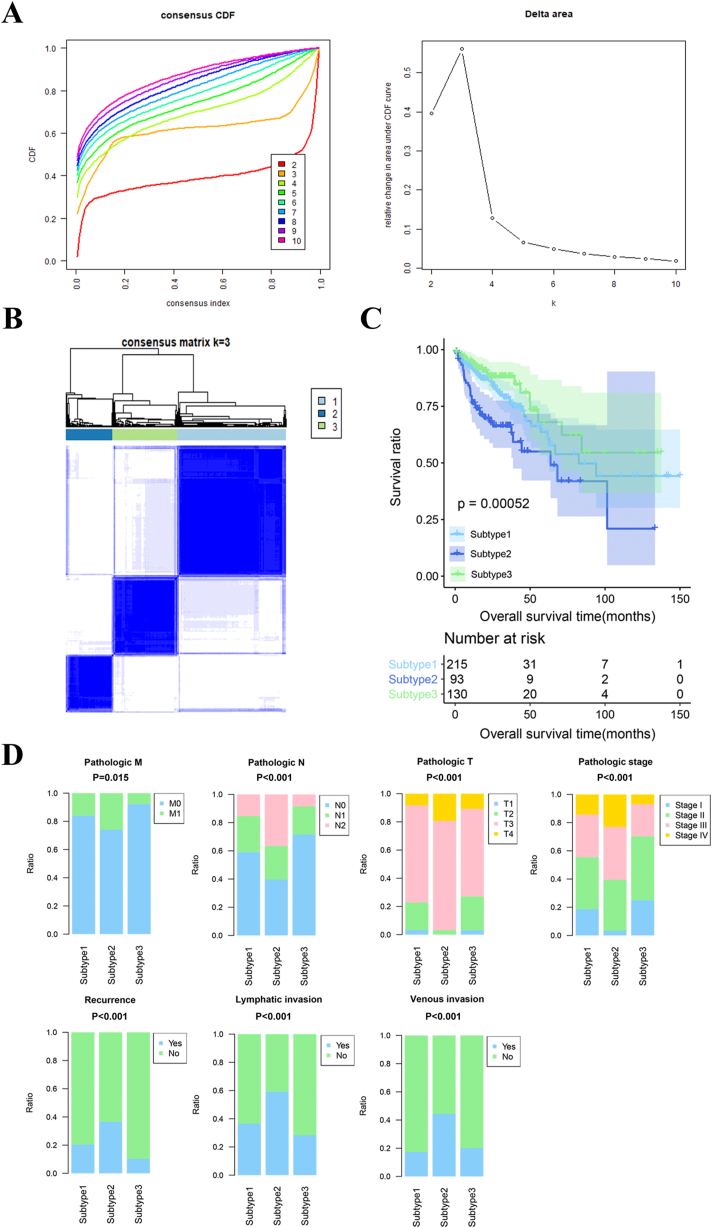
Subtype analysis of COAD samples. A: The empirical cumulative distribution function (CDF) plot visually depicts the consensus distributions for each value of k. The x-axis denotes the consensus index, while the y-axis represents the cumulative distribution function. The y-axis of the delta area plot indicates the proportional increment in cluster stability. B: The consensus heatmap vividly portrays three distinct clusters corresponding to COAD subtypes, encompassing 215, 93, and 130 COAD samples, respectively. C: The Kaplan-Meier (KM) curves provide a clear visualization of the survival disparities observed among the three identified subtypes. D: The graphical representation illustrates the distribution of patients with varying clinical attributes across the three subtypes.

Based on the expression levels of prognostic m6A-related DEGs, subtype analysis was conducted on the COAD samples within the TCGA training set via consensus clustering. The point of maximum stability, indicative of optimal k-means cluster selection, was observed at k = 3, as depicted by the convergence of the distribution (Fig. 3A). Illustrated by the consensus heatmap in Fig. 3B, the COAD subtypes were stratified into three distinct clusters, encompassing 215, 93, and 130 samples, respectively. Survival analysis (Fig. 3C) suggested that the survival status of the 3three subtypes (P = 0.00052). Specifically, subtype 3 displayed the most favorable survival ratio, whereas subtype 2 exhibited the least favorable. The clinical attributes of samples originating from diverse subtypes were subjected to statistical comparison, as outlined in Table 2. Evidently, significant differences emerged among the three subtypes in terms of pathological TNM, pathological stage, recurrence, lymphatic invasion, and venous invasion. Furthermore, Fig. 3D graphically portrays the distribution of patients with distinct clinical characteristics across the three subtypes.

**Table 2:** Statistical comparison of clinical information of samples from different subtypes.

Characteristics	Cases (N = 438)	Subtypes			*P* value
		Subtype 1 (N = 215)	Subtype 2 (N = 93)	Subtype 3 (N = 130)	
Age(years)					0.111
≤60	134	67	35	32	
>60	304	148	58	98	
Gender					0.167
Male	233	124	47	62	
Female	205	91	46	68	
Pathologic M					**0.015**
M0	325	158	60	107	
M1	60	30	21	9	
Unspecified	53	27	12	14	
Pathologic N					**<0.001**
N0	257	127	37	93	
N1	103	55	22	26	
N2	78	33	34	11	
Pathologic T					**<0.001**
T1	11	7	0	4	
T2	76	42	3	31	
T3	300	148	72	80	
T4	50	18	18	14	
Unspecified	1	0	0	1	
Pathologic stage					**<0.001**
Stage I	74	39	3	32	
Stage II	168	77	33	58	
Stage III	126	63	34	29	
Stage IV	60	30	21	9	
Unspecified	10	6	2	2	
History of colon polyps					0.726
Yes	130	62	28	40	
No	242	125	50	67	
Unspecified	66	28	15	23	
Recurrence					**<0.001**
Yes	78	37	30	11	
No	293	145	52	96	
Unspecified	67	33	11	23	
Lymphatic invasion					**<0.001**
Yes	154	69	51	34	
No	241	120	35	86	
Unspecified	43	26	7	10	
Venous invasion					**<0.001**
Yes	90	32	35	23	
No	288	152	44	92	
Unspecified	60	31	14	15	

Bold *P* value < 0.05 indicates statistical significances.

### 3.4 Correlation analysis of subtypes and immune microenvironment

**Fig. 4:**
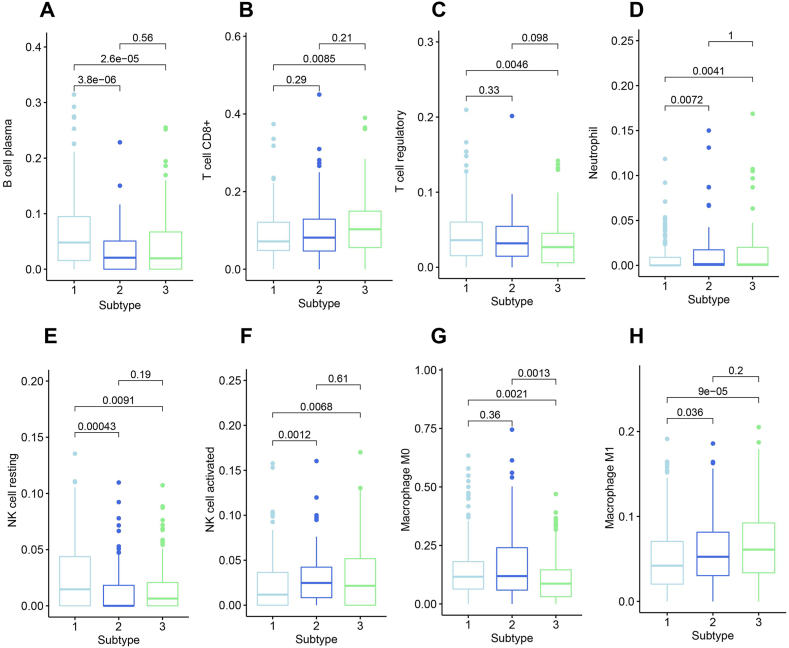
Correlation analysis of subtypes and immune cell infiltrations. The bar charts effectively illustrate notable variations in the proportions of plasma B cells (A), CD8^+^ T cells (B), regulatory T cells (C), neutrophils (D), NK resting cells (E), NK activated cells (F), M0 macrophages (G) and M1 macrophages (H) across the three identified subtypes.

The infiltration of 22 types of immune cells in COAD samples from TCGA training set was analyzed using the CIBERSORT algorithm, and the differences in immune cell proportions among the 3 subtypes were analyzed using the aov function, as shown in Table S2 and Fig. 4. In total, the proportions of 8 immune cell, including plasma B cells, CD8^+^ T cells, regulatory T cells, resting NK cells, activated NK cells, M0 macrophages, M1 macrophages and neutrophils, were significantly different among the 3 subtypes (P < 0.05).

### 3.5 Construction and validation of the prognostic model

**Fig. 5:**
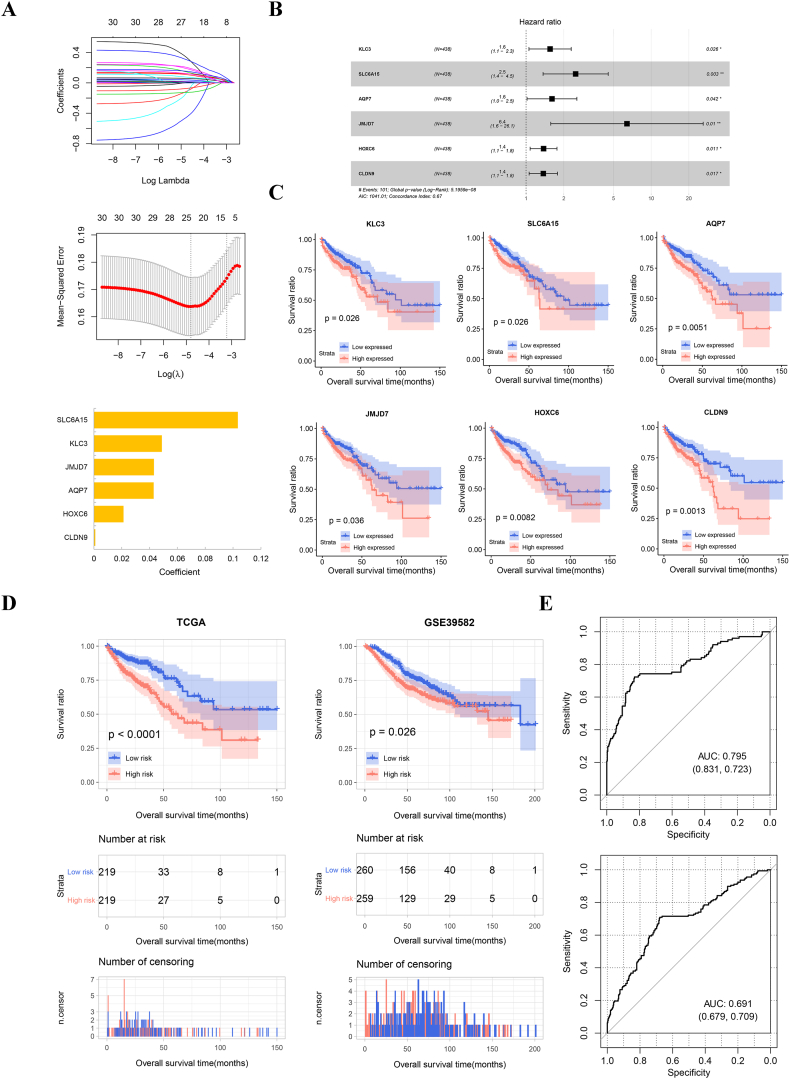
Establishment and verification of the PS-based prognostic model. A: Utilizing the LASSO algorithm with a regularization parameter of 1se = 0.04007, the optimal gene set was determined as the combination of the six-gene-signatures. B: The forest plot visually depicts the pronounced prognostic significance carried by the six-gene-signatures. C: The KM curves provide a visual representation of the intricate associations between the expressions of KLC3, SLC6A15, AQP7, JMJD7, HOXC6, and CLDN9, and the observed survival outcomes of COAD patients. D: KM curves effectively illustrate the interplay between the risk groups stratified by PS and the real survival outcomes among COAD patients across the TCGA and GSE39582 datasets. E: The ROC curves adeptly showcase the predictive prowess of the developed prognostic model, as reflected in its sensitivity and specificity, within both the TCGA and GSE39582 datasets.

Multivariate Cox regression analysis was then performed on 206 prognostic m6A-related DEGs, and 30 DEGs were selected with independent prognosis, as shown in Table S3. The six-gene-signature, including KLC3, SLC6A15, AQP7, JMJD7, HOXC6, and CLDN9, were determined as the optimal gene set by the LASSO algorithm (1se = 0.04007, Fig. 5A). Detailed information on these six-gene-signature is displayed in Table 3 and Fig. 5B, and they were considered as risk factors for COAD prognosis with hazard ratios all over 1. Afterwards, the correlations between the expression of the six-gene-signature and the survival prognosis of COAD patients were analyzed. The KM curves in Fig. 5C suggest that COAD patients with lower expression of these gene signatures had better survival ratios (P < 0.05).

**Table 3:** The information of 6 gene signatures.

Gene name	Hazard Ratio	95 % Confidence Interval	Standard error	Z score	*P*-value	LASSO Coefficient
*KLC3*	1.555	1.054-2.293	0.198	2.227	2.595E-02	0.04884
*SLC6A15*	2.488	1.366-4.530	0.306	2.979	2.900E-03	0.10343
*AQP7*	1.610	1.018-2.546	0.234	2.037	4.166E-02	0.04295
*JMJD7*	6.402	1.573-26.06	0.716	2.593	9.530E-03	0.04310
*HOXC6*	1.379	1.077-1.765	0.126	2.550	1.078E-02	0.02131
*CLDN9*	1.372	1.058-1.780	0.133	2.383	1.719E-02	0.00101

LASSO: least absolute shrinkage and selection operator.

Then, to verify the prognostic values of the six-gene-signature, patients from TCGA and GSE39582 datasets were first divided into high-risk and low-risk groups basing on median PS values. Survival analyses were then performed to further assess the relationship between the risk group and the actual outcomes of patients. The KM curves in Fig. 5D illustrate that the survival status of patients in the low-risk groups was significantly higher than that in the high-risk groups in both TCGA and GSE39582 datasets (P < 0.05). Receiver operator characteristic (ROC) curves were created to evaluate the sensitivity and specificity of the prognostic model. The results proved that the values of ROC were 0.795 and 0.691, respectively in both TCGA and GSE39582 datasets (Fig. 5E). Therefore, PS model has a good performance in predicting the survival prognosis of patients.

### 3.6 Analysis of m6A-regulatory network and subtype distributions

**Fig. 6:**
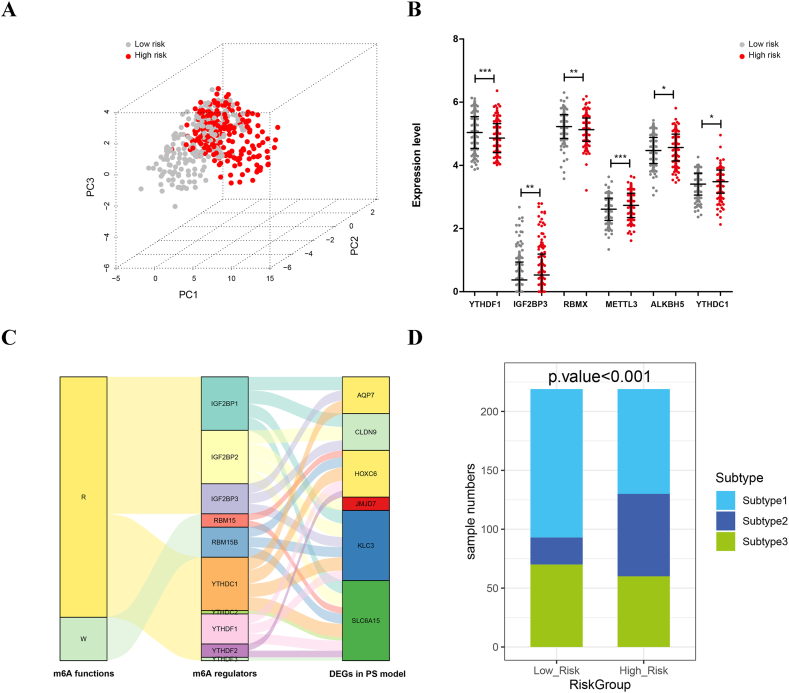
Analyses of the m6A regulatory network and subtype distributions among risk groups. A: The Principal Component Analysis (PCA) elucidates the divergence in distributions between high- and low-risk samples within TCGA, predicated upon the expression patterns of m6A-related genes. B: The dot distribution map visually underscores the contrasting expressions of m6A enzymes within the high- and low-risk groups. C: The regulatory network diagram delineates the intricate interconnections between m6A functionalities, m6A regulators, and the identified six-gene-signatures. D: The bar chart meticulously illustrates the partitioning of samples across the three subtypes within both high- and low-risk groups.

Principal component analysis (PCA) was performed to further observe whether patients at different risks had different m6A statuses. According to the scatter distribution in Fig. 6A, there is satisfactory dispersion between the two groups. Therefore, the low-risk and high-risk groups could be distinguished by the expression of m6A-related genes. Furthermore, it was found that the expression of 6 m6A enzymes, including YTHDF1, IGF2BP3, RBMX, METTL3, ALKBH5, and YTHDC1, was remarkably different between the low- and high-risk groups (Fig. 6B). To observe the relationship between m6A functions, m6A regulators and DEGs in PS model, a Sankey diagram was drawn. As shown in Figs. 6C and 8 m6A readers and 2 m6A writers were correlated with six-gene-signature. Finally, through a bar chart in Fig. 6D, we found that the sample distributions of the three subtypes were significant between the risk groups. The proportion of subtype 2 samples in high-risk group was significantly higher than that in low-risk group (P < 0.001), which was consistent with the verification results of survival analysis, and COAD of subtype 2 predicted poor survival status.

### 3.7 Establishment and verification of the nomogram prediction model

**Fig. 7:**
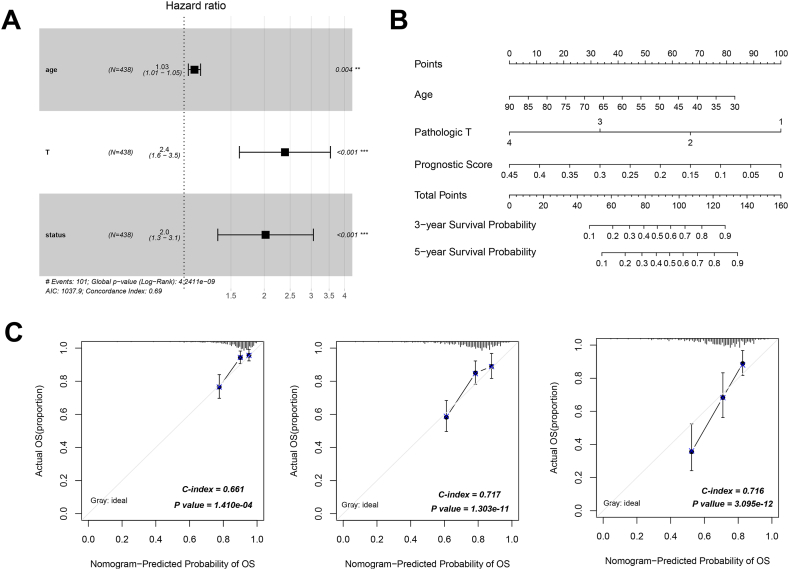
Establishment and validation of a nomogram prediction model. A: The independent prognostic factors were discerned through the utilization of univariate and multivariate Cox proportional hazards models. B: Building upon the foundation of three distinct independent prognostic factors, a nomogram model was constructed to predict the three- and five-year survival probabilities of patients with COAD. C: Calibration curves were meticulously generated to scrutinize the concordance between forecasted and observed one-, three-, and five-year survival rates, alongside the concurrent computation of C-indexes. The x-axis corresponds to the projected survival probability, while the y-axis corresponds to the factual survival status.

Incorporating the clinical information, we conducted univariate and multivariate Cox proportional hazards analyses to pinpoint independent prognostic determinants. The outcomes are delineated in Table 4 and Fig. 7A. Age, pathologic T, and PS status emerged as independently significant prognostic factors in both univariate and multivariate Cox proportional hazards regression evaluations. Drawing upon these three robust prognostic factors, we proceeded to devise a nomogram model to forecast the three- and five-year survival probabilities of COAD patients (Fig. 7B). As a conclusive step, calibration curves were generated and C-indexes were computed to rigorously assess the predictive performance of this nomogram model. Notably, the calibration curves displayed in Fig. 7C exhibit congruence between the projected one-, three-, and five-year survival rates and the actual survival rates, yielding C-indexes of 0.661, 0.717, and 0.716, respectively.

**Table 4:** Screening of independent prognostic factors by the univariate and multivariate Cox regression analysis.

Clinical characteristics	Uni-variable Cox		Multi-variable Cox	
	HR (95 % CI)	*P* value	HR (95 % CI)	*P* value
Age (years, mean ± sd)	1.022[1.005–1.039]	**7.580E-03**	1.034[1.013–1.055]	**1.470E-03**
Gender (Male/Female)	1.101[0.744–1.631]	6.302E-01	–	–
Pathologic M(M0/M1/-)	4.227[2.687–6.648]	**1.186E-11**	1.801[0.424–7.641]	4.251E-01
Pathologic N(N0/N1/-)	1.990[1.578–2.509]	**1.226E-09**	1.480[0.761–2.877]	2.483E-01
Pathologic T(T1/T2/T3/T4/-)	2.424[1.642–3.580]	**4.762E-06**	2.703[1.353–5.401]	**4.860E-03**
Pathologic stage (I/II/III/IV/-)	2.060[1.635–2.596]	**4.936E-10**	1.211[0.414–3.548]	7.266E-01
Recurrence (Yes/No/-)	2.544[1.625–3.981]	**2.319E-05**	1.724[0.919–3.235]	8.971E-02
History of colon polyps (Yes/No/-)	0.784[0.467–1.316]	3.498E-01	–	–
Lymphatic invasion (Yes/No/-)	2.267[1.485–3.459]	**9.831E-05**	0.923[0.437–1.950]	8.342E-01
Venous invasion (Yes/No/-)	2.496[1.616–3.856]	**1.997E-05**	1.082[0.503–2.328]	8.395E-01
PS status (High/Low)	2.222[1.474–3.351]	**8.822E-05**	1.825[1.073–3.104]	**2.632E-02**

SD: standard division; HR: hazard ratio; CI: confidence interval; PS: prognostic score.

Bold *P* value < 0.05 indicates statistical significances.

## 4 Discussion

Similar to DNA and histone methylation, m6A methylation is also dynamically invertible in mammalian cells and is considered another form of epigenetic regulation [38]. Differential expression of m6A regulators is correlated with abnormal RNA regulation in tumors. The prognostic potential of m6A regulators has been explored in bioinformatics analyses for both CRC and COAD [18,39]. However, the implications of m6A regulator target genes on COAD prognosis remain uncharted territory. In this study, harnessing clinical information from COAD patients, COAD DEGs, and publicly predicted m6A-related genes, we identified a total of 206 prognostic m6A-related DEGs that interact with eight m6A enzymes. Based on the expression levels of these prognostic m6A-related DEGs, COAD samples were stratified into three clusters, revealing substantial disparities in survival status, clinical traits, and immune cell infiltration. Subsequently, employing multivariate Cox and LASSO regression analyses, we distilled a six-gene signature that retained prognostic independence. Concomitantly, predictive models including PS and a nomogram were established, demonstrating robust efficacy in prognosticating the risk associated with COAD.

In this investigation, a cohort of 438 COAD samples from TCGA was stratified into three distinct subtypes, among which subtype 2 exhibited the most unfavorable survival profile, while subtype 3 displayed the most favorable. Concomitantly, an exploration of immune cell infiltration in these samples uncovered noteworthy trends. Specifically, a conspicuous reduction in M0 macrophage infiltration was observed in subtype 3 in comparison to subtypes 2 and 1, implying a potential association between diminished M0 macrophage infiltration and improved COAD prognosis. Furthermore, the infiltration levels of resting NK cells demonstrated a significant diminution in subtype 2 as opposed to subtypes 3 and 1, thus suggesting that reduced infiltration of resting NK cells might be a pivotal contributor to the poorer prognosis of COAD. Of interest, a competing endogenous RNA network analysis by Chang et al. unveiled a link between M0 macrophages, distant metastasis, and COAD prognosis [6]. This finding finds concurrence in a comprehensive bioinformatics assessment that discerned heightened M0 macrophage infiltration in cohorts with elevated immune cell infiltration scores, which in turn indicated a bleaker COAD prognosis [40]. Our current findings align with and further fortify these results, buttressing the assertion that an abundance of M0 macrophage infiltration is inversely correlated with COAD prognosis. Turning to NK cells, a related study disclosed that patients harboring an elevated prognostic risk for CRC exhibited markedly heightened infiltration of resting NK cells in comparison to those in the low prognostic risk group [41]. Moreover, a pan-cancer analysis illuminated a link between the upregulation of ANKRD6 and unfavorable CRC prognosis, with increased ANKRD6 expression corresponding to a conspicuous reduction in infiltration abundance of activated NK cells, particularly in the subgroup characterized by heightened ANKRD6 expression [42]. This comprehensive body of research lends credence to the hypothesis that subdued NK cell infiltration holds potential in prognosticating a dismal outlook for COAD.

The m6A is the most abundant epigenetic modification of RNA, and its imbalance can drive abnormal transcription and translation programs and promote the occurrence and development of tumors as well. In addition, m6A can also regulate the immunogenicity of tumors and immune cells involved in anti-tumor responses. For example, METTL3 in regulatory T(Treg) cells can reduce the stability of SOCS mRNA through m6A modification, activate IL-2/STAT5 signaling, inhibit the secretion of T cell effector cytokines, and reduce the anti-tumor response of CD8+T cells in TME [43]. Recent advances in mapping m6A in mRNAs, coupled with the ability to manipulate m6A deposition and recognize enzymes, have led to the emerging role of m6A modification patterns in immunotherapy. For example, Zhang et al. investigated the role of m6A modification features in predicting immune checkpoint block treatment response and discovered that patients with low m6A scores had significant clinical efficacy and significantly extended survival [44]. Further studies showed that regulatory T cells and TME matrix were significantly activated in tumors with high m6A score, mediating tumor immune tolerance. This study revealed the post-transcriptional regulation mode mediated by m6A methylation during the occurrence of COAD from the epigenetic level, providing a new research idea for the immunotherapy of COAD patients.

The six-gene signature was also screened to predict the prognostic risk of COAD in this study. Among them, KLC3, SLC6A15, AQP7, HOXC6, and CLDN9 were found to be related to three m6A regulators, namely IGF2BP3, YTHDC1, and YTHDF1 that were differentially expressed between the risk groups. CLDNs is a quad-panprotein associated with malignant features of cancer, such as invasion, migration, metastasis, and chemotherapy resistance. As a glycolysis-related gene, CLDN9 has also been identified as a predictor of poor prognosis in multiple tumors, such as endometrial cancer, esophageal adenocarcinoma, etc. [45,46] HOXC6 encodes a highly conserved transcription factor, and multivariate analysis showed that increased HOXC6 expression was an independent risk factor for poor prognosis in CRC patients [47]. HOXC6 is considered as a marker gene in the pathological evolution of CRC and can be used to predict the prognosis of CRC patients [48]. Our results also showed that COAD patients with high HOXC6 expression had a lower survival rate. To explain the regulatory mechanisms of HOXC6, Qi et al. pointed out that the overexpression of HOXC6 could promote the migration and invasion of CRC cells by activating the Wnt/β-catenin signaling pathway, inhibiting the secretion of DKK1 (an inhibitor of Wnt signaling), and inducing epithelial-mesenchymal transformation [49]. Furthermore, our study also identified potential regulatory relationships between HOXC6, YTHDC1, and YTHDF1. YTHDC1 was found to be differentially expressed between the high- and low-risk CRC prognostic groups [50], while YTHDF1was significantly upregulated in human colon cancer tumors and cell lines [51]. The YTH domain family of m6A readers is believed to be related to the clinical features of COAD patients and plays a crucial role in the prognosis of COAD [16]. Combined with our results, the abnormal regulation of YTHDF1 and YTHDC1 in COAD may be related to the expression of HOXC6. In a broader context, the dynamic and reversible nature of m6A modification remains a focal point, with many of its regulators awaiting elucidation. The prospect of harnessing m6A modifications for tumor immunotherapy presents notable complexities, notably the intricate interplay between "on-target, off-tumor" effects. Consequently, the comprehensive unveiling of m6A's biological attributes and the enhancement of our adeptness in manipulating this molecular landscape stand as formidable imperatives for the trajectory of forthcoming research endeavors.

Nonetheless, this study does have certain inherent limitations. Firstly, while the development of six novel COAD biomarkers has enabled the prognostic risk prediction through robust internal and external validation, their expression within solid tumor samples has yet to undergo experimental verification. Thus, a prudent avenue could involve in vivo validation at the animal level or clinical validation for more substantiated results. Secondly, despite proposing several conceivable regulatory interrelationships between m6A regulators and these gene attributes, the intricate molecular underpinnings remain enigmatic. Given the intricate intricacies and heterogeneity intrinsic to the immune microenvironment, we intend to embark on further mechanistic investigations to expound upon the intricate upstream and downstream regulatory dynamics.

## 5 Conclusion

The six-gene signature (comprising KLC3, SLC6A15, AQP7, JMJD7, HOXC6, and CLDN9) entwined within the m6A regulatory network presents a valuable tool for delineating the immune microenvironment across distinct COAD subtypes, while concurrently serving as a predictive instrument for assessing the prognostic risk among COAD patients.

## Availability of data and materials

The datasets generated and/or analyzed during the current study are available in the [TCGA and GEO] repository [www.tcga.org/and www.ncbi.nlm.nih.gov/geo/].

## Ethics approval and consent to participate

Not applicable.

## Consent for publication

Not applicable.

## Funding

This work was supported by the Public Welfare Technology Application Research Program of Huzhou (No.2021YZ30)，Zhejiang Medical and Health Technology Project (No.2024KY1636，No.2023KY313).

## CRediT authorship contribution statement

**Han Shugao:** Conceptualization, Writing – review & editing, Formal analysis, Validation. **Wu Yinhang:** Data curation, Formal analysis, Methodology, Validation. **Zhuang Jing:** Formal analysis, Visualization, Validation. **Qu Zhanbo:** Formal analysis, Validation, Visualization. **Da Miao:** Conceptualization, Writing – original draft, Writing – review & editing, Validation.

## Declaration of competing interest

The authors declare that they have no known competing financial interests or personal relationships that could have appeared to influence the work reported in this paper.

## List of abbreviations

CRC: colorectal cancer
COAD: colonic adenocarcinoma
m6A: N6-methyladenosine;
YTH: YT521-B homology
DEGs: differentially expressed genes
TCGA: The Cancer Genome Atlas
GEO: gene expression omnibus
FDR: false discovery rate
FC: fold change
KM: Kaplan–Meier
LASSO: least absolute shrinkage and selection operator
PS: prognostic score
PCA: principal component analysis
KEGG: Kyoto Encyclopedia of Genes and Genomes
ROC: receiver operator characteristic
