# Supplementary material for: Action of m6A-related gene signatures on the prognosis and immune microenvironment of colonic adenocarcinoma
Source: Heliyon. 2024 May 24;10(11):e31441. doi: 10.1016/j.heliyon.2024.e31441 (PMC11153101; doi:10.1016/j.heliyon.2024.e31441)
Supplement: Multimedia component 1 [file mmc1.docx]

| **Table S1 Expression levels of 2422 differential expressed genes between tumors and controls** | | | | |
| --- | --- | --- | --- | --- |
| **Symbol** | **sample_type** | **logFC** | **pvalue** | **FDR** |
| *DAO* | ENSG00000110887.6 | -4.892479849 | 7.55E-12 | 2.09E-11 |
| *SLC17A8* | ENSG00000179520.9 | -4.702432983 | 7.75E-09 | 1.64E-08 |
| *VSTM2A* | ENSG00000170419.9 | -4.526372451 | 1.27E-19 | 7.18E-19 |
| *PCSK2* | ENSG00000125851.8 | -4.493432812 | 2.97E-13 | 9.27E-13 |
| *SLC30A10* | ENSG00000196660.9 | -4.348051115 | 7.82E-15 | 2.82E-14 |
| *FAM135B* | ENSG00000147724.10 | -4.270078216 | 8.58E-19 | 4.45E-18 |
| *ABCB11* | ENSG00000073734.8 | -4.212753474 | 6.00E-09 | 1.29E-08 |
| *PMP2* | ENSG00000147588.6 | -4.209879532 | 4.32E-12 | 1.22E-11 |
| *NRXN1* | ENSG00000179915.19 | -4.154338937 | 1.84E-11 | 4.93E-11 |
| *MYOC* | ENSG00000034971.13 | -4.131128499 | 7.04E-17 | 3.06E-16 |
| *CMTM5* | ENSG00000166091.18 | -4.113726549 | 4.44E-09 | 9.62E-09 |
| *PHOX2B* | ENSG00000109132.6 | -4.065380254 | 2.77E-12 | 7.97E-12 |
| *DPP6* | ENSG00000130226.15 | -3.991015955 | 2.68E-06 | 4.60E-06 |
| *RERGL* | ENSG00000111404.5 | -3.988363249 | 9.52E-18 | 4.46E-17 |
| *STMN4* | ENSG00000015592.15 | -3.986515237 | 7.53E-12 | 2.09E-11 |
| *SGCG* | ENSG00000102683.7 | -3.971917793 | 8.07E-15 | 2.90E-14 |
| *TMEFF2* | ENSG00000144339.10 | -3.946918694 | 3.70E-12 | 1.05E-11 |
| *PLP1* | ENSG00000123560.12 | -3.893800799 | 1.34E-17 | 6.21E-17 |
| *ANGPTL7* | ENSG00000171819.4 | -3.860650532 | 5.17E-12 | 1.45E-11 |
| *XKR4* | ENSG00000206579.8 | -3.83764665 | 6.25E-11 | 1.59E-10 |
| *FRMPD4* | ENSG00000169933.11 | -3.829253397 | 0.00011631 | 0.00017548 |
| *CADM2* | ENSG00000175161.12 | -3.757469269 | 2.89E-10 | 6.96E-10 |
| *SLC5A7* | ENSG00000115665.7 | -3.700374864 | 2.40E-07 | 4.50E-07 |
| *ST8SIA3* | ENSG00000177511.5 | -3.671196234 | 4.62E-08 | 9.22E-08 |
| *EPHA6* | ENSG00000080224.16 | -3.64269077 | 0.000151294 | 0.000225904 |
| *FEV* | ENSG00000163497.2 | -3.609136075 | 2.17E-16 | 9.02E-16 |
| *ABCA8* | ENSG00000141338.12 | -3.564459296 | 7.96E-30 | 1.32E-28 |
| *HSD3B2* | ENSG00000203859.8 | -3.562534301 | 2.70E-09 | 5.98E-09 |
| *MUSK* | ENSG00000030304.11 | -3.561773302 | 3.20E-19 | 1.73E-18 |
| *SCN7A* | ENSG00000136546.12 | -3.553261111 | 3.61E-12 | 1.03E-11 |
| *RXRG* | ENSG00000143171.11 | -3.522992552 | 7.30E-19 | 3.81E-18 |
| *CNTN2* | ENSG00000184144.8 | -3.508288996 | 5.87E-10 | 1.38E-09 |
| *HS3ST6* | ENSG00000162040.6 | -3.483844043 | 3.23E-06 | 5.51E-06 |
| *SLC7A14* | ENSG00000013293.5 | -3.466806286 | 1.15E-08 | 2.41E-08 |
| *MGAT4C* | ENSG00000182050.12 | -3.441871256 | 4.99E-10 | 1.18E-09 |
| *P2RX2* | ENSG00000187848.11 | -3.440971098 | 5.91E-11 | 1.51E-10 |
| *TAT* | ENSG00000198650.9 | -3.437460473 | 7.19E-11 | 1.82E-10 |
| *ADCYAP1R1* | ENSG00000078549.13 | -3.416306043 | 7.31E-09 | 1.56E-08 |
| *CLDN8* | ENSG00000156284.5 | -3.410493354 | 7.72E-16 | 3.05E-15 |
| *BMP3* | ENSG00000152785.6 | -3.407160742 | 1.65E-27 | 2.19E-26 |
| *CDH19* | ENSG00000071991.7 | -3.366297875 | 4.38E-10 | 1.04E-09 |
| *PKHD1L1* | ENSG00000205038.10 | -3.358686195 | 8.64E-16 | 3.40E-15 |
| *RIMS4* | ENSG00000101098.11 | -3.337978252 | 1.86E-08 | 3.83E-08 |
| *PIRT* | ENSG00000233670.6 | -3.328281832 | 4.25E-15 | 1.57E-14 |
| *GCG* | ENSG00000115263.13 | -3.319880703 | 3.19E-18 | 1.56E-17 |
| *CNGB1* | ENSG00000070729.12 | -3.306248114 | 5.56E-08 | 1.10E-07 |
| *MYOT* | ENSG00000120729.8 | -3.295739744 | 1.38E-14 | 4.85E-14 |
| *KCNA1* | ENSG00000111262.4 | -3.295444736 | 0.000575496 | 0.000822608 |
| *ABCB5* | ENSG00000004846.15 | -3.283936556 | 2.66E-06 | 4.56E-06 |
| *RBFOX3* | ENSG00000167281.17 | -3.280052882 | 3.00E-05 | 4.74E-05 |
| *PRIMA1* | ENSG00000175785.11 | -3.276025073 | 2.79E-17 | 1.26E-16 |
| *MAMDC2* | ENSG00000165072.9 | -3.258863937 | 1.79E-22 | 1.38E-21 |
| *CADM3* | ENSG00000162706.11 | -3.252158317 | 4.17E-26 | 4.74E-25 |
| *CTNNA3* | ENSG00000183230.15 | -3.248939371 | 9.66E-06 | 1.59E-05 |
| *ASPA* | ENSG00000108381.9 | -3.242152393 | 8.61E-19 | 4.46E-18 |
| *SLC6A19* | ENSG00000174358.14 | -3.222555553 | 1.27E-11 | 3.44E-11 |
| *NLGN1* | ENSG00000169760.16 | -3.220270473 | 3.88E-06 | 6.57E-06 |
| *SEMA3E* | ENSG00000170381.11 | -3.220225046 | 8.05E-15 | 2.89E-14 |
| *CTNND2* | ENSG00000169862.17 | -3.217478505 | 1.03E-19 | 5.88E-19 |
| *PHOX2A* | ENSG00000165462.5 | -3.21642143 | 4.58E-12 | 1.29E-11 |
| *P2RY4* | ENSG00000186912.6 | -3.214892743 | 6.26E-08 | 1.23E-07 |
| *RSPO2* | ENSG00000147655.9 | -3.208123935 | 1.17E-16 | 4.98E-16 |
| *SCGN* | ENSG00000079689.12 | -3.203686655 | 9.96E-34 | 2.39E-32 |
| *MORN5* | ENSG00000185681.11 | -3.187690709 | 1.56E-06 | 2.73E-06 |
| *TEX11* | ENSG00000120498.12 | -3.186681842 | 3.37E-15 | 1.25E-14 |
| *ASTN1* | ENSG00000152092.14 | -3.185150631 | 1.05E-09 | 2.41E-09 |
| *SLITRK3* | ENSG00000121871.3 | -3.17785697 | 9.50E-10 | 2.19E-09 |
| *NPTX1* | ENSG00000171246.5 | -3.171723766 | 4.05E-07 | 7.46E-07 |
| *SPOCK3* | ENSG00000196104.9 | -3.163674833 | 7.51E-10 | 1.74E-09 |
| *FRRS1L* | ENSG00000260230.2 | -3.152118186 | 4.50E-08 | 8.99E-08 |
| *GPM6A* | ENSG00000150625.15 | -3.144152064 | 3.75E-08 | 7.53E-08 |
| *ATP1A2* | ENSG00000018625.13 | -3.141734046 | 1.16E-12 | 3.45E-12 |
| *NEFM* | ENSG00000104722.12 | -3.137935792 | 1.35E-08 | 2.81E-08 |
| *BRINP3* | ENSG00000162670.9 | -3.13624089 | 9.96E-21 | 6.33E-20 |
| *KCNB1* | ENSG00000158445.7 | -3.126704918 | 2.45E-06 | 4.22E-06 |
| *C2orf40* | ENSG00000119147.8 | -3.125943373 | 4.49E-16 | 1.82E-15 |
| *SCN11A* | ENSG00000168356.10 | -3.123825705 | 1.02E-11 | 2.79E-11 |
| *PI16* | ENSG00000164530.12 | -3.09815486 | 7.96E-23 | 6.36E-22 |
| *AMPD1* | ENSG00000116748.18 | -3.096285767 | 6.21E-16 | 2.48E-15 |
| *CASR* | ENSG00000036828.12 | -3.088946279 | 3.67E-08 | 7.37E-08 |
| *SMYD1* | ENSG00000115593.13 | -3.08619235 | 5.79E-05 | 8.94E-05 |
| *ELAVL3* | ENSG00000196361.8 | -3.07995457 | 1.21E-08 | 2.53E-08 |
| *SORCS1* | ENSG00000108018.14 | -3.079387229 | 1.58E-11 | 4.26E-11 |
| *DMRTA1* | ENSG00000176399.3 | -3.075353662 | 1.00E-17 | 4.68E-17 |
| *SCNN1G* | ENSG00000166828.2 | -3.066028758 | 1.58E-06 | 2.76E-06 |
| *MAB21L1* | ENSG00000180660.7 | -3.062691115 | 1.59E-11 | 4.28E-11 |
| *MAPK4* | ENSG00000141639.10 | -3.061381614 | 6.77E-07 | 1.22E-06 |
| *ELANE* | ENSG00000197561.5 | -3.058295939 | 6.46E-16 | 2.57E-15 |
| *GLP2R* | ENSG00000065325.11 | -3.056619393 | 7.54E-29 | 1.14E-27 |
| *CMA1* | ENSG00000092009.9 | -3.04870985 | 3.00E-20 | 1.80E-19 |
| *LONRF2* | ENSG00000170500.11 | -3.045573334 | 5.10E-07 | 9.31E-07 |
| *ENPP6* | ENSG00000164303.9 | -3.044019889 | 1.73E-20 | 1.07E-19 |
| *SST* | ENSG00000157005.3 | -3.03244434 | 1.44E-19 | 8.10E-19 |
| *PYY* | ENSG00000131096.9 | -3.030429115 | 1.12E-27 | 1.52E-26 |
| *CA7* | ENSG00000168748.12 | -3.028357912 | 3.37E-36 | 1.01E-34 |
| *INSL5* | ENSG00000172410.4 | -3.027953644 | 8.82E-08 | 1.71E-07 |
| *NRSN1* | ENSG00000152954.10 | -3.012017629 | 8.99E-07 | 1.61E-06 |
| *CNR1* | ENSG00000118432.12 | -3.003056262 | 9.42E-13 | 2.82E-12 |
| *SCN2B* | ENSG00000149575.5 | -2.996240362 | 3.05E-09 | 6.71E-09 |
| *CA1* | ENSG00000133742.12 | -2.993685851 | 7.30E-26 | 8.09E-25 |
| *PRPH* | ENSG00000135406.12 | -2.992647043 | 9.17E-16 | 3.59E-15 |
| *GFRA2* | ENSG00000168546.9 | -2.990846836 | 7.60E-24 | 6.75E-23 |
| *SLCO4C1* | ENSG00000173930.8 | -2.983101474 | 2.45E-11 | 6.49E-11 |
| *NPY* | ENSG00000122585.6 | -2.970705041 | 2.99E-13 | 9.33E-13 |
| *KRT222* | ENSG00000213424.7 | -2.949235123 | 3.36E-13 | 1.04E-12 |
| *OLFM3* | ENSG00000118733.15 | -2.929985565 | 3.61E-09 | 7.88E-09 |
| *KCTD4* | ENSG00000180332.6 | -2.921886612 | 4.63E-10 | 1.09E-09 |
| *BEST4* | ENSG00000142959.4 | -2.912594412 | 1.01E-30 | 1.85E-29 |
| *TPH1* | ENSG00000129167.8 | -2.910402621 | 1.22E-12 | 3.62E-12 |
| *BCHE* | ENSG00000114200.8 | -2.901928721 | 1.75E-14 | 6.10E-14 |
| *CNGA3* | ENSG00000144191.10 | -2.891672006 | 9.14E-08 | 1.77E-07 |
| *GBA3* | ENSG00000249948.5 | -2.888592007 | 6.99E-16 | 2.77E-15 |
| *SFRP1* | ENSG00000104332.10 | -2.88735539 | 2.51E-24 | 2.36E-23 |
| *ATCAY* | ENSG00000167654.16 | -2.886007215 | 2.71E-09 | 6.00E-09 |
| *TMEM151B* | ENSG00000178233.16 | -2.884455475 | 1.47E-09 | 3.33E-09 |
| *MOBP* | ENSG00000168314.16 | -2.878353471 | 2.96E-13 | 9.25E-13 |
| *TMEM72* | ENSG00000187783.10 | -2.877543182 | 2.19E-21 | 1.51E-20 |
| *SYT4* | ENSG00000132872.10 | -2.877187959 | 2.62E-08 | 5.34E-08 |
| *PDE6A* | ENSG00000132915.9 | -2.871958971 | 5.12E-17 | 2.25E-16 |
| *NAP1L2* | ENSG00000186462.8 | -2.868767187 | 1.99E-20 | 1.22E-19 |
| *CLVS2* | ENSG00000146352.11 | -2.857439344 | 1.13E-06 | 2.00E-06 |
| *PRKG2* | ENSG00000138669.8 | -2.856902313 | 3.20E-15 | 1.19E-14 |
| *ELAVL4* | ENSG00000162374.15 | -2.850953897 | 1.20E-10 | 2.98E-10 |
| *GCNT2* | ENSG00000111846.14 | -2.844209478 | 6.64E-29 | 1.01E-27 |
| *PTPRZ1* | ENSG00000106278.10 | -2.843519383 | 1.85E-07 | 3.50E-07 |
| *FMN2* | ENSG00000155816.18 | -2.843194788 | 1.48E-07 | 2.82E-07 |
| *SCN9A* | ENSG00000169432.13 | -2.836137522 | 9.89E-26 | 1.08E-24 |
| *GRIK3* | ENSG00000163873.8 | -2.83315259 | 2.28E-10 | 5.54E-10 |
| *FOXD3* | ENSG00000187140.5 | -2.819403926 | 4.26E-09 | 9.25E-09 |
| *SPIB* | ENSG00000269404.5 | -2.813031175 | 8.96E-30 | 1.47E-28 |
| *MEP1B* | ENSG00000141434.10 | -2.806367945 | 3.89E-10 | 9.28E-10 |
| *ANKS1B* | ENSG00000185046.17 | -2.80220906 | 1.79E-06 | 3.12E-06 |
| *IGSF11* | ENSG00000144847.11 | -2.801476858 | 3.81E-07 | 7.04E-07 |
| *KRT9* | ENSG00000171403.8 | -2.798696078 | 1.30E-06 | 2.29E-06 |
| *ADH1B* | ENSG00000196616.11 | -2.788036166 | 1.87E-31 | 3.64E-30 |
| *TMEM100* | ENSG00000166292.10 | -2.787638188 | 1.57E-25 | 1.68E-24 |
| *CHRM2* | ENSG00000181072.10 | -2.780060825 | 4.19E-06 | 7.08E-06 |
| *GUCA2B* | ENSG00000044012.3 | -2.777368505 | 3.65E-32 | 7.69E-31 |
| *FCRL4* | ENSG00000163518.9 | -2.768298734 | 1.29E-06 | 2.28E-06 |
| *PCDH10* | ENSG00000138650.8 | -2.75852217 | 2.75E-08 | 5.59E-08 |
| *ADRA1A* | ENSG00000120907.16 | -2.756624599 | 1.01E-09 | 2.32E-09 |
| *CPEB1* | ENSG00000214575.8 | -2.7543244 | 8.67E-09 | 1.83E-08 |
| *TAGLN3* | ENSG00000144834.11 | -2.752025856 | 6.29E-08 | 1.24E-07 |
| *NOVA1* | ENSG00000139910.18 | -2.751563762 | 2.06E-12 | 5.99E-12 |
| *SNAP91* | ENSG00000065609.13 | -2.751467 | 1.69E-10 | 4.15E-10 |
| *EPHA5* | ENSG00000145242.12 | -2.749332717 | 1.36E-07 | 2.60E-07 |
| *PDZRN4* | ENSG00000165966.13 | -2.745451524 | 5.98E-08 | 1.18E-07 |
| *HMGCLL1* | ENSG00000146151.11 | -2.732371719 | 3.44E-15 | 1.28E-14 |
| *HPSE2* | ENSG00000172987.11 | -2.724398146 | 5.93E-27 | 7.42E-26 |
| *ANGPTL1* | ENSG00000116194.11 | -2.721671706 | 3.44E-15 | 1.28E-14 |
| *ZBTB16* | ENSG00000109906.12 | -2.718776137 | 5.63E-07 | 1.02E-06 |
| *CNTFR* | ENSG00000122756.13 | -2.707461046 | 1.53E-20 | 9.48E-20 |
| *TNFRSF13B* | ENSG00000240505.7 | -2.703500405 | 4.72E-16 | 1.91E-15 |
| *SLC30A8* | ENSG00000164756.11 | -2.702691972 | 3.13E-11 | 8.22E-11 |
| *RASGEF1C* | ENSG00000146090.14 | -2.696735777 | 8.63E-11 | 2.17E-10 |
| *SLC17A7* | ENSG00000104888.8 | -2.696542733 | 2.20E-17 | 1.00E-16 |
| *CCBE1* | ENSG00000183287.12 | -2.696174528 | 5.53E-09 | 1.19E-08 |
| *TCEAL2* | ENSG00000184905.7 | -2.694785693 | 6.51E-09 | 1.39E-08 |
| *EPHA7* | ENSG00000135333.12 | -2.685438216 | 7.32E-09 | 1.56E-08 |
| *AGTR1* | ENSG00000144891.16 | -2.684111719 | 6.73E-08 | 1.32E-07 |
| *SFRP5* | ENSG00000120057.4 | -2.682265152 | 4.93E-08 | 9.81E-08 |
| *PLCXD3* | ENSG00000182836.8 | -2.664488911 | 2.71E-09 | 6.00E-09 |
| *ADH1A* | ENSG00000187758.6 | -2.661595055 | 8.27E-13 | 2.49E-12 |
| *SYT9* | ENSG00000170743.15 | -2.655964048 | 8.55E-09 | 1.81E-08 |
| *SLC27A6* | ENSG00000113396.11 | -2.649810344 | 2.67E-14 | 9.16E-14 |
| *LDB3* | ENSG00000122367.18 | -2.644281487 | 3.87E-08 | 7.76E-08 |
| *COL19A1* | ENSG00000082293.11 | -2.641864989 | 1.49E-10 | 3.67E-10 |
| *ABCG2* | ENSG00000118777.9 | -2.637240409 | 1.04E-18 | 5.34E-18 |
| *SLC25A34* | ENSG00000162461.7 | -2.62715083 | 4.58E-24 | 4.19E-23 |
| *LRAT* | ENSG00000121207.10 | -2.626952183 | 0.02072695 | 0.025910363 |
| *UNC5D* | ENSG00000156687.9 | -2.623396257 | 4.25E-09 | 9.23E-09 |
| *SCARA5* | ENSG00000168079.15 | -2.622019017 | 2.90E-40 | 1.18E-38 |
| *OGN* | ENSG00000106809.9 | -2.619679242 | 3.98E-23 | 3.28E-22 |
| *ZNF536* | ENSG00000198597.7 | -2.61364788 | 5.83E-20 | 3.42E-19 |
| *ANO5* | ENSG00000171714.10 | -2.606514244 | 5.92E-29 | 9.06E-28 |
| *FXYD1* | ENSG00000266964.4 | -2.592661079 | 8.99E-18 | 4.22E-17 |
| *SCRG1* | ENSG00000164106.6 | -2.587194809 | 8.45E-05 | 0.000128909 |
| *CASQ2* | ENSG00000118729.11 | -2.583842863 | 1.86E-10 | 4.55E-10 |
| *PTCHD1* | ENSG00000165186.10 | -2.581937709 | 5.49E-06 | 9.18E-06 |
| *RIC3* | ENSG00000166405.13 | -2.577371023 | 2.31E-08 | 4.72E-08 |
| *NR1H4* | ENSG00000012504.12 | -2.56960303 | 1.61E-11 | 4.34E-11 |
| *CD300LG* | ENSG00000161649.11 | -2.565695149 | 0.000169204 | 0.000251577 |
| *SLC5A11* | ENSG00000158865.11 | -2.558193941 | 4.32E-05 | 6.74E-05 |
| *NEFL* | ENSG00000277586.1 | -2.550934542 | 7.32E-12 | 2.03E-11 |
| *COL6A5* | ENSG00000172752.13 | -2.550439491 | 2.36E-05 | 3.76E-05 |
| *NOS1* | ENSG00000089250.17 | -2.545352524 | 0.000718131 | 0.001019379 |
| *SCG3* | ENSG00000104112.7 | -2.54350444 | 5.68E-19 | 2.99E-18 |
| *B4GALNT2* | ENSG00000167080.7 | -2.542932731 | 4.28E-19 | 2.28E-18 |
| *TINCR* | ENSG00000223573.5 | -2.533482157 | 4.61E-18 | 2.23E-17 |
| *LIFR* | ENSG00000113594.8 | -2.532016548 | 1.87E-20 | 1.15E-19 |
| *AFF3* | ENSG00000144218.17 | -2.528454491 | 5.13E-13 | 1.57E-12 |
| *KIF1A* | ENSG00000130294.13 | -2.524778442 | 8.69E-10 | 2.01E-09 |
| *ENAM* | ENSG00000132464.10 | -2.517779178 | 9.05E-13 | 2.72E-12 |
| *C16orf89* | ENSG00000153446.14 | -2.510515398 | 1.25E-24 | 1.21E-23 |
| *HTR4* | ENSG00000164270.16 | -2.508167301 | 1.50E-18 | 7.59E-18 |
| *ZMAT4* | ENSG00000165061.13 | -2.506959724 | 4.31E-05 | 6.72E-05 |
| *ABCA9* | ENSG00000154258.15 | -2.502752016 | 3.80E-12 | 1.08E-11 |
| *NRG2* | ENSG00000158458.18 | -2.502027899 | 2.51E-08 | 5.12E-08 |
| *PPP1R1A* | ENSG00000135447.15 | -2.493554554 | 9.56E-07 | 1.71E-06 |
| *CBLN2* | ENSG00000141668.8 | -2.491384249 | 2.30E-16 | 9.55E-16 |
| *PYGM* | ENSG00000068976.12 | -2.488934336 | 1.47E-08 | 3.06E-08 |
| *TNXB* | ENSG00000168477.16 | -2.485332446 | 2.89E-17 | 1.30E-16 |
| *KCNS2* | ENSG00000156486.7 | -2.475143987 | 5.77E-07 | 1.05E-06 |
| *NTNG1* | ENSG00000162631.17 | -2.462556049 | 0.002322844 | 0.003161865 |
| *BEND4* | ENSG00000188848.14 | -2.461371807 | 3.24E-10 | 7.78E-10 |
| *LMO3* | ENSG00000048540.13 | -2.457700465 | 8.02E-08 | 1.56E-07 |
| *CWH43* | ENSG00000109182.10 | -2.44969516 | 5.51E-24 | 4.98E-23 |
| *SLITRK2* | ENSG00000185985.8 | -2.447642123 | 6.86E-11 | 1.74E-10 |
| *GSG1L* | ENSG00000169181.11 | -2.445341428 | 2.73E-14 | 9.36E-14 |
| *AQP8* | ENSG00000103375.9 | -2.438164355 | 3.96E-21 | 2.65E-20 |
| *PCOLCE2* | ENSG00000163710.6 | -2.436926297 | 1.59E-16 | 6.68E-16 |
| *C1QTNF9* | ENSG00000240654.5 | -2.430721925 | 2.42E-13 | 7.62E-13 |
| *FCER2* | ENSG00000104921.13 | -2.430478944 | 1.39E-09 | 3.16E-09 |
| *GRIN2A* | ENSG00000183454.12 | -2.428609523 | 1.23E-13 | 3.97E-13 |
| *CNTN1* | ENSG00000018236.13 | -2.425025094 | 1.99E-13 | 6.31E-13 |
| *B3GALT1* | ENSG00000172318.5 | -2.413172961 | 3.72E-23 | 3.08E-22 |
| *TMEM59L* | ENSG00000105696.7 | -2.409292633 | 2.41E-13 | 7.59E-13 |
| *LYVE1* | ENSG00000133800.7 | -2.399617822 | 1.06E-22 | 8.39E-22 |
| *CELA3B* | ENSG00000219073.6 | -2.397421619 | 3.23E-05 | 5.09E-05 |
| *HAND1* | ENSG00000113196.2 | -2.390804281 | 1.67E-06 | 2.91E-06 |
| *IGSF10* | ENSG00000152580.8 | -2.385636562 | 2.78E-12 | 7.99E-12 |
| *KCNK3* | ENSG00000171303.6 | -2.385120814 | 2.31E-09 | 5.15E-09 |
| *DCLK1* | ENSG00000133083.13 | -2.383824406 | 4.06E-13 | 1.25E-12 |
| *WSCD2* | ENSG00000075035.8 | -2.382177847 | 2.10E-07 | 3.96E-07 |
| *INA* | ENSG00000148798.8 | -2.380979513 | 3.54E-11 | 9.27E-11 |
| *FSIP2* | ENSG00000188738.12 | -2.375245689 | 7.80E-15 | 2.81E-14 |
| *CRHBP* | ENSG00000145708.9 | -2.36962132 | 3.01E-17 | 1.35E-16 |
| *USP2* | ENSG00000036672.14 | -2.368229359 | 5.10E-20 | 3.01E-19 |
| *ALPI* | ENSG00000163295.4 | -2.366799538 | 1.20E-20 | 7.57E-20 |
| *SPINK2* | ENSG00000128040.9 | -2.366636062 | 8.58E-17 | 3.70E-16 |
| *LCN10* | ENSG00000187922.12 | -2.36446183 | 1.86E-05 | 2.99E-05 |
| *CNTNAP3* | ENSG00000106714.16 | -2.363022694 | 3.24E-07 | 6.02E-07 |
| *AICDA* | ENSG00000111732.9 | -2.359246588 | 0.010662814 | 0.013673085 |
| *CXCR5* | ENSG00000160683.4 | -2.358423337 | 4.00E-05 | 6.26E-05 |
| *PKNOX2* | ENSG00000165495.14 | -2.35824269 | 2.10E-21 | 1.45E-20 |
| *PENK* | ENSG00000181195.9 | -2.355743217 | 0.00013716 | 0.000205713 |
| *MAL* | ENSG00000172005.9 | -2.354336327 | 5.96E-18 | 2.84E-17 |
| *KCNE2* | ENSG00000159197.3 | -2.353928312 | 5.25E-14 | 1.75E-13 |
| *GRIA4* | ENSG00000152578.11 | -2.351214837 | 1.96E-06 | 3.40E-06 |
| *MYPN* | ENSG00000138347.14 | -2.351132446 | 1.57E-09 | 3.55E-09 |
| *ACSM5* | ENSG00000183549.9 | -2.349240792 | 3.06E-11 | 8.04E-11 |
| *CD177* | ENSG00000204936.8 | -2.348126765 | 5.96E-22 | 4.36E-21 |
| *CALY* | ENSG00000130643.7 | -2.344258589 | 8.44E-09 | 1.79E-08 |
| *ADHFE1* | ENSG00000147576.14 | -2.343778248 | 4.10E-18 | 1.99E-17 |
| *ATRNL1* | ENSG00000107518.15 | -2.339389115 | 0.000172057 | 0.000255697 |
| *NEUROD1* | ENSG00000162992.3 | -2.334675611 | 9.51E-09 | 2.00E-08 |
| *LRRN2* | ENSG00000170382.10 | -2.33287925 | 2.15E-19 | 1.19E-18 |
| *HRK* | ENSG00000135116.8 | -2.331325185 | 5.14E-09 | 1.11E-08 |
| *GSTM5* | ENSG00000134201.9 | -2.328095079 | 1.31E-18 | 6.65E-18 |
| *TRPM6* | ENSG00000119121.20 | -2.322688006 | 1.59E-19 | 8.89E-19 |
| *CHGA* | ENSG00000100604.11 | -2.320693887 | 5.48E-38 | 1.87E-36 |
| *GAP43* | ENSG00000172020.11 | -2.318595511 | 2.39E-14 | 8.24E-14 |
| *WDR17* | ENSG00000150627.14 | -2.318062952 | 7.08E-08 | 1.39E-07 |
| *TRPV3* | ENSG00000167723.13 | -2.315712737 | 1.07E-10 | 2.67E-10 |
| *GREM2* | ENSG00000180875.4 | -2.315382541 | 5.08E-34 | 1.25E-32 |
| *SLC4A4* | ENSG00000080493.12 | -2.31041157 | 6.70E-35 | 1.78E-33 |
| *P2RY12* | ENSG00000169313.9 | -2.306432661 | 3.95E-14 | 1.34E-13 |
| *DUSP26* | ENSG00000133878.7 | -2.299872642 | 5.51E-21 | 3.60E-20 |
| *CTSG* | ENSG00000100448.3 | -2.29908281 | 1.76E-26 | 2.10E-25 |
| *VIT* | ENSG00000205221.11 | -2.299015699 | 2.49E-13 | 7.84E-13 |
| *PCP4L1* | ENSG00000248485.1 | -2.29892896 | 1.31E-06 | 2.31E-06 |
| *CLCNKB* | ENSG00000184908.16 | -2.296824052 | 0.004763185 | 0.006311965 |
| *SLC17A1* | ENSG00000124568.9 | -2.296801107 | 2.91E-11 | 7.66E-11 |
| *SNAP25* | ENSG00000132639.11 | -2.293848207 | 3.54E-09 | 7.74E-09 |
| *CYP4B1* | ENSG00000142973.11 | -2.291118517 | 8.41E-07 | 1.51E-06 |
| *ANK2* | ENSG00000145362.15 | -2.290922575 | 4.22E-13 | 1.30E-12 |
| *ATP2B3* | ENSG00000067842.16 | -2.288964977 | 5.31E-05 | 8.22E-05 |
| *ASPG* | ENSG00000166183.14 | -2.287570976 | 4.35E-10 | 1.03E-09 |
| *PGR* | ENSG00000082175.13 | -2.285415714 | 1.10E-07 | 2.12E-07 |
| *GPR15* | ENSG00000154165.4 | -2.28455165 | 1.10E-21 | 7.84E-21 |
| *PDZD4* | ENSG00000067840.11 | -2.282877132 | 4.59E-14 | 1.54E-13 |
| *CHODL* | ENSG00000154645.12 | -2.281470429 | 2.75E-13 | 8.63E-13 |
| *RPH3A* | ENSG00000089169.13 | -2.280172538 | 1.69E-09 | 3.81E-09 |
| *ARHGAP20* | ENSG00000137727.11 | -2.273905151 | 5.42E-21 | 3.55E-20 |
| *CLEC4G* | ENSG00000182566.11 | -2.271149935 | 2.50E-06 | 4.30E-06 |
| *GALNT16* | ENSG00000100626.15 | -2.270971013 | 9.15E-12 | 2.52E-11 |
| *NWD2* | ENSG00000174145.7 | -2.269353578 | 6.38E-07 | 1.16E-06 |
| *RELN* | ENSG00000189056.12 | -2.268134662 | 1.79E-13 | 5.70E-13 |
| *FGFBP2* | ENSG00000137441.7 | -2.263872758 | 3.56E-11 | 9.32E-11 |
| *MS4A12* | ENSG00000071203.8 | -2.262133441 | 5.55E-24 | 5.01E-23 |
| *ABCA6* | ENSG00000154262.11 | -2.259867084 | 5.98E-14 | 1.99E-13 |
| *BEST2* | ENSG00000039987.5 | -2.257161874 | 1.89E-20 | 1.16E-19 |
| *NPAS4* | ENSG00000174576.7 | -2.252113062 | 3.49E-05 | 5.49E-05 |
| *FNDC9* | ENSG00000172568.4 | -2.250042269 | 2.13E-07 | 4.01E-07 |
| *VWC2* | ENSG00000188730.4 | -2.248502612 | 7.29E-12 | 2.02E-11 |
| *CA10* | ENSG00000154975.12 | -2.247532575 | 1.24E-14 | 4.38E-14 |
| *SVOPL* | ENSG00000157703.14 | -2.244047356 | 6.41E-08 | 1.26E-07 |
| *HPGDS* | ENSG00000163106.9 | -2.242372135 | 9.21E-23 | 7.32E-22 |
| *RBM20* | ENSG00000203867.7 | -2.241996176 | 2.93E-06 | 5.02E-06 |
| *LMX1A* | ENSG00000162761.13 | -2.241638818 | 4.59E-11 | 1.19E-10 |
| *CELF4* | ENSG00000101489.17 | -2.239752613 | 7.41E-08 | 1.45E-07 |
| *STOX2* | ENSG00000173320.8 | -2.236890313 | 1.13E-17 | 5.27E-17 |
| *MASP1* | ENSG00000127241.15 | -2.236478105 | 5.56E-07 | 1.01E-06 |
| *EMILIN3* | ENSG00000183798.4 | -2.232411701 | 2.27E-10 | 5.52E-10 |
| *C1QTNF7* | ENSG00000163145.11 | -2.230608041 | 4.63E-15 | 1.70E-14 |
| *CHGB* | ENSG00000089199.8 | -2.228119038 | 7.55E-19 | 3.93E-18 |
| *CLCA4* | ENSG00000016602.9 | -2.227996296 | 1.39E-28 | 2.05E-27 |
| *SULT1A2* | ENSG00000197165.9 | -2.22561368 | 9.17E-22 | 6.60E-21 |
| *SYNDIG1L* | ENSG00000183379.7 | -2.222485132 | 2.90E-09 | 6.40E-09 |
| *CUX2* | ENSG00000111249.12 | -2.221551454 | 2.54E-07 | 4.76E-07 |
| *TRPC7* | ENSG00000069018.16 | -2.217515456 | 4.16E-16 | 1.69E-15 |
| *FAM107A* | ENSG00000168309.15 | -2.217284244 | 5.38E-22 | 3.96E-21 |
| *NECAB1* | ENSG00000123119.10 | -2.216006896 | 4.31E-06 | 7.27E-06 |
| *SNTG2* | ENSG00000172554.10 | -2.21437845 | 1.57E-12 | 4.61E-12 |
| *ADRB3* | ENSG00000188778.4 | -2.213484012 | 1.16E-05 | 1.89E-05 |
| *ADAMTSL3* | ENSG00000156218.11 | -2.209378152 | 4.38E-10 | 1.04E-09 |
| *GFRA3* | ENSG00000146013.9 | -2.204835863 | 7.25E-17 | 3.14E-16 |
| *GFRA1* | ENSG00000151892.13 | -2.203790756 | 2.95E-15 | 1.10E-14 |
| *GCSAML* | ENSG00000169224.11 | -2.198336973 | 1.03E-14 | 3.67E-14 |
| *DPEP3* | ENSG00000141096.4 | -2.196578622 | 1.03E-06 | 1.83E-06 |
| *DIRAS2* | ENSG00000165023.5 | -2.191606285 | 1.22E-09 | 2.78E-09 |
| *GNAO1* | ENSG00000087258.12 | -2.190014224 | 6.71E-11 | 1.71E-10 |
| *HAND2* | ENSG00000164107.8 | -2.180606227 | 1.11E-09 | 2.54E-09 |
| *CCL23* | ENSG00000274736.3 | -2.179391209 | 1.01E-18 | 5.20E-18 |
| *ATP13A4* | ENSG00000127249.13 | -2.173965667 | 5.94E-15 | 2.16E-14 |
| *CNTN3* | ENSG00000113805.8 | -2.170977856 | 7.66E-23 | 6.15E-22 |
| *LIPC* | ENSG00000166035.9 | -2.168985321 | 4.82E-13 | 1.48E-12 |
| *MGAM* | ENSG00000257335.7 | -2.168717497 | 0.015322448 | 0.019390965 |
| *GPR88* | ENSG00000181656.6 | -2.165144136 | 4.15E-05 | 6.48E-05 |
| *HTR7* | ENSG00000148680.14 | -2.159645122 | 1.14E-16 | 4.85E-16 |
| *DPYSL5* | ENSG00000157851.15 | -2.157250094 | 1.57E-06 | 2.75E-06 |
| *SEC14L5* | ENSG00000103184.10 | -2.153995667 | 4.18E-12 | 1.18E-11 |
| *DNER* | ENSG00000187957.7 | -2.147721556 | 4.77E-10 | 1.13E-09 |
| *MMRN1* | ENSG00000138722.8 | -2.14730289 | 4.75E-20 | 2.81E-19 |
| *SCNN1B* | ENSG00000168447.9 | -2.144804926 | 1.41E-25 | 1.51E-24 |
| *STMN2* | ENSG00000104435.12 | -2.144529179 | 2.53E-36 | 7.64E-35 |
| *MYOM1* | ENSG00000101605.11 | -2.14379521 | 2.17E-11 | 5.78E-11 |
| *BEX1* | ENSG00000133169.5 | -2.142081769 | 7.50E-15 | 2.71E-14 |
| *LUZP2* | ENSG00000187398.10 | -2.1410841 | 2.63E-17 | 1.19E-16 |
| *HTR3A* | ENSG00000166736.10 | -2.139006702 | 1.12E-09 | 2.56E-09 |
| *ALK* | ENSG00000171094.14 | -2.137186332 | 4.85E-06 | 8.15E-06 |
| *CYP3A4* | ENSG00000160868.13 | -2.135308299 | 0.000458788 | 0.000661103 |
| *SLC51A* | ENSG00000163959.8 | -2.133589888 | 3.84E-12 | 1.09E-11 |
| *PKD1L2* | ENSG00000166473.15 | -2.130476429 | 4.33E-06 | 7.31E-06 |
| *TNNT3* | ENSG00000130595.15 | -2.126934203 | 2.84E-10 | 6.85E-10 |
| *NEGR1* | ENSG00000172260.12 | -2.125869153 | 1.84E-14 | 6.40E-14 |
| *LEP* | ENSG00000174697.4 | -2.124508814 | 0.005464684 | 0.007206412 |
| *PEG3* | ENSG00000198300.11 | -2.117138507 | 1.33E-09 | 3.03E-09 |
| *CD160* | ENSG00000117281.14 | -2.117133431 | 6.18E-11 | 1.58E-10 |
| *OMD* | ENSG00000127083.7 | -2.114956466 | 3.78E-13 | 1.17E-12 |
| *CREB3L3* | ENSG00000060566.12 | -2.1125467 | 6.29E-08 | 1.24E-07 |
| *RNF150* | ENSG00000170153.9 | -2.111429405 | 3.32E-08 | 6.69E-08 |
| *CA4* | ENSG00000167434.8 | -2.110109817 | 1.20E-31 | 2.39E-30 |
| *TLL1* | ENSG00000038295.6 | -2.108330493 | 3.22E-14 | 1.10E-13 |
| *ACKR2* | ENSG00000144648.13 | -2.104014398 | 2.08E-18 | 1.04E-17 |
| *DAND5* | ENSG00000179284.5 | -2.099841135 | 0.000300529 | 0.000438737 |
| *TCL1A* | ENSG00000100721.9 | -2.095658818 | 9.54E-06 | 1.57E-05 |
| *DSCAML1* | ENSG00000177103.12 | -2.088960302 | 2.09E-09 | 4.68E-09 |
| *GRIK1* | ENSG00000171189.15 | -2.08833486 | 9.82E-11 | 2.46E-10 |
| *ANO3* | ENSG00000134343.11 | -2.087184003 | 1.30E-05 | 2.11E-05 |
| *KCNG3* | ENSG00000171126.7 | -2.08675709 | 1.42E-12 | 4.19E-12 |
| *SEMA3D* | ENSG00000153993.12 | -2.081865737 | 1.04E-18 | 5.34E-18 |
| *NCAM1* | ENSG00000149294.15 | -2.080764305 | 6.86E-14 | 2.26E-13 |
| *SSTR2* | ENSG00000180616.7 | -2.078675218 | 2.16E-12 | 6.27E-12 |
| *SIGLEC11* | ENSG00000161640.14 | -2.069477826 | 2.82E-11 | 7.43E-11 |
| *GFI1B* | ENSG00000165702.11 | -2.066810775 | 3.06E-16 | 1.26E-15 |
| *TRIM9* | ENSG00000100505.12 | -2.061239059 | 9.66E-11 | 2.42E-10 |
| *C7* | ENSG00000112936.17 | -2.059025525 | 1.10E-19 | 6.26E-19 |
| *GRIA3* | ENSG00000125675.16 | -2.058453213 | 5.60E-20 | 3.28E-19 |
| *ERBB4* | ENSG00000178568.12 | -2.054640876 | 7.05E-05 | 0.000108181 |
| *ABI3BP* | ENSG00000154175.15 | -2.054404886 | 4.76E-20 | 2.82E-19 |
| *PAPPA2* | ENSG00000116183.9 | -2.043895882 | 4.86E-06 | 8.16E-06 |
| *FCRLA* | ENSG00000132185.15 | -2.043527225 | 1.89E-09 | 4.24E-09 |
| *PIANP* | ENSG00000139200.12 | -2.042554114 | 6.14E-17 | 2.68E-16 |
| *SEZ6L* | ENSG00000100095.17 | -2.041852327 | 3.30E-07 | 6.13E-07 |
| *CHL1* | ENSG00000134121.8 | -2.035521877 | 2.35E-19 | 1.29E-18 |
| *TACR1* | ENSG00000115353.9 | -2.034124524 | 5.14E-07 | 9.38E-07 |
| *PLIN4* | ENSG00000167676.3 | -2.033267551 | 1.36E-08 | 2.83E-08 |
| *GRIK5* | ENSG00000105737.8 | -2.033170451 | 2.45E-08 | 5.00E-08 |
| *RGS13* | ENSG00000127074.13 | -2.021464448 | 2.10E-08 | 4.31E-08 |
| *SPX* | ENSG00000134548.8 | -2.021443344 | 1.38E-05 | 2.24E-05 |
| *DNASE1L3* | ENSG00000163687.12 | -2.021321816 | 4.20E-21 | 2.80E-20 |
| *TMEM130* | ENSG00000166448.13 | -2.02072347 | 7.08E-09 | 1.51E-08 |
| *TRHDE* | ENSG00000072657.7 | -2.020181827 | 1.45E-13 | 4.65E-13 |
| *UNC80* | ENSG00000144406.17 | -2.013708492 | 4.64E-05 | 7.22E-05 |
| *GNG13* | ENSG00000127588.4 | -2.012146132 | 4.60E-11 | 1.19E-10 |
| *KRT1* | ENSG00000167768.4 | -2.011440577 | 1.61E-06 | 2.81E-06 |
| *CILP* | ENSG00000138615.5 | -2.00875146 | 1.19E-15 | 4.60E-15 |
| *CPB1* | ENSG00000153002.10 | -2.006949774 | 0.020280246 | 0.02536835 |
| *DRD5* | ENSG00000169676.5 | -1.999728409 | 0.002090392 | 0.002859544 |
| *FAM181B* | ENSG00000182103.4 | -1.999477409 | 1.85E-10 | 4.53E-10 |
| *NTRK3* | ENSG00000140538.15 | -1.998538164 | 9.14E-09 | 1.93E-08 |
| *KLB* | ENSG00000134962.6 | -1.990414013 | 9.81E-17 | 4.20E-16 |
| *KIF5A* | ENSG00000155980.10 | -1.985219472 | 5.25E-11 | 1.35E-10 |
| *SIGLEC6* | ENSG00000105492.14 | -1.98515027 | 6.35E-16 | 2.53E-15 |
| *ADCY5* | ENSG00000173175.13 | -1.982345673 | 1.23E-19 | 6.96E-19 |
| *SCN3A* | ENSG00000153253.14 | -1.981133234 | 2.53E-18 | 1.25E-17 |
| *CNTN4* | ENSG00000144619.13 | -1.978886233 | 4.10E-22 | 3.06E-21 |
| *SSTR3* | ENSG00000278195.1 | -1.978291746 | 6.03E-11 | 1.54E-10 |
| *LAMA1* | ENSG00000101680.12 | -1.977194501 | 3.24E-13 | 1.01E-12 |
| *KY* | ENSG00000174611.10 | -1.976292817 | 0.003510843 | 0.004703222 |
| *CASQ1* | ENSG00000143318.11 | -1.975833387 | 0.000149721 | 0.000223771 |
| *CRB1* | ENSG00000134376.13 | -1.974338876 | 5.28E-06 | 8.84E-06 |
| *CP* | ENSG00000047457.12 | -1.97124755 | 1.33E-17 | 6.17E-17 |
| *BAALC* | ENSG00000164929.15 | -1.969117866 | 1.46E-16 | 6.16E-16 |
| *HSPB3* | ENSG00000169271.2 | -1.968475193 | 1.99E-09 | 4.46E-09 |
| *CNKSR2* | ENSG00000149970.13 | -1.964052914 | 1.71E-17 | 7.83E-17 |
| *ASXL3* | ENSG00000141431.8 | -1.963218702 | 3.25E-16 | 1.33E-15 |
| *CCDC158* | ENSG00000163749.16 | -1.960161437 | 2.90E-13 | 9.07E-13 |
| *LIX1* | ENSG00000145721.10 | -1.958101198 | 0.000858556 | 0.001212199 |
| *EFHC2* | ENSG00000183690.12 | -1.954859396 | 2.62E-19 | 1.43E-18 |
| *GRIA1* | ENSG00000155511.16 | -1.952895312 | 9.56E-10 | 2.20E-09 |
| *STAB2* | ENSG00000136011.13 | -1.950199891 | 9.87E-13 | 2.95E-12 |
| *ERVFRD-1* | ENSG00000244476.2 | -1.948408449 | 2.39E-11 | 6.34E-11 |
| *CNR2* | ENSG00000188822.7 | -1.948142773 | 3.79E-06 | 6.42E-06 |
| *LILRB5* | ENSG00000105609.15 | -1.944594623 | 1.08E-22 | 8.54E-22 |
| *BLK* | ENSG00000136573.11 | -1.941763138 | 6.16E-09 | 1.32E-08 |
| *ATP6V1G2* | ENSG00000213760.9 | -1.934555665 | 3.87E-10 | 9.23E-10 |
| *ABCC8* | ENSG00000006071.10 | -1.932647851 | 8.94E-11 | 2.25E-10 |
| *GNG8* | ENSG00000167414.4 | -1.932514403 | 5.54E-15 | 2.02E-14 |
| *ANXA8L1* | ENSG00000264230.6 | -1.928236801 | 0.001614699 | 0.002226149 |
| *RNF152* | ENSG00000176641.9 | -1.928148448 | 1.37E-21 | 9.64E-21 |
| *POU3F3* | ENSG00000198914.2 | -1.926545396 | 2.83E-07 | 5.28E-07 |
| *CHAD* | ENSG00000136457.8 | -1.91974954 | 5.54E-16 | 2.23E-15 |
| *PNCK* | ENSG00000130822.14 | -1.918749056 | 2.14E-05 | 3.42E-05 |
| *SH2D6* | ENSG00000152292.15 | -1.91748937 | 1.74E-10 | 4.27E-10 |
| *SLC17A4* | ENSG00000146039.9 | -1.915617789 | 7.68E-21 | 4.95E-20 |
| *WSCD1* | ENSG00000179314.12 | -1.913780201 | 1.68E-24 | 1.60E-23 |
| *GDPD2* | ENSG00000130055.12 | -1.913349786 | 4.20E-21 | 2.80E-20 |
| *C11orf86* | ENSG00000173237.4 | -1.91153597 | 8.12E-26 | 8.93E-25 |
| *COL4A6* | ENSG00000197565.14 | -1.907842093 | 7.27E-17 | 3.15E-16 |
| *EYA2* | ENSG00000064655.17 | -1.906828079 | 5.15E-18 | 2.47E-17 |
| *FCRL2* | ENSG00000132704.14 | -1.90625911 | 4.06E-11 | 1.06E-10 |
| *FCRL1* | ENSG00000163534.13 | -1.904841148 | 7.70E-06 | 1.27E-05 |
| *UNC5C* | ENSG00000182168.13 | -1.895746101 | 6.54E-21 | 4.25E-20 |
| *GRIA2* | ENSG00000120251.17 | -1.894249184 | 1.68E-05 | 2.71E-05 |
| *MYOCD* | ENSG00000141052.16 | -1.893793151 | 1.99E-10 | 4.85E-10 |
| *TM6SF2* | ENSG00000213996.11 | -1.892985451 | 1.34E-09 | 3.05E-09 |
| *ZNF229* | ENSG00000278318.3 | -1.890749825 | 4.32E-11 | 1.12E-10 |
| *RYR3* | ENSG00000198838.10 | -1.888813666 | 1.97E-05 | 3.16E-05 |
| *SLC8A2* | ENSG00000118160.12 | -1.885242692 | 0.000323837 | 0.000471696 |
| *KCNK2* | ENSG00000082482.12 | -1.881029934 | 1.12E-10 | 2.79E-10 |
| *KCNQ5* | ENSG00000185760.14 | -1.880073202 | 1.50E-10 | 3.70E-10 |
| *TMEM74* | ENSG00000164841.4 | -1.879750102 | 6.65E-05 | 0.000102205 |
| *RNF112* | ENSG00000128482.14 | -1.877490754 | 2.04E-17 | 9.30E-17 |
| *KLRF1* | ENSG00000150045.10 | -1.871774263 | 6.00E-15 | 2.18E-14 |
| *FABP4* | ENSG00000170323.7 | -1.871173785 | 6.82E-13 | 2.07E-12 |
| *APOBEC3A* | ENSG00000128383.11 | -1.868874904 | 1.65E-08 | 3.41E-08 |
| *SNCB* | ENSG00000074317.9 | -1.868331234 | 0.0001192 | 0.000179718 |
| *ZG16* | ENSG00000174992.7 | -1.868063647 | 1.52E-39 | 5.92E-38 |
| *TMOD1* | ENSG00000136842.12 | -1.867397227 | 2.01E-11 | 5.37E-11 |
| *MT1M* | ENSG00000205364.3 | -1.867147602 | 2.13E-22 | 1.63E-21 |
| *KCNA5* | ENSG00000130037.4 | -1.86698171 | 4.13E-06 | 6.98E-06 |
| *ERICH3* | ENSG00000178965.12 | -1.863881511 | 3.23E-07 | 6.00E-07 |
| *DPF3* | ENSG00000205683.10 | -1.863110441 | 3.20E-26 | 3.69E-25 |
| *ENHO* | ENSG00000168913.6 | -1.856908178 | 4.99E-21 | 3.29E-20 |
| *RAB3C* | ENSG00000152932.7 | -1.854024163 | 2.24E-13 | 7.07E-13 |
| *NRAP* | ENSG00000197893.12 | -1.852195621 | 3.05E-05 | 4.82E-05 |
| *DLGAP2* | ENSG00000198010.10 | -1.851367025 | 3.41E-07 | 6.32E-07 |
| *BMP5* | ENSG00000112175.7 | -1.850445323 | 6.64E-21 | 4.31E-20 |
| *NRG3* | ENSG00000185737.11 | -1.850064947 | 3.33E-07 | 6.18E-07 |
| *SCTR* | ENSG00000080293.8 | -1.848250629 | 1.48E-09 | 3.35E-09 |
| *SEMA6D* | ENSG00000137872.14 | -1.845537704 | 2.06E-28 | 3.00E-27 |
| *ZNF385B* | ENSG00000144331.17 | -1.845487051 | 1.41E-08 | 2.94E-08 |
| *SEZ6* | ENSG00000063015.18 | -1.83966986 | 5.60E-07 | 1.02E-06 |
| *MIA2* | ENSG00000150526.10 | -1.839327345 | 0.000150283 | 0.000224481 |
| *SPEG* | ENSG00000072195.13 | -1.83885267 | 7.19E-07 | 1.30E-06 |
| *KLHL34* | ENSG00000185915.5 | -1.837855561 | 1.45E-19 | 8.14E-19 |
| *CCDC152* | ENSG00000198865.8 | -1.835473045 | 6.91E-18 | 3.28E-17 |
| *C8orf88* | ENSG00000253250.2 | -1.83497475 | 2.34E-08 | 4.78E-08 |
| *C2orf88* | ENSG00000187699.9 | -1.834488976 | 1.23E-32 | 2.66E-31 |
| *CR2* | ENSG00000117322.15 | -1.832994962 | 1.74E-08 | 3.59E-08 |
| *ATP1B2* | ENSG00000129244.7 | -1.831287046 | 2.32E-15 | 8.75E-15 |
| *OSR1* | ENSG00000143867.6 | -1.827740009 | 1.65E-12 | 4.84E-12 |
| *RSPO1* | ENSG00000169218.12 | -1.826539351 | 1.88E-14 | 6.53E-14 |
| *DPP10* | ENSG00000175497.15 | -1.82581565 | 2.22E-18 | 1.11E-17 |
| *CHRDL1* | ENSG00000101938.13 | -1.824612485 | 6.80E-21 | 4.40E-20 |
| *RUNDC3B* | ENSG00000105784.14 | -1.82338069 | 4.04E-19 | 2.16E-18 |
| *LGI3* | ENSG00000168481.7 | -1.821993573 | 4.16E-07 | 7.66E-07 |
| *NLRP7* | ENSG00000167634.11 | -1.821688081 | 1.29E-08 | 2.69E-08 |
| *KCNMA1* | ENSG00000156113.19 | -1.821585447 | 1.20E-12 | 3.56E-12 |
| *PCDH9* | ENSG00000184226.13 | -1.821345081 | 1.10E-08 | 2.31E-08 |
| *PLIN1* | ENSG00000166819.10 | -1.820785388 | 3.47E-06 | 5.90E-06 |
| *CHST8* | ENSG00000124302.11 | -1.8202829 | 2.40E-07 | 4.50E-07 |
| *FAM189A2* | ENSG00000135063.16 | -1.816118582 | 5.53E-17 | 2.43E-16 |
| *CCL14* | ENSG00000276409.3 | -1.81482071 | 1.39E-21 | 9.75E-21 |
| *TMEM255A* | ENSG00000125355.14 | -1.812168698 | 4.79E-16 | 1.94E-15 |
| *HMX2* | ENSG00000188816.3 | -1.811936388 | 6.88E-08 | 1.35E-07 |
| *TNFRSF17* | ENSG00000048462.9 | -1.811383457 | 1.91E-19 | 1.06E-18 |
| *PCDH20* | ENSG00000280165.1 | -1.809291471 | 1.00E-06 | 1.78E-06 |
| *MS4A1* | ENSG00000156738.16 | -1.807244742 | 2.79E-08 | 5.66E-08 |
| *TRIM55* | ENSG00000147573.15 | -1.804980878 | 1.66E-08 | 3.43E-08 |
| *CEND1* | ENSG00000184524.5 | -1.800543776 | 4.89E-08 | 9.74E-08 |
| *STAP1* | ENSG00000035720.6 | -1.799376002 | 2.91E-10 | 7.01E-10 |
| *ATP2B2* | ENSG00000157087.15 | -1.796811361 | 4.90E-08 | 9.75E-08 |
| *HOXD1* | ENSG00000128645.12 | -1.793562744 | 6.31E-19 | 3.31E-18 |
| *KCNJ16* | ENSG00000153822.12 | -1.792900851 | 0.002664499 | 0.003605665 |
| *PPP2R2B* | ENSG00000156475.17 | -1.792724211 | 3.07E-08 | 6.21E-08 |
| *KIF5C* | ENSG00000168280.15 | -1.7919295 | 2.24E-11 | 5.95E-11 |
| *SLC52A1* | ENSG00000132517.13 | -1.790949085 | 1.16E-07 | 2.23E-07 |
| *CYP11A1* | ENSG00000140459.16 | -1.79013083 | 9.56E-13 | 2.86E-12 |
| *LRFN5* | ENSG00000165379.12 | -1.787229928 | 7.75E-07 | 1.39E-06 |
| *SLC35F1* | ENSG00000196376.9 | -1.785920556 | 7.09E-10 | 1.65E-09 |
| *BEND5* | ENSG00000162373.11 | -1.781043158 | 2.65E-12 | 7.64E-12 |
| *GLDN* | ENSG00000186417.12 | -1.778810449 | 3.40E-06 | 5.79E-06 |
| *SCN3B* | ENSG00000166257.7 | -1.773577124 | 1.08E-11 | 2.95E-11 |
| *HAPLN1* | ENSG00000145681.9 | -1.773457354 | 1.77E-17 | 8.10E-17 |
| *HSD17B2* | ENSG00000086696.9 | -1.773036634 | 5.47E-21 | 3.58E-20 |
| *TLR10* | ENSG00000174123.9 | -1.772650061 | 3.43E-08 | 6.90E-08 |
| *ZNF835* | ENSG00000127903.13 | -1.772402543 | 6.17E-09 | 1.32E-08 |
| *PTGDR* | ENSG00000168229.3 | -1.768377345 | 3.19E-22 | 2.41E-21 |
| *PTH1R* | ENSG00000160801.12 | -1.767934303 | 5.66E-16 | 2.27E-15 |
| *SIGLEC8* | ENSG00000105366.14 | -1.766524869 | 2.19E-12 | 6.35E-12 |
| *SGCA* | ENSG00000108823.14 | -1.76445252 | 6.94E-15 | 2.51E-14 |
| *TMEM155* | ENSG00000164112.11 | -1.764287919 | 3.32E-05 | 5.23E-05 |
| *ZDHHC15* | ENSG00000102383.12 | -1.763843849 | 3.31E-09 | 7.26E-09 |
| *NKX2-2* | ENSG00000125820.5 | -1.761184174 | 4.97E-07 | 9.09E-07 |
| *PAK3* | ENSG00000077264.13 | -1.761101677 | 3.78E-11 | 9.87E-11 |
| *KCNIP1* | ENSG00000182132.11 | -1.759259979 | 2.26E-06 | 3.91E-06 |
| *CLEC3B* | ENSG00000163815.5 | -1.758334578 | 2.75E-33 | 6.36E-32 |
| *MADCAM1* | ENSG00000099866.13 | -1.755637619 | 5.09E-17 | 2.24E-16 |
| *FAM218A* | ENSG00000250486.3 | -1.755451543 | 8.02E-12 | 2.22E-11 |
| *PDE7B* | ENSG00000171408.12 | -1.75381075 | 1.31E-26 | 1.59E-25 |
| *CLDN11* | ENSG00000013297.9 | -1.753645129 | 3.99E-10 | 9.50E-10 |
| *SCUBE2* | ENSG00000175356.11 | -1.753456397 | 6.69E-22 | 4.88E-21 |
| *CCDC178* | ENSG00000166960.15 | -1.750617271 | 1.12E-09 | 2.56E-09 |
| *CD36* | ENSG00000135218.16 | -1.750134873 | 6.11E-26 | 6.83E-25 |
| *RND2* | ENSG00000108830.9 | -1.749376075 | 1.10E-06 | 1.95E-06 |
| *NPY1R* | ENSG00000164128.5 | -1.74873972 | 6.17E-16 | 2.46E-15 |
| *JPH3* | ENSG00000154118.11 | -1.747729025 | 4.14E-09 | 9.00E-09 |
| *ENTPD3* | ENSG00000168032.7 | -1.746751077 | 1.55E-16 | 6.52E-16 |
| *ABCD2* | ENSG00000173208.3 | -1.744319134 | 3.20E-13 | 9.95E-13 |
| *STXBP5L* | ENSG00000145087.11 | -1.742171682 | 0.000227913 | 0.000335348 |
| *NBEA* | ENSG00000172915.17 | -1.73998218 | 2.92E-08 | 5.91E-08 |
| *CORO2B* | ENSG00000103647.11 | -1.739396402 | 3.32E-17 | 1.49E-16 |
| *TMEM220* | ENSG00000187824.7 | -1.739297045 | 1.46E-20 | 9.08E-20 |
| *SYNM* | ENSG00000182253.13 | -1.738353617 | 9.34E-11 | 2.34E-10 |
| *WDR64* | ENSG00000162843.16 | -1.738151681 | 1.81E-06 | 3.15E-06 |
| *PRKAA2* | ENSG00000162409.9 | -1.736209914 | 2.65E-10 | 6.40E-10 |
| *MAPK10* | ENSG00000109339.17 | -1.735749855 | 9.68E-17 | 4.15E-16 |
| *SVOP* | ENSG00000166111.8 | -1.734192464 | 4.64E-11 | 1.20E-10 |
| *LY9* | ENSG00000122224.16 | -1.73405806 | 1.73E-12 | 5.07E-12 |
| *DIRAS1* | ENSG00000176490.4 | -1.731960559 | 5.62E-30 | 9.45E-29 |
| *NLGN4X* | ENSG00000146938.13 | -1.730042898 | 1.44E-13 | 4.62E-13 |
| *UTS2B* | ENSG00000188958.8 | -1.72785144 | 1.12E-06 | 1.99E-06 |
| *PTGDR2* | ENSG00000183134.4 | -1.72771153 | 2.49E-17 | 1.13E-16 |
| *SHISA3* | ENSG00000178343.4 | -1.727219631 | 7.94E-15 | 2.86E-14 |
| *CNTNAP3B* | ENSG00000154529.13 | -1.726793385 | 3.77E-05 | 5.91E-05 |
| *PDE2A* | ENSG00000186642.14 | -1.726521089 | 1.64E-14 | 5.73E-14 |
| *VIP* | ENSG00000146469.11 | -1.721449319 | 6.05E-29 | 9.23E-28 |
| *SCN4A* | ENSG00000007314.10 | -1.718095525 | 0.000170465 | 0.000253404 |
| *UGT2A3* | ENSG00000135220.9 | -1.713919707 | 2.49E-32 | 5.30E-31 |
| *HHIP* | ENSG00000164161.8 | -1.71204391 | 6.50E-17 | 2.83E-16 |
| *IL5RA* | ENSG00000091181.18 | -1.711815274 | 4.29E-10 | 1.02E-09 |
| *KCNC1* | ENSG00000129159.6 | -1.711531818 | 1.25E-07 | 2.40E-07 |
| *BVES* | ENSG00000112276.12 | -1.711509818 | 5.95E-07 | 1.08E-06 |
| *GPIHBP1* | ENSG00000277494.1 | -1.709938398 | 7.51E-09 | 1.60E-08 |
| *RBPMS2* | ENSG00000166831.7 | -1.709671599 | 1.09E-10 | 2.72E-10 |
| *MPZ* | ENSG00000158887.14 | -1.707868344 | 1.98E-15 | 7.51E-15 |
| *GNG7* | ENSG00000176533.11 | -1.707449051 | 9.27E-32 | 1.87E-30 |
| *TACR2* | ENSG00000075073.13 | -1.703512936 | 1.67E-08 | 3.45E-08 |
| *IGF1* | ENSG00000017427.14 | -1.703416859 | 4.06E-12 | 1.15E-11 |
| *BMX* | ENSG00000102010.13 | -1.703008623 | 7.08E-21 | 4.58E-20 |
| *CAMK2A* | ENSG00000070808.14 | -1.702922159 | 3.28E-08 | 6.62E-08 |
| *CARTPT* | ENSG00000164326.4 | -1.699650751 | 0.001418836 | 0.001964874 |
| *IL1RAPL1* | ENSG00000169306.8 | -1.69926855 | 1.66E-07 | 3.15E-07 |
| *FAM129C* | ENSG00000167483.16 | -1.696700189 | 0.000205593 | 0.000303638 |
| *MS4A2* | ENSG00000149534.7 | -1.696181152 | 1.82E-14 | 6.34E-14 |
| *LHFPL4* | ENSG00000156959.8 | -1.696040124 | 3.72E-11 | 9.72E-11 |
| *ADCY2* | ENSG00000078295.14 | -1.69555684 | 1.93E-05 | 3.10E-05 |
| *FILIP1* | ENSG00000118407.13 | -1.694607904 | 5.73E-07 | 1.04E-06 |
| *KIR2DL4* | ENSG00000189013.13 | -1.693052807 | 4.37E-12 | 1.23E-11 |
| *HIF3A* | ENSG00000124440.14 | -1.691748609 | 3.71E-07 | 6.86E-07 |
| *FLT3* | ENSG00000122025.13 | -1.69070103 | 3.72E-14 | 1.26E-13 |
| *NPY5R* | ENSG00000164129.10 | -1.68703753 | 1.83E-05 | 2.94E-05 |
| *SH3GL2* | ENSG00000107295.9 | -1.686316328 | 4.11E-11 | 1.07E-10 |
| *TTLL6* | ENSG00000170703.14 | -1.685015369 | 6.67E-16 | 2.65E-15 |
| *LILRA4* | ENSG00000239961.2 | -1.682959158 | 1.76E-08 | 3.63E-08 |
| *FAM163A* | ENSG00000143340.6 | -1.681587409 | 3.94E-11 | 1.03E-10 |
| *TRIM40* | ENSG00000204614.7 | -1.681415053 | 1.15E-10 | 2.86E-10 |
| *SV2B* | ENSG00000185518.10 | -1.680966016 | 9.42E-09 | 1.99E-08 |
| *KCNMB2* | ENSG00000197584.10 | -1.677502143 | 1.48E-05 | 2.40E-05 |
| *FOXP2* | ENSG00000128573.21 | -1.676930201 | 1.08E-09 | 2.47E-09 |
| *PKIB* | ENSG00000135549.13 | -1.676785647 | 1.72E-34 | 4.43E-33 |
| *ASCL1* | ENSG00000139352.3 | -1.676447991 | 1.23E-06 | 2.17E-06 |
| *PTPRT* | ENSG00000196090.11 | -1.676378322 | 9.15E-11 | 2.30E-10 |
| *SLC8A3* | ENSG00000100678.17 | -1.675715454 | 1.22E-11 | 3.31E-11 |
| *SCUBE1* | ENSG00000159307.17 | -1.673906454 | 3.77E-19 | 2.03E-18 |
| *GNG3* | ENSG00000162188.5 | -1.673748022 | 6.11E-12 | 1.71E-11 |
| *MAPT* | ENSG00000186868.14 | -1.671910613 | 1.41E-05 | 2.29E-05 |
| *CD22* | ENSG00000012124.13 | -1.671860449 | 8.34E-08 | 1.62E-07 |
| *SLC23A1* | ENSG00000170482.15 | -1.667296359 | 5.07E-11 | 1.31E-10 |
| *SCN4B* | ENSG00000177098.7 | -1.666254441 | 8.34E-24 | 7.38E-23 |
| *CYP8B1* | ENSG00000180432.5 | -1.664764662 | 3.31E-08 | 6.67E-08 |
| *FCER1A* | ENSG00000179639.9 | -1.664658846 | 5.88E-16 | 2.36E-15 |
| *MIXL1* | ENSG00000185155.10 | -1.662839262 | 3.88E-10 | 9.26E-10 |
| *CHRM4* | ENSG00000180720.7 | -1.661635208 | 1.05E-07 | 2.02E-07 |
| *MPPED2* | ENSG00000066382.15 | -1.659653533 | 3.09E-05 | 4.88E-05 |
| *RGS22* | ENSG00000132554.18 | -1.65963846 | 2.77E-07 | 5.17E-07 |
| *CD163L1* | ENSG00000177675.7 | -1.658621343 | 1.50E-17 | 6.93E-17 |
| *BHMT2* | ENSG00000132840.8 | -1.657902786 | 2.12E-08 | 4.35E-08 |
| *ASB2* | ENSG00000100628.10 | -1.656706 | 2.39E-15 | 9.01E-15 |
| *LRRC55* | ENSG00000183908.5 | -1.655851632 | 1.48E-08 | 3.07E-08 |
| *CAND2* | ENSG00000144712.10 | -1.653507304 | 4.65E-10 | 1.10E-09 |
| *AKAP6* | ENSG00000151320.9 | -1.64912375 | 2.19E-06 | 3.79E-06 |
| *NKAPL* | ENSG00000189134.3 | -1.648902671 | 2.21E-05 | 3.53E-05 |
| *TAS1R1* | ENSG00000173662.18 | -1.648516593 | 9.36E-07 | 1.67E-06 |
| *GRIN3A* | ENSG00000198785.4 | -1.648379466 | 3.74E-08 | 7.51E-08 |
| *P2RY14* | ENSG00000174944.7 | -1.646546369 | 5.10E-21 | 3.36E-20 |
| *KCNT2* | ENSG00000162687.15 | -1.643927271 | 2.71E-10 | 6.54E-10 |
| *RGS9* | ENSG00000108370.14 | -1.643766319 | 1.72E-15 | 6.56E-15 |
| *CRTAC1* | ENSG00000095713.12 | -1.642894744 | 9.58E-07 | 1.71E-06 |
| *TMEM108* | ENSG00000144868.12 | -1.642502386 | 8.87E-10 | 2.05E-09 |
| *KCNN3* | ENSG00000143603.17 | -1.642241321 | 6.31E-10 | 1.48E-09 |
| *P2RX1* | ENSG00000108405.3 | -1.641843077 | 1.40E-23 | 1.21E-22 |
| *CRABP1* | ENSG00000166426.7 | -1.64122087 | 3.64E-12 | 1.04E-11 |
| *PNOC* | ENSG00000168081.7 | -1.640655327 | 5.16E-12 | 1.45E-11 |
| *JPH2* | ENSG00000149596.6 | -1.640214802 | 1.62E-08 | 3.35E-08 |
| *LRMP* | ENSG00000118308.13 | -1.638986952 | 9.26E-16 | 3.63E-15 |
| *RPL10L* | ENSG00000165496.4 | -1.638588137 | 3.81E-19 | 2.04E-18 |
| *KCNMB1* | ENSG00000145936.7 | -1.63838542 | 5.46E-09 | 1.17E-08 |
| *LYNX1* | ENSG00000180155.17 | -1.634914117 | 2.01E-10 | 4.90E-10 |
| *KIRREL3* | ENSG00000149571.9 | -1.634516584 | 3.35E-08 | 6.75E-08 |
| *NTN1* | ENSG00000065320.7 | -1.633981191 | 3.08E-18 | 1.51E-17 |
| *PRKCB* | ENSG00000166501.11 | -1.63372689 | 4.44E-21 | 2.95E-20 |
| *ANKRD33B* | ENSG00000164236.10 | -1.633078308 | 1.46E-14 | 5.12E-14 |
| *GSTA1* | ENSG00000243955.4 | -1.630071587 | 3.08E-10 | 7.41E-10 |
| *GDNF* | ENSG00000168621.13 | -1.629741383 | 6.85E-10 | 1.60E-09 |
| *GHR* | ENSG00000112964.12 | -1.629347649 | 1.10E-13 | 3.57E-13 |
| *WDR78* | ENSG00000152763.15 | -1.626587987 | 1.02E-16 | 4.36E-16 |
| *SI* | ENSG00000090402.6 | -1.624897919 | 2.29E-13 | 7.23E-13 |
| *SYT15* | ENSG00000277758.3 | -1.623983447 | 1.52E-08 | 3.15E-08 |
| *GPM6B* | ENSG00000046653.13 | -1.622994389 | 1.00E-11 | 2.74E-11 |
| *ADAMTSL1* | ENSG00000178031.14 | -1.621670396 | 5.30E-20 | 3.12E-19 |
| *EDN3* | ENSG00000124205.14 | -1.619650412 | 1.02E-34 | 2.68E-33 |
| *OR2W3* | ENSG00000238243.3 | -1.619248772 | 4.71E-12 | 1.33E-11 |
| *SCGB2A1* | ENSG00000124939.5 | -1.619244449 | 1.49E-15 | 5.71E-15 |
| *DISP2* | ENSG00000140323.5 | -1.618989145 | 5.23E-14 | 1.75E-13 |
| *FGF10* | ENSG00000070193.4 | -1.616572716 | 4.71E-08 | 9.39E-08 |
| *ADRB2* | ENSG00000169252.5 | -1.611643508 | 1.95E-22 | 1.50E-21 |
| *CLEC10A* | ENSG00000132514.12 | -1.611168184 | 1.13E-22 | 8.93E-22 |
| *FIGN* | ENSG00000182263.12 | -1.610433672 | 2.97E-09 | 6.55E-09 |
| *ACADL* | ENSG00000115361.6 | -1.606369663 | 3.02E-05 | 4.77E-05 |
| *ATP8A2* | ENSG00000132932.15 | -1.605173163 | 9.78E-10 | 2.25E-09 |
| *PGM5* | ENSG00000154330.11 | -1.599895495 | 9.66E-14 | 3.15E-13 |
| *SYT5* | ENSG00000129990.13 | -1.599779282 | 3.05E-08 | 6.17E-08 |
| *UCN3* | ENSG00000178473.6 | -1.599112356 | 1.74E-16 | 7.29E-16 |
| *CHST5* | ENSG00000135702.13 | -1.596956372 | 5.42E-17 | 2.38E-16 |
| *ATP6V0D2* | ENSG00000147614.3 | -1.595678971 | 1.65E-12 | 4.84E-12 |
| *FMO2* | ENSG00000094963.12 | -1.595189883 | 9.70E-06 | 1.59E-05 |
| *HRASLS2* | ENSG00000133328.3 | -1.592815742 | 7.06E-16 | 2.80E-15 |
| *EDN2* | ENSG00000127129.8 | -1.591059987 | 1.10E-08 | 2.31E-08 |
| *TMEM132D* | ENSG00000151952.13 | -1.589736108 | 1.48E-09 | 3.35E-09 |
| *HIPK4* | ENSG00000160396.8 | -1.589111239 | 0.000168535 | 0.000250679 |
| *HLF* | ENSG00000108924.12 | -1.58461156 | 1.00E-07 | 1.93E-07 |
| *CLECL1* | ENSG00000184293.6 | -1.583115824 | 7.60E-12 | 2.11E-11 |
| *SYNPO2* | ENSG00000172403.9 | -1.581667353 | 1.11E-12 | 3.31E-12 |
| *PSD* | ENSG00000059915.15 | -1.579134472 | 7.39E-08 | 1.45E-07 |
| *UGT2B15* | ENSG00000196620.7 | -1.578578875 | 2.82E-10 | 6.80E-10 |
| *SLCO1C1* | ENSG00000139155.7 | -1.577873414 | 6.61E-11 | 1.68E-10 |
| *MEGF10* | ENSG00000145794.15 | -1.576857091 | 3.59E-09 | 7.84E-09 |
| *HS6ST3* | ENSG00000185352.8 | -1.576472972 | 2.35E-05 | 3.74E-05 |
| *CDK15* | ENSG00000138395.13 | -1.576376299 | 7.02E-10 | 1.64E-09 |
| *EPB41L3* | ENSG00000082397.14 | -1.56993279 | 1.99E-49 | 1.43E-47 |
| *CRYBA2* | ENSG00000163499.10 | -1.569218784 | 7.69E-10 | 1.79E-09 |
| *DHRS9* | ENSG00000073737.15 | -1.569008587 | 2.80E-29 | 4.44E-28 |
| *CSMD1* | ENSG00000183117.16 | -1.567077062 | 3.70E-08 | 7.43E-08 |
| *LRCH2* | ENSG00000130224.13 | -1.566759794 | 3.02E-07 | 5.62E-07 |
| *TPPP2* | ENSG00000179636.13 | -1.566691503 | 4.20E-09 | 9.12E-09 |
| *SLIT3* | ENSG00000184347.13 | -1.565780343 | 7.17E-17 | 3.11E-16 |
| *MAP6* | ENSG00000171533.10 | -1.565483158 | 3.64E-07 | 6.73E-07 |
| *GCNT4* | ENSG00000176928.5 | -1.564805769 | 1.67E-10 | 4.10E-10 |
| *RGMA* | ENSG00000182175.12 | -1.564113041 | 1.72E-12 | 5.04E-12 |
| *FAM189A1* | ENSG00000104059.4 | -1.56403304 | 1.98E-29 | 3.16E-28 |
| *GPR27* | ENSG00000170837.2 | -1.563809402 | 3.86E-14 | 1.31E-13 |
| *C6* | ENSG00000039537.12 | -1.563757584 | 2.33E-07 | 4.38E-07 |
| *TENM1* | ENSG00000009694.12 | -1.562693236 | 0.00094897 | 0.001334009 |
| *JAM2* | ENSG00000154721.13 | -1.558736456 | 5.89E-24 | 5.29E-23 |
| *BTNL8* | ENSG00000113303.10 | -1.558375369 | 5.27E-24 | 4.78E-23 |
| *LRRC7* | ENSG00000033122.17 | -1.556786962 | 1.76E-12 | 5.15E-12 |
| *CCDC13* | ENSG00000244607.4 | -1.556550884 | 8.89E-09 | 1.88E-08 |
| *CHRNA3* | ENSG00000080644.14 | -1.555068435 | 4.81E-13 | 1.47E-12 |
| *SMIM2* | ENSG00000139656.6 | -1.55431499 | 4.02E-09 | 8.75E-09 |
| *NUDT10* | ENSG00000122824.9 | -1.552837659 | 3.25E-07 | 6.04E-07 |
| *NAALADL1* | ENSG00000168060.13 | -1.551951195 | 8.33E-15 | 2.99E-14 |
| *MAP1LC3C* | ENSG00000197769.5 | -1.551893472 | 1.35E-06 | 2.38E-06 |
| *SALL2* | ENSG00000165821.10 | -1.55179935 | 2.50E-10 | 6.05E-10 |
| *SKIDA1* | ENSG00000180592.15 | -1.549350721 | 0.001100742 | 0.001538552 |
| *TDRD10* | ENSG00000163239.11 | -1.547830239 | 3.97E-14 | 1.34E-13 |
| *HRASLS5* | ENSG00000168004.8 | -1.547382877 | 4.74E-05 | 7.37E-05 |
| *IRF4* | ENSG00000137265.13 | -1.545891469 | 2.47E-14 | 8.50E-14 |
| *TPO* | ENSG00000115705.19 | -1.54578068 | 4.86E-06 | 8.16E-06 |
| *THRB* | ENSG00000151090.16 | -1.545167252 | 7.77E-19 | 4.04E-18 |
| *MT1H* | ENSG00000205358.3 | -1.545060656 | 9.80E-17 | 4.20E-16 |
| *CDKL2* | ENSG00000138769.9 | -1.54374235 | 2.37E-09 | 5.28E-09 |
| *LRRC4C* | ENSG00000148948.6 | -1.543013379 | 7.82E-06 | 1.29E-05 |
| *GALNT15* | ENSG00000131386.16 | -1.542249693 | 7.25E-10 | 1.69E-09 |
| *CELA3A* | ENSG00000142789.18 | -1.541561914 | 0.001492248 | 0.002061739 |
| *KCNK10* | ENSG00000100433.14 | -1.540046117 | 9.74E-17 | 4.18E-16 |
| *CCR2* | ENSG00000121807.5 | -1.537618545 | 1.08E-15 | 4.20E-15 |
| *SCIN* | ENSG00000006747.13 | -1.537183742 | 1.79E-21 | 1.24E-20 |
| *PLA2G5* | ENSG00000127472.9 | -1.533823691 | 5.44E-06 | 9.10E-06 |
| *CLEC4F* | ENSG00000152672.6 | -1.532975417 | 5.40E-11 | 1.39E-10 |
| *FCRL3* | ENSG00000160856.19 | -1.529944225 | 7.46E-06 | 1.24E-05 |
| *THRSP* | ENSG00000151365.2 | -1.528260085 | 0.026708688 | 0.032990701 |
| *PRRG3* | ENSG00000130032.14 | -1.526471484 | 5.74E-07 | 1.04E-06 |
| *CACNG7* | ENSG00000105605.6 | -1.525963469 | 8.12E-06 | 1.34E-05 |
| *PCSK5* | ENSG00000099139.12 | -1.521402211 | 1.68E-26 | 2.01E-25 |
| *SUSD5* | ENSG00000173705.7 | -1.520488666 | 3.27E-06 | 5.58E-06 |
| *DPT* | ENSG00000143196.4 | -1.519486575 | 1.20E-20 | 7.57E-20 |
| *CCR9* | ENSG00000173585.14 | -1.519145425 | 6.94E-07 | 1.25E-06 |
| *VIPR2* | ENSG00000106018.12 | -1.518624715 | 1.43E-05 | 2.32E-05 |
| *COL21A1* | ENSG00000124749.15 | -1.517147673 | 3.36E-05 | 5.29E-05 |
| *BTLA* | ENSG00000186265.8 | -1.515647669 | 6.05E-11 | 1.54E-10 |
| *CCNA1* | ENSG00000133101.8 | -1.512363645 | 0.000388089 | 0.000562266 |
| *KCNB2* | ENSG00000182674.5 | -1.512218039 | 2.55E-09 | 5.66E-09 |
| *SLC51B* | ENSG00000186198.3 | -1.512126558 | 1.63E-34 | 4.20E-33 |
| *KCNIP4* | ENSG00000185774.13 | -1.510012943 | 6.26E-09 | 1.34E-08 |
| *REEP2* | ENSG00000132563.14 | -1.507621931 | 8.49E-09 | 1.80E-08 |
| *UBE2QL1* | ENSG00000215218.3 | -1.507444199 | 1.74E-10 | 4.27E-10 |
| *SYNGR1* | ENSG00000100321.13 | -1.504152039 | 3.20E-13 | 9.95E-13 |
| *ADTRP* | ENSG00000111863.11 | -1.504112752 | 3.95E-33 | 8.97E-32 |
| *CDO1* | ENSG00000129596.4 | -1.503929211 | 6.79E-08 | 1.33E-07 |
| *HMCN2* | ENSG00000148357.15 | -1.50343737 | 3.35E-14 | 1.14E-13 |
| *MYH15* | ENSG00000144821.8 | -1.502467408 | 1.44E-14 | 5.05E-14 |
| *NGFR* | ENSG00000064300.7 | -1.500716462 | 7.17E-15 | 2.59E-14 |
| *NACAD* | ENSG00000136274.8 | -1.499940206 | 2.22E-09 | 4.95E-09 |
| *FABP2* | ENSG00000145384.3 | -1.499106667 | 3.72E-23 | 3.08E-22 |
| *B3GALT5* | ENSG00000183778.16 | -1.497761992 | 7.00E-18 | 3.32E-17 |
| *MFAP5* | ENSG00000197614.9 | -1.497303486 | 1.21E-18 | 6.16E-18 |
| *ADAM33* | ENSG00000149451.16 | -1.497070188 | 2.16E-12 | 6.27E-12 |
| *RDH5* | ENSG00000135437.8 | -1.496580688 | 5.03E-31 | 9.42E-30 |
| *LGALS12* | ENSG00000133317.13 | -1.496375016 | 0.00081136 | 0.001147553 |
| *PRDM6* | ENSG00000061455.10 | -1.494942912 | 1.96E-05 | 3.14E-05 |
| *SYN2* | ENSG00000157152.15 | -1.494688692 | 2.53E-07 | 4.74E-07 |
| *SETBP1* | ENSG00000152217.15 | -1.491515803 | 4.78E-14 | 1.60E-13 |
| *CCL8* | ENSG00000108700.4 | -1.490135722 | 3.45E-14 | 1.17E-13 |
| *MSTN* | ENSG00000138379.4 | -1.488655985 | 0.000179551 | 0.000266373 |
| *NEFH* | ENSG00000100285.9 | -1.487415368 | 3.96E-09 | 8.62E-09 |
| *SH2D1B* | ENSG00000198574.5 | -1.487359668 | 2.48E-11 | 6.57E-11 |
| *SYPL2* | ENSG00000143028.8 | -1.48682631 | 5.02E-05 | 7.79E-05 |
| *ZNF471* | ENSG00000196263.6 | -1.486691021 | 1.33E-13 | 4.28E-13 |
| *DLG2* | ENSG00000150672.15 | -1.486482554 | 2.42E-06 | 4.17E-06 |
| *POPDC2* | ENSG00000121577.12 | -1.484514036 | 1.98E-06 | 3.44E-06 |
| *ADRA1B* | ENSG00000170214.3 | -1.481050842 | 5.41E-12 | 1.52E-11 |
| *EFCAB1* | ENSG00000034239.9 | -1.480325709 | 7.71E-11 | 1.95E-10 |
| *TUB* | ENSG00000166402.7 | -1.479233122 | 5.85E-11 | 1.50E-10 |
| *FDCSP* | ENSG00000181617.5 | -1.478075 | 2.79E-08 | 5.66E-08 |
| *DDX25* | ENSG00000109832.11 | -1.477712993 | 2.02E-05 | 3.23E-05 |
| *KCNAB1* | ENSG00000169282.16 | -1.476829334 | 5.88E-08 | 1.16E-07 |
| *KNG1* | ENSG00000113889.10 | -1.469709472 | 3.61E-07 | 6.68E-07 |
| *CCR10* | ENSG00000184451.5 | -1.465171993 | 1.78E-10 | 4.36E-10 |
| *EXTL1* | ENSG00000158008.8 | -1.461908037 | 5.92E-07 | 1.08E-06 |
| *GAPT* | ENSG00000175857.7 | -1.461880933 | 1.74E-11 | 4.67E-11 |
| *CYP2C8* | ENSG00000138115.12 | -1.460287505 | 0.001483832 | 0.002051027 |
| *P2RY1* | ENSG00000169860.6 | -1.460116631 | 2.58E-40 | 1.06E-38 |
| *KCNG1* | ENSG00000026559.12 | -1.45936404 | 5.50E-12 | 1.54E-11 |
| *RBM24* | ENSG00000112183.13 | -1.458429387 | 4.47E-09 | 9.68E-09 |
| *CALB2* | ENSG00000172137.17 | -1.457901153 | 7.42E-21 | 4.79E-20 |
| *RAB9B* | ENSG00000123570.3 | -1.457410932 | 4.59E-07 | 8.42E-07 |
| *SYT6* | ENSG00000134207.13 | -1.453382819 | 0.004052282 | 0.00540281 |
| *NPAS3* | ENSG00000151322.17 | -1.450472838 | 1.56E-07 | 2.97E-07 |
| *AOX1* | ENSG00000138356.12 | -1.450124105 | 2.67E-13 | 8.39E-13 |
| *GUCA2A* | ENSG00000197273.3 | -1.447694981 | 6.02E-44 | 3.13E-42 |
| *HDAC9* | ENSG00000048052.20 | -1.447182978 | 2.72E-24 | 2.54E-23 |
| *VAT1L* | ENSG00000171724.2 | -1.44705986 | 8.64E-21 | 5.54E-20 |
| *SLC6A15* | ENSG00000072041.15 | -1.446264571 | 3.09E-06 | 5.28E-06 |
| *ADORA3* | ENSG00000121933.16 | -1.443557799 | 7.32E-14 | 2.41E-13 |
| *SCG2* | ENSG00000171951.4 | -1.442583078 | 4.89E-22 | 3.62E-21 |
| *SYNC* | ENSG00000162520.13 | -1.442526414 | 5.29E-20 | 3.11E-19 |
| *PTX3* | ENSG00000163661.3 | -1.4422709 | 1.85E-13 | 5.88E-13 |
| *C2orf74* | ENSG00000237651.5 | -1.441526437 | 1.69E-09 | 3.81E-09 |
| *MICU3* | ENSG00000155970.10 | -1.440917218 | 7.86E-08 | 1.53E-07 |
| *OPN4* | ENSG00000122375.10 | -1.440524859 | 6.43E-05 | 9.89E-05 |
| *SLC9A9* | ENSG00000181804.13 | -1.439763654 | 1.94E-26 | 2.30E-25 |
| *PACSIN1* | ENSG00000124507.9 | -1.439539439 | 1.27E-12 | 3.76E-12 |
| *MPP2* | ENSG00000108852.13 | -1.43878309 | 1.82E-08 | 3.75E-08 |
| *TCF21* | ENSG00000118526.6 | -1.438204207 | 3.41E-28 | 4.82E-27 |
| *AQP7* | ENSG00000165269.11 | -1.43780869 | 3.77E-14 | 1.28E-13 |
| *PTN* | ENSG00000105894.10 | -1.437368641 | 7.77E-24 | 6.90E-23 |
| *CLCA2* | ENSG00000137975.7 | -1.436973922 | 4.12E-11 | 1.07E-10 |
| *NAP1L3* | ENSG00000186310.9 | -1.436652016 | 3.72E-11 | 9.72E-11 |
| *RORB* | ENSG00000198963.9 | -1.436530598 | 1.48E-07 | 2.82E-07 |
| *SYT15* | ENSG00000204176.12 | -1.432780862 | 2.84E-07 | 5.30E-07 |
| *NRG4* | ENSG00000169752.15 | -1.431493863 | 3.68E-10 | 8.80E-10 |
| *DYNC1I1* | ENSG00000158560.13 | -1.431091926 | 3.18E-05 | 5.02E-05 |
| *SFTPD* | ENSG00000133661.14 | -1.429118052 | 1.43E-06 | 2.51E-06 |
| *OTUD7A* | ENSG00000169918.8 | -1.428958312 | 3.62E-16 | 1.48E-15 |
| *BFSP2* | ENSG00000170819.4 | -1.427955291 | 2.59E-05 | 4.11E-05 |
| *NECAB2* | ENSG00000103154.8 | -1.427097361 | 4.68E-06 | 7.88E-06 |
| *TMEM132B* | ENSG00000139364.9 | -1.424261587 | 1.90E-06 | 3.30E-06 |
| *FAM216B* | ENSG00000179813.5 | -1.42055161 | 1.75E-07 | 3.32E-07 |
| *LGI4* | ENSG00000153902.12 | -1.417769456 | 1.83E-18 | 9.20E-18 |
| *GPER1* | ENSG00000164850.13 | -1.417351879 | 1.71E-17 | 7.83E-17 |
| *FAM129A* | ENSG00000135842.15 | -1.413625308 | 1.01E-14 | 3.60E-14 |
| *CHP2* | ENSG00000166869.2 | -1.41361869 | 1.30E-36 | 3.98E-35 |
| *CDKL1* | ENSG00000100490.8 | -1.411237854 | 3.30E-22 | 2.48E-21 |
| *RAB39B* | ENSG00000155961.4 | -1.410943784 | 4.34E-15 | 1.60E-14 |
| *VPREB3* | ENSG00000128218.7 | -1.410118167 | 2.06E-10 | 5.02E-10 |
| *HSPB6* | ENSG00000004776.10 | -1.409644621 | 3.06E-15 | 1.14E-14 |
| *FAM13C* | ENSG00000148541.11 | -1.409259548 | 2.71E-09 | 6.00E-09 |
| *LRRN3* | ENSG00000173114.11 | -1.408252448 | 1.75E-13 | 5.57E-13 |
| *TLR7* | ENSG00000196664.4 | -1.407091016 | 7.58E-16 | 2.99E-15 |
| *CD209* | ENSG00000090659.16 | -1.405081491 | 3.82E-22 | 2.86E-21 |
| *GPR17* | ENSG00000144230.15 | -1.403883067 | 5.51E-07 | 1.00E-06 |
| *PLAC9* | ENSG00000189129.12 | -1.399600921 | 2.94E-26 | 3.39E-25 |
| *HSPB7* | ENSG00000173641.16 | -1.398608191 | 1.03E-07 | 1.99E-07 |
| *CIDEB* | ENSG00000136305.10 | -1.396329864 | 1.07E-14 | 3.81E-14 |
| *BMP6* | ENSG00000153162.8 | -1.396142952 | 1.30E-17 | 6.03E-17 |
| *CA14* | ENSG00000118298.9 | -1.39471484 | 8.98E-05 | 0.000136711 |
| *PLCL2* | ENSG00000154822.14 | -1.392950738 | 3.25E-27 | 4.18E-26 |
| *CFAP54* | ENSG00000188596.8 | -1.392251849 | 4.38E-13 | 1.35E-12 |
| *ADH1C* | ENSG00000248144.4 | -1.389767612 | 9.00E-44 | 4.64E-42 |
| *SHE* | ENSG00000169291.8 | -1.388499341 | 3.67E-20 | 2.19E-19 |
| *NKX2-3* | ENSG00000119919.10 | -1.387261509 | 1.18E-26 | 1.44E-25 |
| *CYSLTR1* | ENSG00000173198.5 | -1.386412401 | 4.04E-20 | 2.40E-19 |
| *GPC5* | ENSG00000179399.12 | -1.385823641 | 9.13E-06 | 1.50E-05 |
| *CPNE4* | ENSG00000196353.10 | -1.384788258 | 2.38E-07 | 4.47E-07 |
| *CPAMD8* | ENSG00000160111.11 | -1.3841136 | 2.23E-14 | 7.70E-14 |
| *FAM43B* | ENSG00000183114.7 | -1.384050903 | 5.22E-08 | 1.04E-07 |
| *KLK15* | ENSG00000174562.12 | -1.383618199 | 4.09E-09 | 8.89E-09 |
| *SLC38A4* | ENSG00000139209.14 | -1.381670462 | 2.46E-10 | 5.96E-10 |
| *EPB41L4A* | ENSG00000129595.11 | -1.381651026 | 8.03E-16 | 3.16E-15 |
| *PLN* | ENSG00000198523.5 | -1.380727912 | 3.98E-11 | 1.04E-10 |
| *KL* | ENSG00000133116.7 | -1.378926397 | 5.91E-11 | 1.51E-10 |
| *TRIM50* | ENSG00000146755.9 | -1.377230722 | 1.76E-05 | 2.83E-05 |
| *ITGA8* | ENSG00000077943.7 | -1.376148129 | 3.82E-23 | 3.16E-22 |
| *AFF2* | ENSG00000155966.12 | -1.375425816 | 0.011070086 | 0.01416949 |
| *CLIP4* | ENSG00000115295.18 | -1.373617958 | 1.70E-12 | 4.98E-12 |
| *CCL13* | ENSG00000181374.6 | -1.369974256 | 2.67E-16 | 1.10E-15 |
| *UGT2B17* | ENSG00000197888.2 | -1.369887469 | 5.40E-10 | 1.27E-09 |
| *PDE1C* | ENSG00000154678.15 | -1.369380936 | 1.24E-16 | 5.26E-16 |
| *GIMAP5* | ENSG00000196329.9 | -1.367813913 | 3.93E-13 | 1.22E-12 |
| *SMPX* | ENSG00000091482.5 | -1.366680636 | 0.000611404 | 0.000872242 |
| *CCDC85A* | ENSG00000055813.5 | -1.366513384 | 6.71E-07 | 1.21E-06 |
| *LANCL3* | ENSG00000147036.10 | -1.364947295 | 3.96E-16 | 1.61E-15 |
| *B3GNT6* | ENSG00000198488.9 | -1.363810917 | 8.82E-30 | 1.45E-28 |
| *C14orf132* | ENSG00000227051.5 | -1.359973157 | 8.57E-15 | 3.07E-14 |
| *CR1* | ENSG00000203710.9 | -1.359673835 | 1.61E-06 | 2.81E-06 |
| *DACT3* | ENSG00000197380.9 | -1.359487728 | 6.84E-09 | 1.46E-08 |
| *AKR1C2* | ENSG00000151632.15 | -1.359487548 | 1.44E-19 | 8.10E-19 |
| *PRELP* | ENSG00000188783.5 | -1.358490934 | 1.40E-14 | 4.92E-14 |
| *PAX5* | ENSG00000196092.11 | -1.356703651 | 8.08E-05 | 0.000123447 |
| *SSBP2* | ENSG00000145687.14 | -1.353242437 | 6.97E-19 | 3.64E-18 |
| *ZIK1* | ENSG00000171649.10 | -1.352960949 | 9.13E-13 | 2.74E-12 |
| *SNCA* | ENSG00000145335.14 | -1.351030264 | 4.81E-08 | 9.58E-08 |
| *MB* | ENSG00000198125.11 | -1.34981766 | 2.66E-18 | 1.31E-17 |
| *EML5* | ENSG00000165521.14 | -1.349566372 | 4.16E-07 | 7.66E-07 |
| *MAP2* | ENSG00000078018.18 | -1.348009813 | 4.67E-08 | 9.31E-08 |
| *ZNF492* | ENSG00000229676.2 | -1.347867544 | 1.10E-08 | 2.31E-08 |
| *VWA3B* | ENSG00000168658.17 | -1.346077266 | 3.53E-14 | 1.20E-13 |
| *CYS1* | ENSG00000205795.4 | -1.346051837 | 1.17E-14 | 4.14E-14 |
| *ZNF626* | ENSG00000188171.13 | -1.344999428 | 2.95E-10 | 7.10E-10 |
| *MYH11* | ENSG00000133392.15 | -1.344910563 | 3.50E-16 | 1.43E-15 |
| *HSPB8* | ENSG00000152137.5 | -1.344774234 | 1.32E-10 | 3.27E-10 |
| *ZNF677* | ENSG00000197928.9 | -1.343777245 | 4.08E-10 | 9.71E-10 |
| *KLHL10* | ENSG00000161594.6 | -1.343146771 | 0.002887615 | 0.003897701 |
| *SLC26A2* | ENSG00000155850.7 | -1.343128238 | 1.17E-24 | 1.14E-23 |
| *GPR82* | ENSG00000171657.5 | -1.342653326 | 2.41E-10 | 5.84E-10 |
| *CPNE8* | ENSG00000139117.12 | -1.341802899 | 1.88E-26 | 2.23E-25 |
| *SLIT2* | ENSG00000145147.18 | -1.341550189 | 5.75E-11 | 1.47E-10 |
| *AR* | ENSG00000169083.14 | -1.341017243 | 1.43E-05 | 2.32E-05 |
| *ANKRD35* | ENSG00000198483.11 | -1.34048801 | 8.08E-11 | 2.04E-10 |
| *TXLNB* | ENSG00000164440.13 | -1.338593618 | 2.33E-09 | 5.19E-09 |
| *LRRTM4* | ENSG00000176204.12 | -1.338083328 | 4.08E-06 | 6.90E-06 |
| *F13A1* | ENSG00000124491.14 | -1.337701217 | 4.59E-19 | 2.44E-18 |
| *L1CAM* | ENSG00000198910.11 | -1.336933115 | 9.10E-14 | 2.97E-13 |
| *SYP* | ENSG00000102003.9 | -1.335335983 | 1.47E-20 | 9.13E-20 |
| *TRIM58* | ENSG00000162722.8 | -1.334775715 | 4.97E-13 | 1.52E-12 |
| *CIDEC* | ENSG00000187288.9 | -1.334627498 | 8.31E-11 | 2.10E-10 |
| *SLC26A3* | ENSG00000091138.11 | -1.33357787 | 5.44E-46 | 3.33E-44 |
| *LRRC18* | ENSG00000165383.9 | -1.333299922 | 1.67E-07 | 3.17E-07 |
| *SUGCT* | ENSG00000175600.14 | -1.331574571 | 1.43E-19 | 8.05E-19 |
| *HPCAL4* | ENSG00000116983.11 | -1.331063655 | 0.000423457 | 0.000611673 |
| *IL6R* | ENSG00000160712.11 | -1.331040358 | 4.96E-41 | 2.15E-39 |
| *LRP1B* | ENSG00000168702.15 | -1.330592324 | 0.021779589 | 0.027160387 |
| *ST8SIA1* | ENSG00000111728.9 | -1.329405018 | 1.31E-08 | 2.73E-08 |
| *GPT* | ENSG00000167701.12 | -1.327772961 | 2.29E-33 | 5.33E-32 |
| *FBXL22* | ENSG00000197361.6 | -1.3264273 | 2.40E-06 | 4.14E-06 |
| *CD19* | ENSG00000177455.10 | -1.324480612 | 3.17E-07 | 5.89E-07 |
| *STK32B* | ENSG00000152953.11 | -1.324304708 | 3.98E-12 | 1.13E-11 |
| *PIK3CG* | ENSG00000105851.9 | -1.32240292 | 7.33E-16 | 2.90E-15 |
| *SLC2A4* | ENSG00000181856.13 | -1.321607503 | 1.14E-08 | 2.39E-08 |
| *GPR18* | ENSG00000125245.11 | -1.321498143 | 2.78E-09 | 6.14E-09 |
| *NEU4* | ENSG00000204099.10 | -1.318992488 | 2.62E-22 | 1.99E-21 |
| *PTPRN* | ENSG00000054356.12 | -1.318966933 | 3.62E-07 | 6.70E-07 |
| *STAC2* | ENSG00000141750.6 | -1.317101673 | 6.79E-06 | 1.13E-05 |
| *HEPACAM2* | ENSG00000188175.8 | -1.313779552 | 2.49E-27 | 3.25E-26 |
| *ZNF135* | ENSG00000176293.18 | -1.313428625 | 8.33E-10 | 1.93E-09 |
| *RERG* | ENSG00000134533.5 | -1.310374292 | 6.30E-09 | 1.35E-08 |
| *EEF1A2* | ENSG00000101210.9 | -1.308099945 | 1.73E-11 | 4.65E-11 |
| *FAM151A* | ENSG00000162391.10 | -1.307954103 | 4.38E-10 | 1.04E-09 |
| *GPR31* | ENSG00000120436.3 | -1.307446651 | 4.08E-05 | 6.38E-05 |
| *PABPC5* | ENSG00000174740.7 | -1.306067566 | 9.15E-06 | 1.50E-05 |
| *OLFM1* | ENSG00000130558.17 | -1.303232473 | 1.89E-19 | 1.05E-18 |
| *COLEC12* | ENSG00000158270.11 | -1.302789792 | 9.55E-22 | 6.86E-21 |
| *CFP* | ENSG00000126759.11 | -1.300971584 | 2.77E-13 | 8.68E-13 |
| *GP2* | ENSG00000169347.15 | -1.300741887 | 1.84E-11 | 4.93E-11 |
| *PHLPP2* | ENSG00000040199.17 | -1.300591206 | 1.93E-18 | 9.69E-18 |
| *ANKRD53* | ENSG00000144031.10 | -1.300537349 | 3.69E-09 | 8.05E-09 |
| *DSEL* | ENSG00000171451.13 | -1.300032481 | 2.12E-15 | 8.02E-15 |
| *YPEL4* | ENSG00000166793.9 | -1.298311337 | 5.81E-10 | 1.36E-09 |
| *SLC13A2* | ENSG00000007216.13 | -1.297593137 | 1.44E-12 | 4.24E-12 |
| *FCRL5* | ENSG00000143297.17 | -1.296757909 | 3.31E-10 | 7.94E-10 |
| *RBMS3* | ENSG00000144642.19 | -1.29396177 | 8.50E-11 | 2.14E-10 |
| *CDKN2B* | ENSG00000147883.10 | -1.293246458 | 2.63E-26 | 3.06E-25 |
| *CHRNA1* | ENSG00000138435.13 | -1.290558579 | 5.57E-10 | 1.31E-09 |
| *MMP28* | ENSG00000271447.4 | -1.289917603 | 4.55E-32 | 9.47E-31 |
| *PPP2R3A* | ENSG00000073711.9 | -1.289056897 | 2.83E-41 | 1.24E-39 |
| *TMTC1* | ENSG00000133687.14 | -1.288849678 | 8.83E-11 | 2.22E-10 |
| *RPRM* | ENSG00000177519.3 | -1.28857558 | 0.002494528 | 0.003386322 |
| *OTOA* | ENSG00000155719.15 | -1.285926475 | 8.62E-08 | 1.68E-07 |
| *TTLL7* | ENSG00000137941.15 | -1.282975811 | 8.43E-11 | 2.13E-10 |
| *ZNF781* | ENSG00000196381.9 | -1.281505952 | 4.02E-07 | 7.41E-07 |
| *RADIL* | ENSG00000157927.15 | -1.280709635 | 1.37E-09 | 3.11E-09 |
| *SOX5* | ENSG00000134532.14 | -1.280704468 | 1.72E-10 | 4.22E-10 |
| *MYBPC2* | ENSG00000086967.9 | -1.278816142 | 1.59E-05 | 2.57E-05 |
| *CPED1* | ENSG00000106034.16 | -1.277511453 | 2.40E-12 | 6.93E-12 |
| *FAXC* | ENSG00000146267.11 | -1.276765712 | 0.001098115 | 0.001535019 |
| *TEPP* | ENSG00000159648.10 | -1.27621073 | 5.30E-05 | 8.21E-05 |
| *LYPD8* | ENSG00000259823.4 | -1.275875741 | 2.09E-26 | 2.46E-25 |
| *SOGA3* | ENSG00000255330.7 | -1.273499558 | 7.31E-07 | 1.32E-06 |
| *SRPX* | ENSG00000101955.13 | -1.272274554 | 1.89E-19 | 1.05E-18 |
| *CERS1* | ENSG00000223802.6 | -1.270740137 | 7.47E-07 | 1.35E-06 |
| *CD200R1* | ENSG00000163606.9 | -1.270671462 | 2.34E-11 | 6.21E-11 |
| *KCNH1* | ENSG00000143473.10 | -1.270389623 | 3.91E-10 | 9.32E-10 |
| *NRG1* | ENSG00000157168.17 | -1.269699985 | 2.30E-11 | 6.11E-11 |
| *SRL* | ENSG00000185739.12 | -1.269656163 | 2.70E-08 | 5.49E-08 |
| *PDK4* | ENSG00000004799.7 | -1.267468058 | 2.20E-23 | 1.86E-22 |
| *FRMD3* | ENSG00000172159.14 | -1.263662441 | 3.51E-22 | 2.64E-21 |
| *AK5* | ENSG00000154027.17 | -1.263498472 | 2.88E-08 | 5.84E-08 |
| *ZNF157* | ENSG00000147117.7 | -1.262900285 | 3.63E-06 | 6.16E-06 |
| *NR5A2* | ENSG00000116833.12 | -1.262680845 | 2.80E-28 | 4.00E-27 |
| *ADAMTS8* | ENSG00000134917.9 | -1.260037657 | 1.67E-05 | 2.69E-05 |
| *CAMP* | ENSG00000164047.4 | -1.258446922 | 5.55E-05 | 8.58E-05 |
| *WFIKKN2* | ENSG00000173714.7 | -1.258269669 | 0.000672666 | 0.000956727 |
| *BRINP2* | ENSG00000198797.6 | -1.256441672 | 0.000799133 | 0.001131191 |
| *FHL1* | ENSG00000022267.15 | -1.254707583 | 1.22E-18 | 6.21E-18 |
| *GYPE* | ENSG00000197465.12 | -1.25445815 | 1.22E-09 | 2.78E-09 |
| *TESPA1* | ENSG00000135426.13 | -1.2537912 | 2.88E-13 | 9.01E-13 |
| *GPR37L1* | ENSG00000170075.8 | -1.252600795 | 1.91E-10 | 4.67E-10 |
| *CHRNB4* | ENSG00000117971.10 | -1.251485675 | 0.000156317 | 0.000233179 |
| *CPM* | ENSG00000135678.10 | -1.250135034 | 1.18E-25 | 1.27E-24 |
| *CHRM5* | ENSG00000184984.9 | -1.249951418 | 1.57E-08 | 3.25E-08 |
| *CAMK4* | ENSG00000152495.9 | -1.249785754 | 1.07E-18 | 5.48E-18 |
| *ALAS2* | ENSG00000158578.17 | -1.248436232 | 0.004314112 | 0.005739532 |
| *ADD2* | ENSG00000075340.21 | -1.24704875 | 3.02E-06 | 5.16E-06 |
| *SEMA6A* | ENSG00000092421.15 | -1.243678099 | 1.08E-38 | 3.93E-37 |
| *LMOD1* | ENSG00000163431.12 | -1.243218251 | 2.89E-15 | 1.08E-14 |
| *NPPC* | ENSG00000163273.3 | -1.242987844 | 0.000180471 | 0.000267635 |
| *FGF9* | ENSG00000102678.6 | -1.241927672 | 4.89E-09 | 1.06E-08 |
| *ANPEP* | ENSG00000166825.12 | -1.239774578 | 1.03E-21 | 7.36E-21 |
| *CCL16* | ENSG00000275152.3 | -1.239423865 | 4.23E-07 | 7.78E-07 |
| *POU2AF1* | ENSG00000110777.10 | -1.236641091 | 7.71E-14 | 2.53E-13 |
| *TMEM156* | ENSG00000121895.7 | -1.236136053 | 9.93E-10 | 2.28E-09 |
| *MOGAT2* | ENSG00000166391.13 | -1.234201785 | 2.63E-33 | 6.11E-32 |
| *SEMA3G* | ENSG00000010319.5 | -1.232981817 | 6.54E-17 | 2.85E-16 |
| *KIR3DL2* | ENSG00000240403.4 | -1.232428402 | 0.00052354 | 0.000750632 |
| *MYO3A* | ENSG00000095777.13 | -1.232142643 | 5.34E-05 | 8.26E-05 |
| *RTN1* | ENSG00000139970.15 | -1.231350634 | 2.06E-13 | 6.53E-13 |
| *DTNA* | ENSG00000134769.20 | -1.230772939 | 6.41E-07 | 1.16E-06 |
| *FHL5* | ENSG00000112214.9 | -1.226117138 | 4.43E-07 | 8.13E-07 |
| *NXPE4* | ENSG00000137634.8 | -1.225178527 | 3.88E-26 | 4.41E-25 |
| *ZNF667* | ENSG00000198046.10 | -1.22357474 | 9.83E-07 | 1.75E-06 |
| *PPP1R3C* | ENSG00000119938.8 | -1.223415027 | 4.96E-08 | 9.87E-08 |
| *ELMOD1* | ENSG00000110675.11 | -1.223349484 | 5.12E-05 | 7.94E-05 |
| *SLC23A3* | ENSG00000213901.9 | -1.223205773 | 5.92E-07 | 1.08E-06 |
| *ZCCHC12* | ENSG00000174460.3 | -1.222951376 | 3.81E-05 | 5.97E-05 |
| *ZSCAN1* | ENSG00000152467.8 | -1.222707463 | 2.88E-05 | 4.56E-05 |
| *PI15* | ENSG00000137558.6 | -1.221958153 | 3.79E-06 | 6.42E-06 |
| *ZNF418* | ENSG00000196724.11 | -1.221146008 | 2.99E-09 | 6.59E-09 |
| *CACNA2D1* | ENSG00000153956.14 | -1.220379765 | 3.26E-06 | 5.56E-06 |
| *ACKR1* | ENSG00000213088.8 | -1.220169441 | 4.57E-23 | 3.75E-22 |
| *CX3CR1* | ENSG00000168329.12 | -1.219600178 | 3.92E-11 | 1.02E-10 |
| *CA2* | ENSG00000104267.8 | -1.21890809 | 6.62E-28 | 9.17E-27 |
| *CDH23* | ENSG00000107736.18 | -1.217374417 | 2.45E-13 | 7.72E-13 |
| *DNAJC28* | ENSG00000177692.10 | -1.21583838 | 3.48E-23 | 2.89E-22 |
| *RFX6* | ENSG00000185002.8 | -1.214974615 | 0.000195492 | 0.000289298 |
| *SLC15A1* | ENSG00000088386.14 | -1.214301913 | 9.21E-06 | 1.51E-05 |
| *CCL19* | ENSG00000172724.10 | -1.21363014 | 4.62E-14 | 1.55E-13 |
| *NXPE1* | ENSG00000095110.6 | -1.213320363 | 5.39E-22 | 3.96E-21 |
| *PLCG2* | ENSG00000197943.8 | -1.212251569 | 3.64E-16 | 1.48E-15 |
| *SYNE3* | ENSG00000176438.11 | -1.211991331 | 1.95E-15 | 7.40E-15 |
| *MYOZ2* | ENSG00000172399.5 | -1.211818482 | 0.000244613 | 0.000359106 |
| *CLCA1* | ENSG00000016490.14 | -1.211642609 | 2.98E-43 | 1.48E-41 |
| *GRIK2* | ENSG00000164418.18 | -1.211081413 | 1.82E-06 | 3.17E-06 |
| *ELOVL4* | ENSG00000118402.5 | -1.21056173 | 5.42E-09 | 1.17E-08 |
| *DNAJB5* | ENSG00000137094.13 | -1.210471342 | 2.97E-07 | 5.53E-07 |
| *IL16* | ENSG00000172349.15 | -1.210188968 | 4.83E-17 | 2.13E-16 |
| *DMD* | ENSG00000198947.13 | -1.209346395 | 1.01E-07 | 1.95E-07 |
| *NIM1K* | ENSG00000177453.6 | -1.209284535 | 1.09E-09 | 2.50E-09 |
| *HDC* | ENSG00000140287.9 | -1.208791044 | 4.22E-10 | 1.00E-09 |
| *XCR1* | ENSG00000173578.7 | -1.208645575 | 7.45E-10 | 1.73E-09 |
| *CASP12* | ENSG00000204403.8 | -1.208374937 | 4.98E-07 | 9.11E-07 |
| *UNC13C* | ENSG00000137766.15 | -1.20789023 | 0.008639794 | 0.011174365 |
| *ZNF540* | ENSG00000171817.15 | -1.20744677 | 1.63E-13 | 5.21E-13 |
| *THEMIS* | ENSG00000172673.9 | -1.205552012 | 2.45E-09 | 5.45E-09 |
| *ZNF829* | ENSG00000185869.12 | -1.205101901 | 1.66E-14 | 5.80E-14 |
| *SLC4A1* | ENSG00000004939.12 | -1.204707565 | 0.008531701 | 0.011043797 |
| *ZNF257* | ENSG00000197134.10 | -1.204479763 | 1.71E-09 | 3.85E-09 |
| *SLC24A4* | ENSG00000140090.16 | -1.203240368 | 1.04E-09 | 2.38E-09 |
| *ZNF385D* | ENSG00000151789.8 | -1.202960479 | 6.01E-06 | 1.00E-05 |
| *IP6K3* | ENSG00000161896.9 | -1.201360482 | 6.16E-05 | 9.49E-05 |
| *NAT8B* | ENSG00000204872.3 | -1.199282044 | 1.41E-09 | 3.20E-09 |
| *C20orf194* | ENSG00000088854.12 | -1.198971608 | 1.36E-10 | 3.36E-10 |
| *NPY4R* | ENSG00000204174.5 | -1.197959459 | 2.09E-06 | 3.62E-06 |
| *TLX2* | ENSG00000115297.10 | -1.197650881 | 3.92E-09 | 8.54E-09 |
| *AQP12A* | ENSG00000184945.12 | -1.197495592 | 5.87E-06 | 9.80E-06 |
| *SLC9A3* | ENSG00000066230.9 | -1.196700908 | 3.17E-13 | 9.87E-13 |
| *INSM1* | ENSG00000173404.4 | -1.196618847 | 4.38E-10 | 1.04E-09 |
| *RMDN2* | ENSG00000115841.18 | -1.19615776 | 1.50E-23 | 1.29E-22 |
| *TPSG1* | ENSG00000116176.6 | -1.195970823 | 1.02E-24 | 9.96E-24 |
| *CORO6* | ENSG00000167549.17 | -1.195437817 | 0.000422255 | 0.000610051 |
| *SLITRK5* | ENSG00000165300.7 | -1.195376085 | 0.000575157 | 0.0008222 |
| *UCHL1* | ENSG00000154277.11 | -1.194112973 | 1.08E-15 | 4.20E-15 |
| *NR3C2* | ENSG00000151623.13 | -1.192943759 | 6.25E-40 | 2.50E-38 |
| *AKR1B10* | ENSG00000198074.8 | -1.192175898 | 9.33E-38 | 3.12E-36 |
| *NHSL2* | ENSG00000204131.7 | -1.190607138 | 0.000272653 | 0.000399247 |
| *UNC79* | ENSG00000133958.12 | -1.190579963 | 1.45E-16 | 6.12E-16 |
| *FGFR2* | ENSG00000066468.19 | -1.187927235 | 3.74E-32 | 7.86E-31 |
| *RNF180* | ENSG00000164197.10 | -1.187764952 | 3.82E-06 | 6.47E-06 |
| *ZNF831* | ENSG00000124203.5 | -1.187145102 | 6.24E-07 | 1.13E-06 |
| *MAB21L2* | ENSG00000181541.5 | -1.186010454 | 5.04E-10 | 1.19E-09 |
| *TNR* | ENSG00000116147.15 | -1.184421515 | 0.000560702 | 0.000802055 |
| *AP3B2* | ENSG00000103723.11 | -1.184415083 | 1.64E-06 | 2.86E-06 |
| *C1QTNF2* | ENSG00000145861.7 | -1.184202383 | 3.84E-17 | 1.71E-16 |
| *DCHS2* | ENSG00000197410.11 | -1.183751596 | 0.000998392 | 0.001401446 |
| *TCEAL7* | ENSG00000182916.7 | -1.182237284 | 3.05E-12 | 8.75E-12 |
| *DOCK3* | ENSG00000088538.12 | -1.180580472 | 0.001591168 | 0.002193902 |
| *SIGLEC1* | ENSG00000088827.11 | -1.180158948 | 3.62E-15 | 1.34E-14 |
| *ZNF671* | ENSG00000083814.11 | -1.179673275 | 1.34E-17 | 6.21E-17 |
| *KCNIP3* | ENSG00000115041.11 | -1.179463084 | 2.18E-05 | 3.48E-05 |
| *CYP2C18* | ENSG00000108242.11 | -1.179005158 | 2.43E-09 | 5.40E-09 |
| *MEOX2* | ENSG00000106511.5 | -1.178986564 | 2.02E-07 | 3.81E-07 |
| *DAAM2* | ENSG00000146122.15 | -1.178584938 | 3.08E-21 | 2.09E-20 |
| *CD79B* | ENSG00000007312.11 | -1.175569858 | 5.38E-12 | 1.51E-11 |
| *ESR1* | ENSG00000091831.20 | -1.175561007 | 2.81E-14 | 9.62E-14 |
| *LDHD* | ENSG00000166816.12 | -1.174834227 | 3.84E-31 | 7.23E-30 |
| *BNC2* | ENSG00000173068.16 | -1.173907329 | 7.51E-06 | 1.24E-05 |
| *ROS1* | ENSG00000047936.9 | -1.173104443 | 5.37E-08 | 1.06E-07 |
| *RANBP3L* | ENSG00000164188.7 | -1.171125552 | 0.002530189 | 0.003432322 |
| *CCDC169* | ENSG00000242715.6 | -1.170321906 | 0.004554489 | 0.006042698 |
| *COL25A1* | ENSG00000188517.13 | -1.17027674 | 1.85E-05 | 2.97E-05 |
| *CD180* | ENSG00000134061.5 | -1.17024248 | 4.19E-12 | 1.19E-11 |
| *CYSLTR2* | ENSG00000152207.6 | -1.169957897 | 8.75E-14 | 2.86E-13 |
| *OMG* | ENSG00000126861.4 | -1.16959975 | 4.78E-05 | 7.43E-05 |
| *MAOB* | ENSG00000069535.13 | -1.16892432 | 1.46E-14 | 5.12E-14 |
| *EML1* | ENSG00000066629.15 | -1.168736328 | 1.79E-08 | 3.69E-08 |
| *BTNL3* | ENSG00000168903.8 | -1.16836217 | 2.21E-27 | 2.91E-26 |
| *ZNF575* | ENSG00000176472.9 | -1.167359546 | 7.57E-16 | 2.99E-15 |
| *BACH2* | ENSG00000112182.13 | -1.166977759 | 0.000100347 | 0.000152273 |
| *ZNF568* | ENSG00000198453.11 | -1.166462888 | 1.03E-15 | 4.01E-15 |
| *THBS4* | ENSG00000113296.13 | -1.165305167 | 1.70E-05 | 2.74E-05 |
| *BARX2* | ENSG00000043039.6 | -1.163586089 | 1.36E-20 | 8.50E-20 |
| *MEIS1* | ENSG00000143995.18 | -1.162659064 | 4.23E-09 | 9.19E-09 |
| *FAM153A* | ENSG00000170074.18 | -1.161964065 | 0.010014143 | 0.012872783 |
| *ACKR4* | ENSG00000129048.6 | -1.161459318 | 3.41E-06 | 5.80E-06 |
| *FXYD6* | ENSG00000137726.14 | -1.160743403 | 2.48E-10 | 6.01E-10 |
| *TUBAL3* | ENSG00000178462.10 | -1.160532294 | 1.35E-22 | 1.06E-21 |
| *SLC10A1* | ENSG00000100652.4 | -1.160178739 | 1.04E-10 | 2.60E-10 |
| *CXCL12* | ENSG00000107562.15 | -1.159215215 | 7.89E-29 | 1.19E-27 |
| *MYLK* | ENSG00000065534.17 | -1.159181761 | 9.51E-14 | 3.10E-13 |
| *ZNF415* | ENSG00000170954.10 | -1.15916361 | 7.79E-21 | 5.02E-20 |
| *TMIE* | ENSG00000181585.3 | -1.158283283 | 2.93E-08 | 5.93E-08 |
| *LOXL4* | ENSG00000138131.3 | -1.157665994 | 1.55E-07 | 2.95E-07 |
| *ROR1* | ENSG00000185483.10 | -1.157575962 | 2.99E-09 | 6.59E-09 |
| *LY6H* | ENSG00000176956.11 | -1.156554293 | 0.004407937 | 0.00585831 |
| *B3GNT7* | ENSG00000156966.6 | -1.156421123 | 7.25E-18 | 3.43E-17 |
| *GPRASP1* | ENSG00000198932.11 | -1.155491273 | 8.41E-09 | 1.78E-08 |
| *MYL2* | ENSG00000111245.13 | -1.155433904 | 1.98E-05 | 3.17E-05 |
| *HPGD* | ENSG00000164120.12 | -1.155321344 | 4.56E-36 | 1.35E-34 |
| *LRRK2* | ENSG00000188906.12 | -1.154128413 | 6.40E-10 | 1.49E-09 |
| *FLVCR2* | ENSG00000119686.8 | -1.15328392 | 5.49E-19 | 2.89E-18 |
| *PRKAR2B* | ENSG00000005249.11 | -1.153123245 | 3.20E-29 | 5.04E-28 |
| *PREX2* | ENSG00000046889.17 | -1.152579752 | 1.58E-13 | 5.05E-13 |
| *TYRP1* | ENSG00000107165.11 | -1.151999563 | 6.18E-07 | 1.12E-06 |
| *ZSCAN23* | ENSG00000187987.8 | -1.151966239 | 1.95E-05 | 3.13E-05 |
| *FAM124A* | ENSG00000150510.14 | -1.151592696 | 3.58E-05 | 5.62E-05 |
| *ART4* | ENSG00000111339.9 | -1.151543061 | 2.19E-06 | 3.79E-06 |
| *PNLIPRP2* | ENSG00000266200.5 | -1.150670215 | 1.78E-06 | 3.10E-06 |
| *ICAM3* | ENSG00000076662.8 | -1.150326075 | 6.17E-14 | 2.05E-13 |
| *RUNX1T1* | ENSG00000079102.15 | -1.149999465 | 1.45E-06 | 2.55E-06 |
| *PCK1* | ENSG00000124253.10 | -1.149780575 | 8.83E-20 | 5.07E-19 |
| *PEBP4* | ENSG00000134020.7 | -1.149618035 | 8.49E-07 | 1.52E-06 |
| *SVEP1* | ENSG00000165124.16 | -1.149048124 | 2.31E-10 | 5.61E-10 |
| *TGM4* | ENSG00000163810.10 | -1.145367232 | 2.53E-06 | 4.35E-06 |
| *GIMAP1* | ENSG00000213203.2 | -1.144834745 | 1.10E-19 | 6.26E-19 |
| *PPARGC1A* | ENSG00000109819.7 | -1.144803603 | 2.64E-25 | 2.76E-24 |
| *APLP1* | ENSG00000105290.10 | -1.144349896 | 1.37E-10 | 3.39E-10 |
| *CDK5R2* | ENSG00000171450.5 | -1.143568818 | 6.37E-08 | 1.25E-07 |
| *TIMD4* | ENSG00000145850.7 | -1.143081049 | 0.000848852 | 0.001198717 |
| *TSPAN7* | ENSG00000156298.11 | -1.141821149 | 5.03E-46 | 3.09E-44 |
| *RD3* | ENSG00000198570.5 | -1.140804867 | 0.00331023 | 0.004445642 |
| *PADI2* | ENSG00000117115.11 | -1.140711141 | 1.35E-28 | 2.00E-27 |
| *PDE3A* | ENSG00000172572.6 | -1.1390136 | 7.55E-36 | 2.17E-34 |
| *DACH2* | ENSG00000126733.19 | -1.137838204 | 0.000743199 | 0.001054138 |
| *SCHIP1* | ENSG00000151967.17 | -1.136354464 | 0.000256505 | 0.000376029 |
| *LPAR1* | ENSG00000198121.12 | -1.136253737 | 1.56E-51 | 1.23E-49 |
| *GDF5* | ENSG00000125965.7 | -1.13507159 | 0.037104003 | 0.045181522 |
| *MEIS2* | ENSG00000134138.18 | -1.132299988 | 1.07E-05 | 1.75E-05 |
| *CACNA1A* | ENSG00000141837.17 | -1.131651172 | 1.17E-06 | 2.07E-06 |
| *C16orf54* | ENSG00000185905.3 | -1.131609047 | 9.14E-18 | 4.29E-17 |
| *RASGRP4* | ENSG00000171777.14 | -1.130995645 | 1.65E-14 | 5.77E-14 |
| *GPR174* | ENSG00000147138.1 | -1.130342164 | 1.50E-09 | 3.40E-09 |
| *NAALAD2* | ENSG00000077616.9 | -1.130051099 | 3.10E-10 | 7.45E-10 |
| *SGSM1* | ENSG00000167037.17 | -1.129592817 | 2.09E-12 | 6.07E-12 |
| *KRT12* | ENSG00000187242.4 | -1.128626259 | 1.53E-07 | 2.91E-07 |
| *PM20D1* | ENSG00000162877.11 | -1.128493594 | 3.46E-05 | 5.44E-05 |
| *LRRN4CL* | ENSG00000177363.4 | -1.128438825 | 2.16E-14 | 7.47E-14 |
| *ARHGAP44* | ENSG00000006740.15 | -1.127619704 | 6.35E-20 | 3.71E-19 |
| *CD207* | ENSG00000116031.8 | -1.127472463 | 5.00E-10 | 1.18E-09 |
| *ZNF582* | ENSG00000018869.15 | -1.126631593 | 3.96E-14 | 1.34E-13 |
| *SULT4A1* | ENSG00000130540.12 | -1.126546047 | 9.45E-06 | 1.55E-05 |
| *COL28A1* | ENSG00000215018.8 | -1.126425965 | 8.40E-10 | 1.94E-09 |
| *SLC28A2* | ENSG00000137860.10 | -1.12639922 | 0.000212847 | 0.000313932 |
| *FGL2* | ENSG00000127951.5 | -1.126226415 | 5.88E-42 | 2.73E-40 |
| *LIPJ* | ENSG00000204022.8 | -1.126124678 | 2.75E-05 | 4.36E-05 |
| *TSHR* | ENSG00000165409.14 | -1.126064865 | 9.17E-07 | 1.64E-06 |
| *ESR2* | ENSG00000140009.17 | -1.125991651 | 6.17E-07 | 1.12E-06 |
| *PTGS1* | ENSG00000095303.13 | -1.125270035 | 3.09E-16 | 1.27E-15 |
| *GLI3* | ENSG00000106571.11 | -1.12488324 | 1.28E-06 | 2.26E-06 |
| *MIPOL1* | ENSG00000151338.17 | -1.124334508 | 2.05E-14 | 7.10E-14 |
| *SYNE1* | ENSG00000131018.21 | -1.123902034 | 5.19E-11 | 1.34E-10 |
| *HSPB2* | ENSG00000170276.5 | -1.123812483 | 7.11E-08 | 1.39E-07 |
| *ZBTB7C* | ENSG00000184828.8 | -1.122152625 | 5.49E-25 | 5.54E-24 |
| *KLRC1* | ENSG00000134545.12 | -1.120546843 | 4.40E-06 | 7.42E-06 |
| *CPNE5* | ENSG00000124772.10 | -1.119853572 | 2.52E-17 | 1.14E-16 |
| *GPR55* | ENSG00000135898.8 | -1.119407571 | 5.64E-08 | 1.12E-07 |
| *NPR1* | ENSG00000169418.9 | -1.119310828 | 3.86E-12 | 1.10E-11 |
| *KIT* | ENSG00000157404.14 | -1.119244332 | 2.53E-23 | 2.12E-22 |
| *FGF2* | ENSG00000138685.11 | -1.11913228 | 8.97E-08 | 1.74E-07 |
| *IGSF5* | ENSG00000183067.5 | -1.119106943 | 0.0019492 | 0.002671599 |
| *KANK4* | ENSG00000132854.17 | -1.118232697 | 9.56E-11 | 2.40E-10 |
| *SNCG* | ENSG00000173267.12 | -1.117322077 | 2.58E-16 | 1.07E-15 |
| *TLR3* | ENSG00000164342.11 | -1.117027262 | 3.14E-23 | 2.62E-22 |
| *APCDD1L* | ENSG00000198768.9 | -1.11660282 | 3.66E-06 | 6.21E-06 |
| *ADAMDEC1* | ENSG00000134028.13 | -1.115411161 | 1.13E-24 | 1.10E-23 |
| *KIAA1211* | ENSG00000109265.11 | -1.114655512 | 1.21E-20 | 7.62E-20 |
| *MTUS2* | ENSG00000132938.17 | -1.113970292 | 0.002770568 | 0.003742977 |
| *GNG2* | ENSG00000186469.7 | -1.11234995 | 4.95E-23 | 4.05E-22 |
| *RBFOX1* | ENSG00000078328.18 | -1.110527022 | 1.41E-08 | 2.94E-08 |
| *AMHR2* | ENSG00000135409.9 | -1.110366291 | 3.33E-06 | 5.67E-06 |
| *RASGRP2* | ENSG00000068831.17 | -1.11003911 | 1.42E-11 | 3.84E-11 |
| *FAM177B* | ENSG00000197520.9 | -1.109736818 | 3.26E-09 | 7.15E-09 |
| *PALM* | ENSG00000099864.16 | -1.109648991 | 2.43E-16 | 1.01E-15 |
| *MESP2* | ENSG00000188095.4 | -1.109428762 | 1.05E-13 | 3.41E-13 |
| *WNT9A* | ENSG00000143816.7 | -1.106256476 | 2.57E-09 | 5.70E-09 |
| *GABRB3* | ENSG00000166206.12 | -1.105551814 | 6.42E-11 | 1.63E-10 |
| *CPXM2* | ENSG00000121898.11 | -1.105519146 | 8.60E-09 | 1.82E-08 |
| *BDKRB1* | ENSG00000100739.9 | -1.102790103 | 8.08E-17 | 3.49E-16 |
| *SPTBN4* | ENSG00000160460.14 | -1.102326836 | 2.31E-05 | 3.68E-05 |
| *DPYD* | ENSG00000188641.11 | -1.101589864 | 8.73E-29 | 1.31E-27 |
| *CEBPE* | ENSG00000092067.5 | -1.101151159 | 0.000792618 | 0.001122071 |
| *S100B* | ENSG00000160307.8 | -1.100473084 | 5.25E-13 | 1.60E-12 |
| *FAM19A2* | ENSG00000198673.9 | -1.099702522 | 2.49E-10 | 6.03E-10 |
| *ZFPM2* | ENSG00000169946.12 | -1.098076595 | 3.06E-09 | 6.73E-09 |
| *AKAP2* | ENSG00000241978.8 | -1.097844568 | 2.92E-08 | 5.91E-08 |
| *EBF1* | ENSG00000164330.15 | -1.097622937 | 1.49E-10 | 3.67E-10 |
| *TNNI3K* | ENSG00000116783.13 | -1.096625035 | 0.001669419 | 0.002300154 |
| *OTC* | ENSG00000036473.6 | -1.095916335 | 7.13E-11 | 1.81E-10 |
| *IGLL1* | ENSG00000128322.6 | -1.095296566 | 9.82E-05 | 0.000149103 |
| *CD79A* | ENSG00000105369.8 | -1.094983915 | 1.83E-18 | 9.20E-18 |
| *FCN1* | ENSG00000085265.9 | -1.094886347 | 3.33E-05 | 5.24E-05 |
| *HCN4* | ENSG00000138622.3 | -1.094259205 | 0.001235998 | 0.001719988 |
| *DPY19L2* | ENSG00000177990.10 | -1.09177283 | 0.000168392 | 0.000250491 |
| *MTNR1A* | ENSG00000168412.6 | -1.091769645 | 4.25E-11 | 1.10E-10 |
| *PLA2G2D* | ENSG00000117215.13 | -1.091375073 | 3.92E-06 | 6.63E-06 |
| *SIGLEC16* | ENSG00000161643.11 | -1.091279956 | 2.03E-05 | 3.25E-05 |
| *NXPH3* | ENSG00000182575.7 | -1.091080545 | 1.21E-06 | 2.14E-06 |
| *TRIM61* | ENSG00000183439.6 | -1.090651268 | 6.68E-07 | 1.21E-06 |
| *CACNB2* | ENSG00000165995.17 | -1.088959002 | 1.54E-17 | 7.10E-17 |
| *ZNF793* | ENSG00000188227.11 | -1.087098576 | 4.98E-11 | 1.28E-10 |
| *LRRTM1* | ENSG00000162951.9 | -1.086859767 | 0.000181537 | 0.000269164 |
| *TMIGD2* | ENSG00000167664.7 | -1.085754949 | 3.54E-10 | 8.48E-10 |
| *CEACAM7* | ENSG00000007306.13 | -1.085173937 | 7.35E-26 | 8.14E-25 |
| *LAX1* | ENSG00000122188.11 | -1.082159225 | 4.23E-11 | 1.10E-10 |
| *P2RY10* | ENSG00000078589.11 | -1.080098015 | 3.61E-12 | 1.03E-11 |
| *ITLN1* | ENSG00000179914.4 | -1.079479074 | 1.57E-26 | 1.89E-25 |
| *SLC18A2* | ENSG00000165646.10 | -1.079020475 | 3.88E-11 | 1.01E-10 |
| *LRRTM2* | ENSG00000146006.7 | -1.079009068 | 2.94E-09 | 6.48E-09 |
| *SLC16A9* | ENSG00000165449.10 | -1.078751478 | 1.12E-13 | 3.63E-13 |
| *TMEM132C* | ENSG00000181234.9 | -1.076139943 | 7.69E-06 | 1.27E-05 |
| *MEI1* | ENSG00000167077.11 | -1.075959152 | 2.90E-10 | 6.99E-10 |
| *LRRC19* | ENSG00000184434.7 | -1.07581372 | 1.80E-29 | 2.89E-28 |
| *RASD2* | ENSG00000100302.6 | -1.075287659 | 4.02E-23 | 3.31E-22 |
| *CDC14A* | ENSG00000079335.16 | -1.07212226 | 2.17E-19 | 1.20E-18 |
| *KLRB1* | ENSG00000111796.3 | -1.072049173 | 2.98E-19 | 1.62E-18 |
| *NBEAL1* | ENSG00000144426.17 | -1.071573763 | 1.07E-18 | 5.48E-18 |
| *SULT1A1* | ENSG00000196502.10 | -1.070576678 | 4.94E-18 | 2.38E-17 |
| *CNGA1* | ENSG00000198515.12 | -1.069515961 | 4.47E-10 | 1.06E-09 |
| *ANK3* | ENSG00000151150.19 | -1.06941782 | 1.75E-18 | 8.81E-18 |
| *NKX3-2* | ENSG00000109705.7 | -1.069321549 | 0.000174536 | 0.000259231 |
| *CD1C* | ENSG00000158481.11 | -1.067896877 | 2.07E-10 | 5.04E-10 |
| *FGF14* | ENSG00000102466.14 | -1.065863885 | 4.42E-06 | 7.45E-06 |
| *CPA2* | ENSG00000158516.10 | -1.065733962 | 0.000423759 | 0.000612052 |
| *PDZK1* | ENSG00000174827.12 | -1.064058909 | 0.002532329 | 0.003434924 |
| *ITIH5* | ENSG00000123243.13 | -1.063923597 | 9.31E-14 | 3.03E-13 |
| *MT1F* | ENSG00000198417.6 | -1.063658135 | 1.46E-15 | 5.60E-15 |
| *BCAS1* | ENSG00000064787.11 | -1.062489716 | 1.28E-29 | 2.07E-28 |
| *FOLR2* | ENSG00000165457.12 | -1.062267404 | 4.86E-22 | 3.60E-21 |
| *VSIG2* | ENSG00000019102.10 | -1.061957846 | 1.70E-26 | 2.03E-25 |
| *KRBOX1* | ENSG00000240747.6 | -1.060908718 | 5.07E-05 | 7.87E-05 |
| *TRAT1* | ENSG00000163519.12 | -1.060815896 | 6.12E-10 | 1.43E-09 |
| *TNFRSF13C* | ENSG00000159958.4 | -1.060069234 | 1.05E-05 | 1.72E-05 |
| *CCDC68* | ENSG00000166510.12 | -1.059255493 | 5.62E-26 | 6.31E-25 |
| *CFL2* | ENSG00000165410.13 | -1.058924213 | 2.40E-08 | 4.90E-08 |
| *RAB6B* | ENSG00000154917.9 | -1.05866911 | 1.20E-12 | 3.56E-12 |
| *POPDC3* | ENSG00000132429.8 | -1.058448112 | 0.004299391 | 0.005720931 |
| *DES* | ENSG00000175084.10 | -1.057616383 | 3.87E-15 | 1.43E-14 |
| *PPP1R16B* | ENSG00000101445.8 | -1.057213611 | 2.24E-19 | 1.23E-18 |
| *MLIP* | ENSG00000146147.13 | -1.054915305 | 1.05E-05 | 1.72E-05 |
| *SERTAD4* | ENSG00000082497.10 | -1.054026255 | 6.17E-12 | 1.72E-11 |
| *SULT1B1* | ENSG00000173597.7 | -1.053998203 | 4.16E-36 | 1.24E-34 |
| *DPEP2* | ENSG00000167261.12 | -1.053358173 | 1.88E-15 | 7.15E-15 |
| *TENM2* | ENSG00000145934.14 | -1.052113844 | 0.001488067 | 0.00205633 |
| *CCDC69* | ENSG00000198624.11 | -1.051710535 | 7.51E-17 | 3.25E-16 |
| *KLHL6* | ENSG00000172578.10 | -1.051396532 | 5.63E-09 | 1.21E-08 |
| *ADRB1* | ENSG00000043591.5 | -1.050482328 | 3.69E-15 | 1.37E-14 |
| *LTK* | ENSG00000062524.14 | -1.0503719 | 1.08E-13 | 3.51E-13 |
| *USP51* | ENSG00000247746.4 | -1.050354972 | 2.87E-11 | 7.56E-11 |
| *TMEM37* | ENSG00000171227.6 | -1.048538981 | 2.35E-23 | 1.98E-22 |
| *NCF1* | ENSG00000158517.12 | -1.048354134 | 1.04E-11 | 2.85E-11 |
| *AKAP5* | ENSG00000179841.8 | -1.048207221 | 3.69E-17 | 1.64E-16 |
| *NR3C1* | ENSG00000113580.13 | -1.047795014 | 1.58E-32 | 3.39E-31 |
| *FGF13* | ENSG00000129682.12 | -1.046803195 | 1.03E-05 | 1.69E-05 |
| *TMEM61* | ENSG00000143001.4 | -1.046570275 | 1.48E-16 | 6.24E-16 |
| *RSPO3* | ENSG00000146374.12 | -1.046442005 | 1.09E-11 | 2.98E-11 |
| *ANKRD65* | ENSG00000235098.7 | -1.046364375 | 5.74E-09 | 1.23E-08 |
| *OR51E2* | ENSG00000167332.7 | -1.045987664 | 0.000232543 | 0.000341841 |
| *PDE9A* | ENSG00000160191.16 | -1.045442787 | 1.07E-24 | 1.04E-23 |
| *KIAA0513* | ENSG00000135709.11 | -1.044568542 | 7.73E-29 | 1.17E-27 |
| *PDE1B* | ENSG00000123360.10 | -1.043935801 | 5.12E-10 | 1.20E-09 |
| *AMER3* | ENSG00000178171.9 | -1.043274137 | 0.00021576 | 0.000318097 |
| *IKZF1* | ENSG00000185811.15 | -1.042671815 | 7.19E-15 | 2.60E-14 |
| *ZNF528* | ENSG00000167555.12 | -1.041567825 | 8.20E-17 | 3.54E-16 |
| *SLC22A17* | ENSG00000092096.13 | -1.040973116 | 2.84E-15 | 1.06E-14 |
| *MALL* | ENSG00000144063.3 | -1.040408197 | 6.64E-26 | 7.41E-25 |
| *LURAP1* | ENSG00000171357.5 | -1.039729929 | 2.92E-05 | 4.62E-05 |
| *PPP1R12B* | ENSG00000077157.19 | -1.038660134 | 7.85E-11 | 1.99E-10 |
| *NCAN* | ENSG00000130287.12 | -1.03860559 | 0.019501116 | 0.024423375 |
| *TRIM63* | ENSG00000158022.6 | -1.037602059 | 9.14E-06 | 1.50E-05 |
| *PDE1A* | ENSG00000115252.17 | -1.036685277 | 2.75E-08 | 5.59E-08 |
| *EFNA5* | ENSG00000184349.11 | -1.036602903 | 5.91E-22 | 4.33E-21 |
| *GP9* | ENSG00000169704.4 | -1.034358871 | 5.89E-08 | 1.16E-07 |
| *ZNF804A* | ENSG00000170396.7 | -1.033906538 | 1.56E-05 | 2.52E-05 |
| *SLC4A9* | ENSG00000113073.13 | -1.033396359 | 0.001284146 | 0.00178378 |
| *GPR146* | ENSG00000164849.8 | -1.033361057 | 1.31E-08 | 2.73E-08 |
| *SLC9A2* | ENSG00000115616.2 | -1.03140059 | 1.76E-27 | 2.33E-26 |
| *TREH* | ENSG00000118094.10 | -1.030615673 | 0.000267893 | 0.000392426 |
| *MEF2C* | ENSG00000081189.12 | -1.030554639 | 5.01E-25 | 5.08E-24 |
| *ZNF304* | ENSG00000131845.13 | -1.030026836 | 2.55E-20 | 1.54E-19 |
| *SYT2* | ENSG00000143858.10 | -1.029523566 | 3.09E-08 | 6.25E-08 |
| *ACACB* | ENSG00000076555.14 | -1.029029184 | 3.88E-22 | 2.90E-21 |
| *P2RY8* | ENSG00000182162.8 | -1.028494208 | 1.98E-12 | 5.77E-12 |
| *CACNA1B* | ENSG00000148408.11 | -1.028231213 | 0.000412019 | 0.000595652 |
| *POU2F2* | ENSG00000028277.19 | -1.028169137 | 1.28E-09 | 2.92E-09 |
| *PLAC8* | ENSG00000145287.9 | -1.028106654 | 2.11E-28 | 3.07E-27 |
| *CNN1* | ENSG00000130176.6 | -1.027915722 | 2.71E-11 | 7.15E-11 |
| *CD1D* | ENSG00000158473.6 | -1.027565179 | 7.72E-19 | 4.02E-18 |
| *ZEB2* | ENSG00000169554.15 | -1.024452306 | 2.59E-25 | 2.70E-24 |
| *MAP3K19* | ENSG00000176601.10 | -1.024002249 | 0.000220132 | 0.000324337 |
| *RGS18* | ENSG00000150681.8 | -1.023950243 | 9.62E-09 | 2.03E-08 |
| *ARHGAP28* | ENSG00000088756.11 | -1.023903036 | 7.89E-14 | 2.59E-13 |
| *ST6GALNAC3* | ENSG00000184005.10 | -1.02343177 | 2.66E-11 | 7.03E-11 |
| *CD33* | ENSG00000105383.13 | -1.023339187 | 4.72E-09 | 1.02E-08 |
| *CYP26B1* | ENSG00000003137.7 | -1.023289099 | 4.69E-12 | 1.32E-11 |
| *FLRT2* | ENSG00000185070.9 | -1.022631361 | 3.61E-07 | 6.68E-07 |
| *CHD5* | ENSG00000116254.16 | -1.021770858 | 0.000446188 | 0.000643546 |
| *XDH* | ENSG00000158125.8 | -1.021227588 | 1.55E-19 | 8.68E-19 |
| *SLC1A2* | ENSG00000110436.10 | -1.021164065 | 0.001036681 | 0.001452687 |
| *FBXO27* | ENSG00000161243.7 | -1.021073295 | 5.17E-06 | 8.66E-06 |
| *SLC7A3* | ENSG00000165349.10 | -1.021025905 | 1.42E-05 | 2.30E-05 |
| *C7orf31* | ENSG00000153790.10 | -1.021007873 | 6.99E-22 | 5.08E-21 |
| *CLDN5* | ENSG00000184113.9 | -1.019972467 | 8.77E-25 | 8.64E-24 |
| *PLCE1* | ENSG00000138193.13 | -1.019709436 | 9.87E-25 | 9.65E-24 |
| *PTGIS* | ENSG00000124212.5 | -1.019584056 | 2.83E-10 | 6.82E-10 |
| *UST* | ENSG00000111962.7 | -1.018388001 | 1.66E-11 | 4.46E-11 |
| *DDR2* | ENSG00000162733.15 | -1.018189294 | 2.15E-12 | 6.24E-12 |
| *KLF8* | ENSG00000102349.13 | -1.018001282 | 6.69E-11 | 1.70E-10 |
| *ARL4D* | ENSG00000175906.4 | -1.016501842 | 2.12E-09 | 4.74E-09 |
| *NYAP1* | ENSG00000166924.7 | -1.015728116 | 1.02E-07 | 1.97E-07 |
| *GPR156* | ENSG00000175697.9 | -1.014668857 | 0.030907716 | 0.037944018 |
| *BCL2* | ENSG00000171791.11 | -1.014282231 | 7.28E-29 | 1.10E-27 |
| *ITM2A* | ENSG00000078596.9 | -1.012958434 | 2.33E-30 | 4.06E-29 |
| *IL1R2* | ENSG00000115590.12 | -1.012630819 | 1.00E-15 | 3.90E-15 |
| *ARHGEF25* | ENSG00000240771.5 | -1.012630072 | 8.66E-09 | 1.83E-08 |
| *VEPH1* | ENSG00000197415.10 | -1.012545876 | 3.07E-09 | 6.75E-09 |
| *NFASC* | ENSG00000163531.14 | -1.010732361 | 5.09E-10 | 1.20E-09 |
| *SECTM1* | ENSG00000141574.6 | -1.00965327 | 1.35E-22 | 1.06E-21 |
| *NLGN3* | ENSG00000196338.11 | -1.009587509 | 5.70E-09 | 1.22E-08 |
| *FKBP1B* | ENSG00000119782.12 | -1.009326729 | 5.18E-18 | 2.49E-17 |
| *RCSD1* | ENSG00000198771.9 | -1.009148943 | 9.01E-24 | 7.95E-23 |
| *GOLGA8N* | ENSG00000232653.7 | -1.008552119 | 1.52E-08 | 3.15E-08 |
| *TBX10* | ENSG00000167800.9 | -1.008410892 | 7.83E-10 | 1.82E-09 |
| *STON1* | ENSG00000243244.4 | -1.008387765 | 1.23E-05 | 2.00E-05 |
| *KLHL4* | ENSG00000102271.12 | -1.008238566 | 5.71E-07 | 1.04E-06 |
| *FMO4* | ENSG00000076258.8 | -1.007526544 | 1.21E-20 | 7.62E-20 |
| *CHI3L2* | ENSG00000064886.12 | -1.00684822 | 7.30E-05 | 0.000111895 |
| *RCAN2* | ENSG00000172348.13 | -1.006671822 | 2.74E-29 | 4.35E-28 |
| *RAB37* | ENSG00000172794.18 | -1.006472678 | 9.07E-15 | 3.24E-14 |
| *FRMD1* | ENSG00000153303.15 | -1.005565287 | 5.33E-13 | 1.63E-12 |
| *AHRR* | ENSG00000063438.15 | -1.005423226 | 1.00E-09 | 2.30E-09 |
| *BOC* | ENSG00000144857.13 | -1.005096143 | 7.97E-08 | 1.55E-07 |
| *CACNA2D3* | ENSG00000157445.13 | -1.004862015 | 8.18E-07 | 1.47E-06 |
| *NEURL1* | ENSG00000107954.9 | -1.004385004 | 8.32E-20 | 4.79E-19 |
| *ADAM11* | ENSG00000073670.12 | -1.004055086 | 0.003140956 | 0.004225646 |
| *CNNM2* | ENSG00000148842.16 | -1.003224244 | 1.49E-18 | 7.54E-18 |
| *KLK13* | ENSG00000167759.11 | -1.003216375 | 7.98E-05 | 0.000121967 |
| *PTCRA* | ENSG00000171611.8 | -1.002219593 | 1.13E-05 | 1.85E-05 |
| *BTK* | ENSG00000010671.14 | -1.00188677 | 3.19E-14 | 1.09E-13 |
| *PRICKLE2* | ENSG00000163637.10 | -1.001134102 | 2.40E-07 | 4.50E-07 |
| *SLC47A1* | ENSG00000142494.12 | -1.000879381 | 1.41E-11 | 3.81E-11 |
| *TRIM59* | ENSG00000213186.6 | 1.000353354 | 1.82E-32 | 3.90E-31 |
| *RTN4R* | ENSG00000040608.12 | 1.000535601 | 2.89E-31 | 5.52E-30 |
| *SYN3* | ENSG00000185666.13 | 1.000706409 | 1.20E-07 | 2.31E-07 |
| *CXCL2* | ENSG00000081041.8 | 1.001828213 | 4.39E-19 | 2.34E-18 |
| *SRRM5* | ENSG00000226763.4 | 1.001928276 | 2.74E-19 | 1.49E-18 |
| *VWCE* | ENSG00000167992.11 | 1.002646661 | 7.37E-10 | 1.71E-09 |
| *TNFRSF12A* | ENSG00000006327.12 | 1.003006314 | 4.93E-24 | 4.50E-23 |
| *FCRLB* | ENSG00000162746.13 | 1.003494963 | 8.96E-15 | 3.21E-14 |
| *RNF32* | ENSG00000105982.15 | 1.004486304 | 3.27E-48 | 2.28E-46 |
| *SERPINE1* | ENSG00000106366.8 | 1.004665794 | 4.90E-13 | 1.50E-12 |
| *WFDC3* | ENSG00000124116.17 | 1.005551192 | 4.27E-14 | 1.44E-13 |
| *HMGA2* | ENSG00000149948.12 | 1.005822734 | 2.88E-27 | 3.72E-26 |
| *ARMC2* | ENSG00000118690.11 | 1.006477343 | 8.99E-46 | 5.37E-44 |
| *FAM19A5* | ENSG00000219438.7 | 1.006502218 | 6.13E-16 | 2.45E-15 |
| *CKAP2L* | ENSG00000169607.11 | 1.007007389 | 1.94E-25 | 2.05E-24 |
| *ZSCAN4* | ENSG00000180532.9 | 1.009267008 | 6.21E-05 | 9.57E-05 |
| *CXCL1* | ENSG00000163739.4 | 1.010321394 | 1.37E-19 | 7.72E-19 |
| *ENTHD1* | ENSG00000176177.9 | 1.010823766 | 2.49E-06 | 4.29E-06 |
| *NEIL3* | ENSG00000109674.3 | 1.011412069 | 5.41E-23 | 4.41E-22 |
| *DMKN* | ENSG00000161249.19 | 1.011844424 | 7.84E-25 | 7.78E-24 |
| *EXO1* | ENSG00000174371.15 | 1.012065727 | 4.47E-24 | 4.10E-23 |
| *PSD2* | ENSG00000146005.3 | 1.014723451 | 3.08E-09 | 6.77E-09 |
| *ZNF280C* | ENSG00000056277.14 | 1.015749619 | 8.31E-49 | 5.84E-47 |
| *PKMYT1* | ENSG00000127564.15 | 1.016738866 | 2.90E-27 | 3.75E-26 |
| *PIP5KL1* | ENSG00000167103.10 | 1.01683298 | 1.24E-13 | 4.00E-13 |
| *ATP6V1E2* | ENSG00000250565.5 | 1.017193647 | 1.20E-27 | 1.62E-26 |
| *CLVS1* | ENSG00000177182.9 | 1.017555894 | 0.000195903 | 0.000289853 |
| *ADAT2* | ENSG00000189007.14 | 1.017829909 | 5.51E-35 | 1.47E-33 |
| *NUF2* | ENSG00000143228.11 | 1.018169079 | 2.80E-28 | 4.00E-27 |
| *CPLX1* | ENSG00000168993.13 | 1.018614953 | 8.70E-20 | 5.00E-19 |
| *SYCP2* | ENSG00000196074.11 | 1.018620412 | 1.95E-10 | 4.76E-10 |
| *CCDC40* | ENSG00000141519.13 | 1.019193391 | 2.36E-16 | 9.78E-16 |
| *PRSS53* | ENSG00000151006.7 | 1.020035988 | 2.59E-12 | 7.47E-12 |
| *DACH1* | ENSG00000276644.3 | 1.02284418 | 2.26E-45 | 1.32E-43 |
| *ZNF850* | ENSG00000267041.4 | 1.024362123 | 8.84E-34 | 2.13E-32 |
| *PSAT1* | ENSG00000135069.12 | 1.025277333 | 1.37E-37 | 4.54E-36 |
| *IPO4* | ENSG00000196497.14 | 1.025727654 | 2.16E-37 | 6.97E-36 |
| *ASIC3* | ENSG00000213199.6 | 1.026037749 | 4.51E-21 | 2.99E-20 |
| *RFX4* | ENSG00000111783.11 | 1.02644005 | 3.75E-06 | 6.36E-06 |
| *LRRC34* | ENSG00000171757.14 | 1.026586563 | 4.39E-22 | 3.27E-21 |
| *DUSP14* | ENSG00000276023.3 | 1.026666227 | 4.28E-24 | 3.93E-23 |
| *RECQL4* | ENSG00000160957.11 | 1.027927919 | 5.12E-37 | 1.63E-35 |
| *FZD9* | ENSG00000188763.4 | 1.028037363 | 2.12E-11 | 5.65E-11 |
| *HILPDA* | ENSG00000135245.9 | 1.028050763 | 3.02E-38 | 1.05E-36 |
| *LBP* | ENSG00000129988.5 | 1.029078663 | 0.022228104 | 0.02769961 |
| *FOXP3* | ENSG00000049768.13 | 1.029670826 | 1.03E-18 | 5.30E-18 |
| *LRRC10B* | ENSG00000204950.3 | 1.030202681 | 2.01E-11 | 5.37E-11 |
| *KCNJ11* | ENSG00000187486.5 | 1.031245161 | 4.91E-33 | 1.10E-31 |
| *TMEM190* | ENSG00000160472.4 | 1.031988199 | 7.18E-08 | 1.41E-07 |
| *CABYR* | ENSG00000154040.19 | 1.032269115 | 6.30E-18 | 3.00E-17 |
| *SPHK1* | ENSG00000176170.12 | 1.033100274 | 1.12E-39 | 4.40E-38 |
| *SLC22A15* | ENSG00000163393.11 | 1.033476219 | 4.47E-32 | 9.33E-31 |
| *NOD2* | ENSG00000167207.10 | 1.033624571 | 2.93E-21 | 1.99E-20 |
| *NMB* | ENSG00000197696.8 | 1.035670942 | 8.44E-38 | 2.83E-36 |
| *GTF2IRD1* | ENSG00000006704.9 | 1.035714442 | 7.33E-47 | 4.74E-45 |
| *IL13RA2* | ENSG00000123496.6 | 1.037140863 | 2.62E-10 | 6.33E-10 |
| *MYLPF* | ENSG00000180209.10 | 1.037801087 | 6.16E-18 | 2.94E-17 |
| *FBXL13* | ENSG00000161040.15 | 1.037808863 | 3.98E-32 | 8.33E-31 |
| *COL9A1* | ENSG00000112280.14 | 1.038844451 | 1.40E-15 | 5.38E-15 |
| *EIF5A2* | ENSG00000163577.6 | 1.039450727 | 3.36E-20 | 2.01E-19 |
| *ZFP69B* | ENSG00000187801.13 | 1.040101325 | 5.60E-39 | 2.12E-37 |
| *MAP7D2* | ENSG00000184368.14 | 1.040482789 | 7.55E-20 | 4.37E-19 |
| *UBE3D* | ENSG00000118420.15 | 1.040555804 | 2.63E-35 | 7.25E-34 |
| *OSBPL3* | ENSG00000070882.11 | 1.041431609 | 8.24E-39 | 3.03E-37 |
| *LRRC15* | ENSG00000172061.8 | 1.042106978 | 8.05E-12 | 2.23E-11 |
| *ARID3A* | ENSG00000116017.9 | 1.044186778 | 2.98E-40 | 1.21E-38 |
| *CCDC74A* | ENSG00000163040.13 | 1.044807911 | 7.80E-26 | 8.59E-25 |
| *TIGD1* | ENSG00000221944.4 | 1.045535372 | 2.66E-51 | 2.06E-49 |
| *PPP1R1C* | ENSG00000150722.9 | 1.050372005 | 9.65E-17 | 4.14E-16 |
| *ANLN* | ENSG00000011426.9 | 1.050413725 | 3.78E-31 | 7.13E-30 |
| *C17orf53* | ENSG00000125319.13 | 1.050708599 | 2.95E-29 | 4.66E-28 |
| *IER5L* | ENSG00000188483.7 | 1.050769464 | 2.77E-31 | 5.32E-30 |
| *SLC39A10* | ENSG00000196950.12 | 1.053221327 | 1.87E-67 | 2.70E-65 |
| *GNG4* | ENSG00000168243.9 | 1.053499686 | 1.81E-24 | 1.72E-23 |
| *TBX18* | ENSG00000112837.15 | 1.054166892 | 9.95E-10 | 2.29E-09 |
| *AP1S3* | ENSG00000152056.15 | 1.054226452 | 1.45E-28 | 2.14E-27 |
| *CALCB* | ENSG00000175868.12 | 1.054503014 | 0.023557693 | 0.029285691 |
| *IQUB* | ENSG00000164675.9 | 1.055803646 | 3.09E-12 | 8.86E-12 |
| *LMOD3* | ENSG00000163380.14 | 1.057530594 | 2.24E-12 | 6.49E-12 |
| *IQCH* | ENSG00000103599.18 | 1.057759498 | 4.39E-38 | 1.50E-36 |
| *KIF18B* | ENSG00000186185.12 | 1.059662374 | 6.18E-27 | 7.71E-26 |
| *DLX2* | ENSG00000115844.9 | 1.05986345 | 0.000108063 | 0.000163581 |
| *SNAI1* | ENSG00000124216.3 | 1.059963252 | 4.49E-22 | 3.34E-21 |
| *ALPK3* | ENSG00000136383.6 | 1.060682247 | 8.52E-11 | 2.15E-10 |
| *KNDC1* | ENSG00000171798.16 | 1.060821362 | 5.33E-12 | 1.49E-11 |
| *FAM89A* | ENSG00000182118.5 | 1.061449169 | 9.67E-30 | 1.58E-28 |
| *TROAP* | ENSG00000135451.11 | 1.063981291 | 9.17E-29 | 1.37E-27 |
| *TEAD4* | ENSG00000197905.7 | 1.065653867 | 2.67E-34 | 6.76E-33 |
| *SKA3* | ENSG00000165480.14 | 1.066345249 | 4.69E-31 | 8.79E-30 |
| *CBX8* | ENSG00000141570.9 | 1.066685615 | 3.17E-50 | 2.36E-48 |
| *AKR1E2* | ENSG00000165568.16 | 1.067522266 | 1.49E-30 | 2.67E-29 |
| *GREB1* | ENSG00000196208.12 | 1.067536316 | 5.23E-19 | 2.76E-18 |
| *EDNRA* | ENSG00000151617.14 | 1.068339461 | 4.39E-27 | 5.57E-26 |
| *ITGA11* | ENSG00000137809.15 | 1.068980367 | 7.64E-31 | 1.41E-29 |
| *RDM1* | ENSG00000278023.3 | 1.069101174 | 3.53E-28 | 4.99E-27 |
| *SEC16B* | ENSG00000120341.17 | 1.069895756 | 2.40E-08 | 4.90E-08 |
| *ANGPT2* | ENSG00000091879.12 | 1.073805483 | 1.72E-26 | 2.05E-25 |
| *SLC9A5* | ENSG00000135740.15 | 1.07470704 | 2.33E-27 | 3.05E-26 |
| *C11orf16* | ENSG00000176029.12 | 1.076704039 | 7.44E-08 | 1.45E-07 |
| *POLQ* | ENSG00000051341.12 | 1.078739875 | 1.21E-23 | 1.05E-22 |
| *HOXB8* | ENSG00000120068.6 | 1.084834624 | 9.50E-34 | 2.29E-32 |
| *LZTS3* | ENSG00000088899.13 | 1.086591883 | 2.81E-52 | 2.31E-50 |
| *RPS6KL1* | ENSG00000198208.10 | 1.087444225 | 2.44E-36 | 7.38E-35 |
| *CTRL* | ENSG00000141086.16 | 1.08778925 | 3.49E-06 | 5.93E-06 |
| *PLEKHG4* | ENSG00000196155.11 | 1.088273023 | 6.62E-34 | 1.61E-32 |
| *KRT40* | ENSG00000204889.9 | 1.088582443 | 2.61E-08 | 5.32E-08 |
| *KLK2* | ENSG00000167751.11 | 1.089162756 | 0.000354034 | 0.000514082 |
| *BBOX1* | ENSG00000129151.7 | 1.090454008 | 0.001489816 | 0.002058563 |
| *TCF7* | ENSG00000081059.18 | 1.091673463 | 9.72E-33 | 2.13E-31 |
| *TTC9* | ENSG00000133985.2 | 1.092332963 | 1.65E-38 | 5.95E-37 |
| *FCN3* | ENSG00000142748.11 | 1.093715589 | 3.79E-07 | 7.00E-07 |
| *TMEM151A* | ENSG00000179292.4 | 1.094437496 | 3.48E-10 | 8.34E-10 |
| *TSPO2* | ENSG00000112212.10 | 1.094590119 | 1.04E-07 | 2.01E-07 |
| *SNTB1* | ENSG00000172164.12 | 1.096824261 | 2.95E-56 | 2.85E-54 |
| *PMAIP1* | ENSG00000141682.11 | 1.097254011 | 1.14E-34 | 2.99E-33 |
| *SYNPR* | ENSG00000163630.9 | 1.097540173 | 7.43E-05 | 0.00011382 |
| *MYH7B* | ENSG00000078814.14 | 1.097883673 | 6.01E-18 | 2.87E-17 |
| *C10orf67* | ENSG00000179133.10 | 1.098490039 | 9.76E-09 | 2.06E-08 |
| *BTBD11* | ENSG00000151136.13 | 1.09914115 | 7.05E-17 | 3.06E-16 |
| *PLEKHS1* | ENSG00000148735.13 | 1.100196691 | 1.71E-16 | 7.17E-16 |
| *PHLDA1* | ENSG00000139289.12 | 1.101372699 | 8.57E-28 | 1.17E-26 |
| *KLHL17* | ENSG00000187961.12 | 1.101444627 | 2.57E-34 | 6.53E-33 |
| *MCMDC2* | ENSG00000178460.16 | 1.101943794 | 2.17E-26 | 2.55E-25 |
| *FBXO43* | ENSG00000156509.12 | 1.102846207 | 4.86E-12 | 1.37E-11 |
| *DYDC2* | ENSG00000133665.11 | 1.103118042 | 0.000928611 | 0.00130634 |
| *PPP2R3B* | ENSG00000167393.15 | 1.103671048 | 3.16E-45 | 1.83E-43 |
| *GNB1L* | ENSG00000185838.12 | 1.105573172 | 5.59E-55 | 5.09E-53 |
| *HOXC9* | ENSG00000180806.4 | 1.105832622 | 3.88E-05 | 6.07E-05 |
| *FZD3* | ENSG00000104290.9 | 1.10626839 | 1.11E-30 | 2.02E-29 |
| *CDH16* | ENSG00000166589.11 | 1.109021738 | 1.53E-06 | 2.68E-06 |
| *GALNT9* | ENSG00000182870.11 | 1.109505474 | 1.34E-06 | 2.36E-06 |
| *CFAP43* | ENSG00000197748.11 | 1.110308632 | 1.35E-22 | 1.06E-21 |
| *MROH6* | ENSG00000204839.7 | 1.110326219 | 6.85E-39 | 2.55E-37 |
| *SLC22A3* | ENSG00000146477.5 | 1.112421287 | 1.31E-25 | 1.41E-24 |
| *ATP11A* | ENSG00000068650.17 | 1.113197587 | 1.08E-47 | 7.33E-46 |
| *WDR38* | ENSG00000136918.6 | 1.113534821 | 0.00022103 | 0.000325567 |
| *UHRF1* | ENSG00000276043.3 | 1.114274767 | 3.28E-29 | 5.16E-28 |
| *SLC7A5* | ENSG00000103257.7 | 1.114481367 | 1.47E-35 | 4.10E-34 |
| *DSCC1* | ENSG00000136982.5 | 1.115716249 | 1.02E-33 | 2.45E-32 |
| *HIST1H4E* | ENSG00000276966.1 | 1.117235211 | 5.80E-17 | 2.54E-16 |
| *ACTL8* | ENSG00000117148.7 | 1.119020012 | 6.52E-10 | 1.52E-09 |
| *YY2* | ENSG00000230797.2 | 1.122174894 | 5.20E-25 | 5.26E-24 |
| *DGKK* | ENSG00000274588.1 | 1.122230611 | 0.006629164 | 0.008671063 |
| *ANKFN1* | ENSG00000153930.9 | 1.122853688 | 6.17E-11 | 1.57E-10 |
| *LYPD6B* | ENSG00000150556.15 | 1.123456141 | 4.24E-17 | 1.88E-16 |
| *ATP2A1* | ENSG00000196296.12 | 1.12410347 | 4.73E-22 | 3.50E-21 |
| *SLC16A8* | ENSG00000100156.9 | 1.126126125 | 9.10E-14 | 2.97E-13 |
| *BDNF* | ENSG00000176697.17 | 1.126655321 | 1.55E-14 | 5.43E-14 |
| *MEIG1* | ENSG00000197889.8 | 1.127271504 | 4.34E-14 | 1.46E-13 |
| *CNBD2* | ENSG00000149646.11 | 1.127561038 | 2.11E-05 | 3.37E-05 |
| *OPCML* | ENSG00000183715.12 | 1.127879557 | 5.41E-09 | 1.16E-08 |
| *CACNA1E* | ENSG00000198216.9 | 1.13055586 | 7.69E-05 | 0.000117698 |
| *OVGP1* | ENSG00000085465.12 | 1.131545341 | 3.79E-33 | 8.63E-32 |
| *PILRB* | ENSG00000121716.17 | 1.132892111 | 1.37E-14 | 4.82E-14 |
| *PDE10A* | ENSG00000112541.12 | 1.13300671 | 1.02E-14 | 3.63E-14 |
| *BNIPL* | ENSG00000163141.17 | 1.134831386 | 4.95E-23 | 4.05E-22 |
| *F2RL2* | ENSG00000164220.6 | 1.134881066 | 4.11E-21 | 2.75E-20 |
| *LIME1* | ENSG00000203896.8 | 1.136246832 | 8.43E-30 | 1.39E-28 |
| *DIAPH3* | ENSG00000139734.16 | 1.1369261 | 8.26E-29 | 1.24E-27 |
| *GLYATL2* | ENSG00000156689.5 | 1.137290872 | 4.11E-06 | 6.95E-06 |
| *KRT36* | ENSG00000126337.12 | 1.137880247 | 4.22E-08 | 8.44E-08 |
| *REG1A* | ENSG00000115386.5 | 1.141254999 | 7.46E-06 | 1.24E-05 |
| *LRRC73* | ENSG00000204052.4 | 1.141855583 | 1.20E-17 | 5.58E-17 |
| *LGR5* | ENSG00000139292.11 | 1.143946899 | 1.39E-38 | 5.03E-37 |
| *KCNS1* | ENSG00000124134.7 | 1.14500452 | 7.89E-08 | 1.54E-07 |
| *SLC6A1* | ENSG00000157103.9 | 1.145756161 | 1.58E-17 | 7.27E-17 |
| *MOV10L1* | ENSG00000073146.14 | 1.145964382 | 3.30E-08 | 6.65E-08 |
| *CTRC* | ENSG00000162438.10 | 1.147158237 | 0.000693378 | 0.000985189 |
| *BICD1* | ENSG00000151746.12 | 1.147224225 | 6.11E-31 | 1.14E-29 |
| *LYPD3* | ENSG00000124466.8 | 1.147797869 | 1.17E-30 | 2.11E-29 |
| *TNFSF15* | ENSG00000181634.7 | 1.150242813 | 1.80E-15 | 6.85E-15 |
| *CAPNS2* | ENSG00000256812.1 | 1.15034073 | 1.91E-08 | 3.93E-08 |
| *CENPI* | ENSG00000102384.12 | 1.152237174 | 2.02E-32 | 4.32E-31 |
| *GHRHR* | ENSG00000106128.17 | 1.152398548 | 3.73E-05 | 5.85E-05 |
| *ACMSD* | ENSG00000153086.12 | 1.152550118 | 0.000426806 | 0.000616223 |
| *MC1R* | ENSG00000258839.2 | 1.152559416 | 2.78E-28 | 3.98E-27 |
| *HIST1H2BM* | ENSG00000273703.1 | 1.154315786 | 0.000761529 | 0.001079542 |
| *SRMS* | ENSG00000125508.3 | 1.157670974 | 2.88E-26 | 3.33E-25 |
| *ZDHHC11* | ENSG00000188818.11 | 1.157982692 | 1.43E-10 | 3.53E-10 |
| *SPATA6L* | ENSG00000106686.15 | 1.158093795 | 2.51E-21 | 1.72E-20 |
| *RPGRIP1L* | ENSG00000103494.11 | 1.162513936 | 7.12E-38 | 2.40E-36 |
| *ELFN1* | ENSG00000225968.6 | 1.163081152 | 1.60E-18 | 8.08E-18 |
| *TICRR* | ENSG00000140534.12 | 1.163914901 | 1.25E-28 | 1.85E-27 |
| *OVOL3* | ENSG00000105261.6 | 1.164234489 | 1.60E-10 | 3.94E-10 |
| *PODNL1* | ENSG00000132000.10 | 1.164272532 | 2.29E-33 | 5.33E-32 |
| *INHBB* | ENSG00000163083.5 | 1.16597868 | 1.70E-21 | 1.18E-20 |
| *RPE65* | ENSG00000116745.6 | 1.167904154 | 0.001384848 | 0.001919697 |
| *AZGP1* | ENSG00000160862.11 | 1.175595677 | 6.76E-39 | 2.53E-37 |
| *GNMT* | ENSG00000124713.5 | 1.175840561 | 1.01E-18 | 5.20E-18 |
| *ANKRD30B* | ENSG00000180777.12 | 1.176022853 | 0.00021907 | 0.000322803 |
| *PMFBP1* | ENSG00000118557.14 | 1.177471049 | 5.35E-23 | 4.36E-22 |
| *ICAM5* | ENSG00000105376.4 | 1.181730788 | 5.53E-16 | 2.22E-15 |
| *SLC17A9* | ENSG00000101194.16 | 1.183522088 | 1.46E-22 | 1.14E-21 |
| *GPR84* | ENSG00000139572.3 | 1.183536396 | 6.32E-12 | 1.76E-11 |
| *RGS16* | ENSG00000143333.6 | 1.18392681 | 3.38E-25 | 3.49E-24 |
| *TEX22* | ENSG00000226174.5 | 1.185089543 | 8.78E-27 | 1.08E-25 |
| *CAMK2N2* | ENSG00000163888.3 | 1.185217234 | 1.91E-12 | 5.57E-12 |
| *ACRV1* | ENSG00000134940.12 | 1.18630753 | 1.49E-23 | 1.28E-22 |
| *SMOX* | ENSG00000088826.16 | 1.19019945 | 9.19E-60 | 1.03E-57 |
| *HECW2* | ENSG00000138411.9 | 1.191910573 | 1.02E-30 | 1.87E-29 |
| *NME2* | ENSG00000011052.20 | 1.193210074 | 1.71E-26 | 2.04E-25 |
| *ABHD1* | ENSG00000143994.12 | 1.193499978 | 6.64E-20 | 3.87E-19 |
| *REG1B* | ENSG00000172023.6 | 1.194543739 | 0.001159185 | 0.001617171 |
| *TMEM178B* | ENSG00000261115.4 | 1.195205212 | 3.42E-14 | 1.16E-13 |
| *CAGE1* | ENSG00000164304.14 | 1.196490466 | 1.63E-09 | 3.68E-09 |
| *GRM1* | ENSG00000152822.12 | 1.197095318 | 0.001525484 | 0.002105966 |
| *R3HDML* | ENSG00000101074.3 | 1.197917591 | 7.45E-22 | 5.39E-21 |
| *B3GNT4* | ENSG00000176383.8 | 1.197972828 | 1.70E-27 | 2.25E-26 |
| *SLC27A5* | ENSG00000083807.8 | 1.198195803 | 5.55E-45 | 3.14E-43 |
| *SPP1* | ENSG00000118785.12 | 1.198976043 | 3.23E-24 | 3.00E-23 |
| *SYCE3* | ENSG00000217442.3 | 1.199969794 | 1.13E-19 | 6.42E-19 |
| *HSF2BP* | ENSG00000160207.7 | 1.200656906 | 1.74E-32 | 3.73E-31 |
| *OCA2* | ENSG00000104044.14 | 1.201192471 | 3.77E-10 | 9.00E-10 |
| *WFDC13* | ENSG00000168634.4 | 1.202140091 | 8.76E-09 | 1.85E-08 |
| *TNFSF11* | ENSG00000120659.13 | 1.202189589 | 1.46E-19 | 8.20E-19 |
| *GUCY2D* | ENSG00000132518.6 | 1.202987086 | 2.21E-09 | 4.93E-09 |
| *TMEM255B* | ENSG00000184497.11 | 1.203278479 | 3.90E-32 | 8.17E-31 |
| *ANKRD31* | ENSG00000145700.8 | 1.20385029 | 4.14E-13 | 1.28E-12 |
| *SSC4D* | ENSG00000146700.8 | 1.205156838 | 2.79E-32 | 5.92E-31 |
| *PIF1* | ENSG00000140451.11 | 1.207480924 | 4.21E-26 | 4.77E-25 |
| *TDO2* | ENSG00000151790.7 | 1.209589916 | 6.03E-20 | 3.53E-19 |
| *RNASE7* | ENSG00000165799.4 | 1.210053266 | 1.52E-11 | 4.10E-11 |
| *LDLRAD3* | ENSG00000179241.11 | 1.211490332 | 9.17E-43 | 4.43E-41 |
| *ADAMTS2* | ENSG00000087116.12 | 1.211895944 | 3.98E-34 | 9.91E-33 |
| *CATSPER1* | ENSG00000175294.5 | 1.2141616 | 1.18E-17 | 5.49E-17 |
| *DNMT3B* | ENSG00000088305.17 | 1.214323153 | 2.74E-55 | 2.54E-53 |
| *GPR143* | ENSG00000101850.11 | 1.214702254 | 7.14E-27 | 8.86E-26 |
| *MCM10* | ENSG00000065328.15 | 1.220301265 | 5.16E-27 | 6.50E-26 |
| *MYT1* | ENSG00000196132.10 | 1.221104882 | 1.67E-09 | 3.77E-09 |
| *ZNF239* | ENSG00000196793.12 | 1.222784779 | 2.03E-30 | 3.57E-29 |
| *SLC5A2* | ENSG00000140675.11 | 1.22305576 | 2.41E-13 | 7.59E-13 |
| *C2CD4B* | ENSG00000205502.3 | 1.22352916 | 4.50E-27 | 5.70E-26 |
| *POLR3G* | ENSG00000113356.9 | 1.224115915 | 3.62E-37 | 1.16E-35 |
| *PLA2G4B* | ENSG00000243708.7 | 1.224435958 | 6.32E-16 | 2.52E-15 |
| *S1PR5* | ENSG00000180739.13 | 1.228631185 | 6.52E-23 | 5.27E-22 |
| *STX1A* | ENSG00000106089.10 | 1.229225659 | 6.95E-27 | 8.64E-26 |
| *SHH* | ENSG00000164690.6 | 1.22930572 | 8.27E-33 | 1.83E-31 |
| *ODF3L2* | ENSG00000181781.8 | 1.229742974 | 1.86E-16 | 7.78E-16 |
| *RPA4* | ENSG00000204086.4 | 1.230155201 | 3.73E-17 | 1.66E-16 |
| *TNFRSF10C* | ENSG00000173535.12 | 1.23091794 | 2.32E-27 | 3.04E-26 |
| *JMJD7* | ENSG00000243789.9 | 1.232009039 | 3.55E-12 | 1.01E-11 |
| *B3GNTL1* | ENSG00000175711.7 | 1.232497407 | 1.35E-51 | 1.08E-49 |
| *ZBED2* | ENSG00000177494.5 | 1.233781927 | 2.57E-10 | 6.22E-10 |
| *AGBL4* | ENSG00000186094.15 | 1.234219491 | 6.22E-14 | 2.06E-13 |
| *MPP3* | ENSG00000161647.17 | 1.236759585 | 3.06E-24 | 2.84E-23 |
| *FOXS1* | ENSG00000179772.7 | 1.237040143 | 1.91E-23 | 1.62E-22 |
| *SLC9A7* | ENSG00000065923.8 | 1.240731849 | 2.16E-37 | 6.97E-36 |
| *S100A3* | ENSG00000188015.8 | 1.240736721 | 1.57E-23 | 1.34E-22 |
| *KIAA0895* | ENSG00000164542.11 | 1.242448954 | 4.59E-30 | 7.81E-29 |
| *DRC7* | ENSG00000159625.13 | 1.243056507 | 0.00044371 | 0.000640091 |
| *MTHFD1L* | ENSG00000120254.14 | 1.243667792 | 2.13E-36 | 6.46E-35 |
| *MME* | ENSG00000196549.9 | 1.24414 | 7.58E-05 | 0.000116072 |
| *PPFIA4* | ENSG00000143847.14 | 1.246741771 | 5.68E-13 | 1.73E-12 |
| *APOC2* | ENSG00000234906.7 | 1.249271654 | 2.37E-12 | 6.85E-12 |
| *SUN3* | ENSG00000164744.11 | 1.251034544 | 5.31E-10 | 1.25E-09 |
| *CD3EAP* | ENSG00000117877.9 | 1.25106109 | 7.09E-42 | 3.26E-40 |
| *C5orf34* | ENSG00000172244.7 | 1.25276625 | 6.56E-39 | 2.46E-37 |
| *REN* | ENSG00000143839.13 | 1.253137107 | 9.06E-12 | 2.50E-11 |
| *CNIH3* | ENSG00000143786.6 | 1.255084425 | 2.56E-44 | 1.38E-42 |
| *SCLY* | ENSG00000132330.15 | 1.255797547 | 1.27E-45 | 7.53E-44 |
| *MACC1* | ENSG00000183742.11 | 1.259062425 | 9.07E-30 | 1.49E-28 |
| *MST1* | ENSG00000173531.14 | 1.260508888 | 1.76E-17 | 8.06E-17 |
| *DPPA4* | ENSG00000121570.11 | 1.261126128 | 0.000766878 | 0.001086727 |
| *UNC5CL* | ENSG00000124602.8 | 1.261539461 | 1.36E-19 | 7.67E-19 |
| *MORN3* | ENSG00000139714.11 | 1.261812353 | 1.09E-14 | 3.87E-14 |
| *ASCL2* | ENSG00000183734.4 | 1.261911009 | 1.16E-50 | 8.84E-49 |
| *NFE2* | ENSG00000123405.12 | 1.26197214 | 1.59E-11 | 4.28E-11 |
| *RHEBL1* | ENSG00000167550.9 | 1.262338448 | 1.17E-30 | 2.11E-29 |
| *ERCC6L* | ENSG00000186871.6 | 1.262360178 | 6.57E-33 | 1.46E-31 |
| *TRPV4* | ENSG00000111199.9 | 1.262879292 | 6.75E-29 | 1.02E-27 |
| *DCLK3* | ENSG00000163673.6 | 1.263024581 | 1.59E-20 | 9.84E-20 |
| *MFAP2* | ENSG00000117122.12 | 1.263167763 | 5.18E-42 | 2.41E-40 |
| *DGAT2* | ENSG00000062282.13 | 1.263703707 | 2.26E-20 | 1.38E-19 |
| *CDH4* | ENSG00000179242.14 | 1.264218878 | 2.65E-13 | 8.33E-13 |
| *SBK1* | ENSG00000188322.4 | 1.265214646 | 2.04E-33 | 4.80E-32 |
| *SLC30A2* | ENSG00000158014.13 | 1.266026173 | 1.07E-06 | 1.90E-06 |
| *SPNS3* | ENSG00000182557.6 | 1.268756018 | 3.24E-28 | 4.60E-27 |
| *HCAR2* | ENSG00000182782.7 | 1.268868993 | 5.37E-10 | 1.26E-09 |
| *INHBE* | ENSG00000139269.2 | 1.269544397 | 3.36E-32 | 7.10E-31 |
| *SHROOM4* | ENSG00000158352.14 | 1.26973974 | 2.31E-47 | 1.52E-45 |
| *FAM83C* | ENSG00000125998.7 | 1.270517735 | 3.10E-06 | 5.30E-06 |
| *OR2B6* | ENSG00000124657.1 | 1.270616412 | 1.22E-10 | 3.03E-10 |
| *CLCN4* | ENSG00000073464.10 | 1.27175705 | 9.01E-47 | 5.78E-45 |
| *DRC1* | ENSG00000157856.9 | 1.272017229 | 1.07E-06 | 1.90E-06 |
| *RANBP17* | ENSG00000204764.11 | 1.273666139 | 3.56E-46 | 2.23E-44 |
| *PPM1H* | ENSG00000111110.10 | 1.273894407 | 5.65E-65 | 7.47E-63 |
| *TRIP13* | ENSG00000071539.12 | 1.273923866 | 7.37E-34 | 1.79E-32 |
| *ADAMTS18* | ENSG00000140873.14 | 1.276068889 | 3.65E-09 | 7.97E-09 |
| *USH2A* | ENSG00000042781.11 | 1.276336538 | 6.00E-09 | 1.29E-08 |
| *RDH12* | ENSG00000139988.8 | 1.276713746 | 1.42E-23 | 1.22E-22 |
| *SYNGR4* | ENSG00000105467.7 | 1.277557533 | 8.96E-12 | 2.47E-11 |
| *CBS* | ENSG00000160200.16 | 1.277844126 | 8.02E-09 | 1.70E-08 |
| *FCAR* | ENSG00000186431.17 | 1.278201497 | 1.82E-08 | 3.75E-08 |
| *GRM8* | ENSG00000179603.16 | 1.278605806 | 3.98E-29 | 6.17E-28 |
| *KLF1* | ENSG00000105610.4 | 1.278986587 | 1.37E-16 | 5.79E-16 |
| *ZC3HAV1L* | ENSG00000146858.7 | 1.280083665 | 2.12E-45 | 1.24E-43 |
| *TRAPPC5* | ENSG00000181029.8 | 1.282797547 | 4.27E-26 | 4.83E-25 |
| *ORC6* | ENSG00000091651.7 | 1.285479011 | 2.72E-33 | 6.30E-32 |
| *SLC4A8* | ENSG00000050438.15 | 1.286872171 | 2.57E-27 | 3.34E-26 |
| *TTC26* | ENSG00000105948.12 | 1.287037474 | 1.36E-54 | 1.22E-52 |
| *PROZ* | ENSG00000126231.12 | 1.287256606 | 1.97E-13 | 6.25E-13 |
| *TRIM7* | ENSG00000146054.16 | 1.288085658 | 2.71E-20 | 1.64E-19 |
| *ASB9* | ENSG00000102048.14 | 1.289272389 | 1.08E-52 | 9.08E-51 |
| *HIST1H2BE* | ENSG00000274290.1 | 1.289830117 | 1.10E-08 | 2.31E-08 |
| *ASIC1* | ENSG00000110881.10 | 1.290867031 | 1.01E-19 | 5.77E-19 |
| *DCDC2* | ENSG00000146038.10 | 1.294246217 | 5.62E-16 | 2.26E-15 |
| *VSIG1* | ENSG00000101842.12 | 1.297294043 | 1.74E-05 | 2.80E-05 |
| *LZTS1* | ENSG00000061337.14 | 1.299406617 | 1.74E-31 | 3.41E-30 |
| *CAPN12* | ENSG00000182472.7 | 1.304513041 | 5.93E-32 | 1.22E-30 |
| *NEBL* | ENSG00000078114.17 | 1.30682673 | 1.38E-37 | 4.56E-36 |
| *LRRC46* | ENSG00000141294.8 | 1.309417233 | 3.57E-39 | 1.37E-37 |
| *NR0B2* | ENSG00000131910.4 | 1.310810214 | 3.73E-10 | 8.91E-10 |
| *STC1* | ENSG00000159167.10 | 1.311193936 | 2.25E-19 | 1.24E-18 |
| *CCDC113* | ENSG00000103021.8 | 1.311920009 | 1.96E-63 | 2.43E-61 |
| *CHSY3* | ENSG00000198108.3 | 1.312536288 | 9.24E-37 | 2.89E-35 |
| *F5* | ENSG00000198734.9 | 1.315804519 | 8.70E-19 | 4.51E-18 |
| *SPRED3* | ENSG00000188766.11 | 1.316868455 | 1.02E-20 | 6.47E-20 |
| *KRT15* | ENSG00000171346.12 | 1.316902323 | 9.33E-25 | 9.15E-24 |
| *COL27A1* | ENSG00000196739.13 | 1.317354098 | 8.55E-28 | 1.17E-26 |
| *RLN2* | ENSG00000107014.8 | 1.319451381 | 9.48E-19 | 4.90E-18 |
| *PHACTR3* | ENSG00000087495.15 | 1.319784921 | 7.80E-22 | 5.64E-21 |
| *CDRT1* | ENSG00000241322.7 | 1.320123834 | 1.26E-14 | 4.45E-14 |
| *CEP72* | ENSG00000112877.7 | 1.32113506 | 1.20E-60 | 1.40E-58 |
| *AMIGO3* | ENSG00000176020.8 | 1.321843602 | 2.07E-06 | 3.59E-06 |
| *PPM1N* | ENSG00000213889.9 | 1.323737906 | 5.14E-32 | 1.07E-30 |
| *TCHH* | ENSG00000159450.11 | 1.324106915 | 3.07E-05 | 4.85E-05 |
| *DNAH10* | ENSG00000197653.13 | 1.324256476 | 6.66E-14 | 2.20E-13 |
| *SLC6A6* | ENSG00000131389.15 | 1.32704059 | 3.02E-38 | 1.05E-36 |
| *CYP19A1* | ENSG00000137869.12 | 1.328947037 | 1.15E-14 | 4.08E-14 |
| *GPC2* | ENSG00000213420.6 | 1.329817423 | 1.97E-26 | 2.33E-25 |
| *NPM2* | ENSG00000158806.12 | 1.329928782 | 6.57E-40 | 2.62E-38 |
| *EPO* | ENSG00000130427.2 | 1.337498049 | 1.65E-09 | 3.73E-09 |
| *RAB36* | ENSG00000100228.11 | 1.340250636 | 1.30E-30 | 2.34E-29 |
| *GABRE* | ENSG00000102287.15 | 1.340455883 | 8.01E-41 | 3.38E-39 |
| *SPATA17* | ENSG00000162814.9 | 1.340822173 | 3.17E-36 | 9.54E-35 |
| *CPB2* | ENSG00000080618.12 | 1.341502058 | 0.02501327 | 0.031002984 |
| *GABRA3* | ENSG00000011677.11 | 1.342036111 | 5.69E-06 | 9.50E-06 |
| *EME1* | ENSG00000154920.13 | 1.345621597 | 1.62E-40 | 6.70E-39 |
| *CSF3* | ENSG00000108342.11 | 1.346558696 | 5.05E-07 | 9.23E-07 |
| *FRMD5* | ENSG00000171877.18 | 1.346936392 | 1.56E-46 | 9.97E-45 |
| *CXCL3* | ENSG00000163734.4 | 1.349580193 | 1.33E-22 | 1.04E-21 |
| *OTUB2* | ENSG00000089723.8 | 1.350125187 | 4.76E-57 | 4.81E-55 |
| *HPN* | ENSG00000105707.12 | 1.353904761 | 1.60E-06 | 2.80E-06 |
| *TWIST1* | ENSG00000122691.11 | 1.35442867 | 1.21E-27 | 1.63E-26 |
| *UMODL1* | ENSG00000177398.17 | 1.354945724 | 1.12E-15 | 4.34E-15 |
| *SRPX2* | ENSG00000102359.5 | 1.355634525 | 1.07E-36 | 3.31E-35 |
| *VASH2* | ENSG00000143494.14 | 1.355871651 | 2.62E-40 | 1.07E-38 |
| *PCP2* | ENSG00000174788.8 | 1.358629307 | 2.79E-19 | 1.52E-18 |
| *MPP6* | ENSG00000105926.14 | 1.358641394 | 1.34E-21 | 9.43E-21 |
| *MSH4* | ENSG00000057468.6 | 1.359476062 | 7.51E-08 | 1.47E-07 |
| *RBP3* | ENSG00000265203.1 | 1.362590651 | 0.036643838 | 0.044635237 |
| *FAM53A* | ENSG00000174137.11 | 1.365732128 | 8.13E-43 | 3.94E-41 |
| *TERT* | ENSG00000164362.17 | 1.368686393 | 9.63E-24 | 8.47E-23 |
| *KIF14* | ENSG00000118193.10 | 1.369025256 | 3.11E-34 | 7.83E-33 |
| *OXT* | ENSG00000101405.3 | 1.37277905 | 4.38E-11 | 1.13E-10 |
| *SH2D4B* | ENSG00000178217.12 | 1.37301622 | 1.40E-10 | 3.46E-10 |
| *CCDC168* | ENSG00000175820.3 | 1.373437503 | 5.46E-11 | 1.40E-10 |
| *RPEL1* | ENSG00000235376.5 | 1.374733792 | 6.12E-11 | 1.56E-10 |
| *FBXO41* | ENSG00000163013.10 | 1.376840882 | 6.81E-59 | 7.31E-57 |
| *TMEM145* | ENSG00000167619.10 | 1.37768727 | 3.54E-17 | 1.58E-16 |
| *MYCN* | ENSG00000134323.10 | 1.379495503 | 2.27E-32 | 4.85E-31 |
| *SLC2A12* | ENSG00000146411.5 | 1.38743079 | 1.53E-16 | 6.44E-16 |
| *GABRA4* | ENSG00000109158.9 | 1.389009457 | 1.17E-05 | 1.91E-05 |
| *LEF1* | ENSG00000138795.8 | 1.389824869 | 1.55E-33 | 3.67E-32 |
| *TNS4* | ENSG00000131746.11 | 1.39177253 | 1.44E-35 | 4.03E-34 |
| *RTN4RL2* | ENSG00000186907.6 | 1.392574348 | 6.30E-29 | 9.58E-28 |
| *S100A5* | ENSG00000196420.6 | 1.3927963 | 5.26E-30 | 8.90E-29 |
| *C9orf43* | ENSG00000157653.10 | 1.396781767 | 4.38E-43 | 2.15E-41 |
| *B4GALNT4* | ENSG00000182272.10 | 1.397421065 | 3.92E-33 | 8.92E-32 |
| *KRT5* | ENSG00000186081.10 | 1.39760924 | 0.001247347 | 0.001734844 |
| *WDR62* | ENSG00000075702.15 | 1.399794262 | 7.10E-35 | 1.88E-33 |
| *FZD10* | ENSG00000111432.4 | 1.401529383 | 2.94E-16 | 1.21E-15 |
| *PRR19* | ENSG00000188368.8 | 1.403362494 | 8.92E-78 | 1.84E-75 |
| *MYOM3* | ENSG00000142661.17 | 1.404891197 | 1.57E-19 | 8.79E-19 |
| *SLC25A2* | ENSG00000120329.6 | 1.413383209 | 2.85E-09 | 6.29E-09 |
| *DIO2* | ENSG00000211448.10 | 1.416900257 | 5.02E-40 | 2.03E-38 |
| *PDZD7* | ENSG00000186862.16 | 1.418953924 | 0.000168132 | 0.000250128 |
| *SFRP4* | ENSG00000106483.10 | 1.419898171 | 2.18E-23 | 1.84E-22 |
| *VSNL1* | ENSG00000163032.10 | 1.422372787 | 5.48E-46 | 3.34E-44 |
| *PCSK9* | ENSG00000169174.10 | 1.424882841 | 8.59E-21 | 5.51E-20 |
| *ASIP* | ENSG00000101440.8 | 1.427404456 | 1.67E-19 | 9.32E-19 |
| *C9* | ENSG00000113600.9 | 1.427424749 | 1.57E-11 | 4.23E-11 |
| *FGF4* | ENSG00000075388.3 | 1.427649024 | 2.27E-07 | 4.27E-07 |
| *ASPHD1* | ENSG00000174939.9 | 1.427920498 | 7.04E-72 | 1.16E-69 |
| *RDH8* | ENSG00000080511.3 | 1.432104916 | 0.002963128 | 0.003994747 |
| *COL9A3* | ENSG00000092758.14 | 1.432567355 | 1.08E-45 | 6.42E-44 |
| *OSM* | ENSG00000099985.3 | 1.433463893 | 4.14E-14 | 1.40E-13 |
| *CPA5* | ENSG00000158525.14 | 1.438038649 | 0.000119277 | 0.000179817 |
| *DCAF4L1* | ENSG00000182308.6 | 1.440303745 | 3.70E-27 | 4.75E-26 |
| *TKTL1* | ENSG00000007350.15 | 1.440708712 | 0.001965741 | 0.002693077 |
| *GPR19* | ENSG00000183150.6 | 1.443521518 | 1.54E-40 | 6.39E-39 |
| *CCL26* | ENSG00000006606.7 | 1.444534737 | 1.07E-25 | 1.16E-24 |
| *C9orf116* | ENSG00000160345.11 | 1.446184668 | 1.00E-54 | 8.99E-53 |
| *TYRO3* | ENSG00000092445.10 | 1.447948179 | 3.05E-35 | 8.36E-34 |
| *SLC22A31* | ENSG00000259803.5 | 1.448434544 | 2.41E-09 | 5.36E-09 |
| *MTBP* | ENSG00000172167.6 | 1.449412019 | 3.87E-52 | 3.17E-50 |
| *HECW1* | ENSG00000002746.13 | 1.450387708 | 1.62E-28 | 2.38E-27 |
| *TRPM8* | ENSG00000144481.15 | 1.451130538 | 0.000127745 | 0.000192189 |
| *ZNF114* | ENSG00000178150.7 | 1.451325402 | 2.66E-19 | 1.45E-18 |
| *SMTNL2* | ENSG00000188176.10 | 1.451747398 | 4.25E-19 | 2.27E-18 |
| *ECEL1* | ENSG00000171551.10 | 1.456631568 | 1.44E-11 | 3.89E-11 |
| *FGF18* | ENSG00000156427.7 | 1.458099687 | 6.30E-24 | 5.64E-23 |
| *CCDC114* | ENSG00000105479.14 | 1.461677191 | 1.90E-21 | 1.32E-20 |
| *LRRC74A* | ENSG00000100565.14 | 1.463770287 | 8.31E-10 | 1.92E-09 |
| *FAM166A* | ENSG00000188163.7 | 1.464551783 | 4.64E-10 | 1.10E-09 |
| *NMU* | ENSG00000109255.10 | 1.467624353 | 4.56E-47 | 2.98E-45 |
| *RETN* | ENSG00000104918.6 | 1.470775861 | 1.94E-11 | 5.19E-11 |
| *DBNDD1* | ENSG00000003249.12 | 1.473830647 | 1.47E-51 | 1.17E-49 |
| *DDIAS* | ENSG00000165490.11 | 1.477493346 | 6.13E-36 | 1.79E-34 |
| *FAM72A* | ENSG00000196550.9 | 1.479879939 | 6.04E-44 | 3.13E-42 |
| *ANKRD13B* | ENSG00000198720.11 | 1.482301321 | 2.94E-43 | 1.47E-41 |
| *E2F7* | ENSG00000165891.14 | 1.484585398 | 1.12E-39 | 4.40E-38 |
| *EREG* | ENSG00000124882.3 | 1.487974158 | 5.92E-44 | 3.09E-42 |
| *TRPV6* | ENSG00000165125.16 | 1.488692649 | 2.04E-07 | 3.85E-07 |
| *SLC34A1* | ENSG00000131183.9 | 1.491125313 | 3.04E-14 | 1.04E-13 |
| *AANAT* | ENSG00000129673.8 | 1.491985057 | 1.40E-14 | 4.92E-14 |
| *TMEM132A* | ENSG00000006118.13 | 1.493251691 | 4.93E-61 | 5.78E-59 |
| *PABPC3* | ENSG00000151846.8 | 1.494993361 | 3.72E-27 | 4.77E-26 |
| *AMBP* | ENSG00000106927.10 | 1.495082302 | 1.79E-17 | 8.19E-17 |
| *ARNTL2* | ENSG00000029153.13 | 1.498639147 | 4.25E-50 | 3.15E-48 |
| *PBX4* | ENSG00000105717.12 | 1.499494012 | 2.53E-42 | 1.20E-40 |
| *ODAM* | ENSG00000109205.15 | 1.499868209 | 1.89E-11 | 5.06E-11 |
| *SRCIN1* | ENSG00000277363.3 | 1.502042402 | 1.03E-42 | 4.96E-41 |
| *P4HA3* | ENSG00000149380.10 | 1.503171062 | 2.57E-24 | 2.41E-23 |
| *GUCA1A* | ENSG00000048545.12 | 1.504333692 | 2.51E-14 | 8.63E-14 |
| *FOXB1* | ENSG00000171956.6 | 1.504463649 | 0.000109343 | 0.000165453 |
| *GPR78* | ENSG00000155269.10 | 1.504821389 | 1.68E-13 | 5.36E-13 |
| *BRCA2* | ENSG00000139618.13 | 1.505051445 | 6.10E-46 | 3.69E-44 |
| *KIF25* | ENSG00000125337.15 | 1.507994676 | 1.53E-21 | 1.07E-20 |
| *FABP7* | ENSG00000164434.10 | 1.509599863 | 6.83E-06 | 1.13E-05 |
| *ATAD3C* | ENSG00000215915.8 | 1.510367009 | 1.98E-37 | 6.45E-36 |
| *CYP24A1* | ENSG00000019186.8 | 1.512424763 | 0.000100578 | 0.000152594 |
| *FOXD4* | ENSG00000170122.5 | 1.516556874 | 4.88E-22 | 3.61E-21 |
| *GABRR1* | ENSG00000146276.10 | 1.518056844 | 3.68E-07 | 6.80E-07 |
| *EPHX3* | ENSG00000105131.6 | 1.519914957 | 1.18E-22 | 9.30E-22 |
| *LCN12* | ENSG00000184925.10 | 1.52212533 | 1.63E-25 | 1.74E-24 |
| *DPF1* | ENSG00000011332.18 | 1.52442778 | 1.08E-32 | 2.36E-31 |
| *HOXC6* | ENSG00000197757.7 | 1.524869379 | 1.17E-09 | 2.67E-09 |
| *RIIAD1* | ENSG00000178796.11 | 1.526034058 | 2.20E-21 | 1.51E-20 |
| *RELL2* | ENSG00000164620.7 | 1.526106099 | 2.37E-56 | 2.32E-54 |
| *CXCL8* | ENSG00000169429.9 | 1.526443374 | 1.07E-18 | 5.48E-18 |
| *RSPH14* | ENSG00000100218.10 | 1.531219675 | 8.86E-42 | 4.03E-40 |
| *ATP6V1C2* | ENSG00000143882.8 | 1.531606316 | 4.74E-55 | 4.36E-53 |
| *IL13* | ENSG00000169194.8 | 1.5322243 | 1.23E-07 | 2.36E-07 |
| *TAF1L* | ENSG00000122728.6 | 1.534053344 | 5.41E-09 | 1.16E-08 |
| *PPP2R2C* | ENSG00000074211.12 | 1.53877134 | 2.75E-20 | 1.66E-19 |
| *MYO3B* | ENSG00000071909.17 | 1.54319802 | 3.71E-13 | 1.15E-12 |
| *NDP* | ENSG00000124479.8 | 1.543531626 | 9.80E-21 | 6.24E-20 |
| *CLDN10* | ENSG00000134873.8 | 1.544001525 | 2.69E-06 | 4.61E-06 |
| *COL6A6* | ENSG00000206384.9 | 1.544718587 | 2.45E-08 | 5.00E-08 |
| *GADL1* | ENSG00000144644.13 | 1.546020894 | 3.41E-06 | 5.80E-06 |
| *REM2* | ENSG00000139890.8 | 1.548380083 | 5.94E-57 | 5.97E-55 |
| *DCT* | ENSG00000080166.14 | 1.549424546 | 2.05E-05 | 3.28E-05 |
| *HIST1H2BO* | ENSG00000274641.1 | 1.551723303 | 8.13E-11 | 2.05E-10 |
| *SPEF1* | ENSG00000101222.11 | 1.552452773 | 5.99E-17 | 2.61E-16 |
| *AQP9* | ENSG00000103569.8 | 1.552745189 | 3.00E-13 | 9.36E-13 |
| *CADPS* | ENSG00000163618.16 | 1.552850634 | 4.68E-59 | 5.13E-57 |
| *CDH15* | ENSG00000129910.6 | 1.557565443 | 5.78E-18 | 2.77E-17 |
| *XRCC2* | ENSG00000196584.2 | 1.557713235 | 1.61E-41 | 7.20E-40 |
| *CECR2* | ENSG00000099954.17 | 1.557736878 | 3.30E-15 | 1.23E-14 |
| *CYP27B1* | ENSG00000111012.8 | 1.559307489 | 3.94E-53 | 3.35E-51 |
| *BTN1A1* | ENSG00000124557.11 | 1.560213835 | 1.30E-12 | 3.85E-12 |
| *FBXO39* | ENSG00000177294.6 | 1.564265941 | 2.30E-16 | 9.55E-16 |
| *MYBPC3* | ENSG00000134571.9 | 1.564897364 | 3.51E-08 | 7.06E-08 |
| *CYP4X1* | ENSG00000186377.7 | 1.566053497 | 6.84E-54 | 5.98E-52 |
| *ANP32D* | ENSG00000139223.2 | 1.567695211 | 3.46E-09 | 7.57E-09 |
| *ZNF469* | ENSG00000225614.2 | 1.571772851 | 2.10E-47 | 1.40E-45 |
| *GREB1L* | ENSG00000141449.13 | 1.573817306 | 1.00E-09 | 2.30E-09 |
| *KLHL31* | ENSG00000124743.5 | 1.576542348 | 9.60E-33 | 2.11E-31 |
| *ERP27* | ENSG00000139055.5 | 1.578023336 | 1.78E-21 | 1.24E-20 |
| *ALB* | ENSG00000163631.15 | 1.579580653 | 0.02437772 | 0.030256466 |
| *TUBA4B* | ENSG00000243910.6 | 1.579614443 | 2.42E-14 | 8.34E-14 |
| *EDAR* | ENSG00000135960.8 | 1.580387347 | 6.62E-45 | 3.71E-43 |
| *HAMP* | ENSG00000105697.6 | 1.583066051 | 1.72E-09 | 3.87E-09 |
| *SLCO4A1* | ENSG00000101187.14 | 1.583688462 | 2.55E-58 | 2.68E-56 |
| *GRIN3B* | ENSG00000116032.5 | 1.587032945 | 9.03E-18 | 4.24E-17 |
| *ELFN2* | ENSG00000166897.13 | 1.59033373 | 8.06E-11 | 2.04E-10 |
| *APOF* | ENSG00000175336.9 | 1.591313343 | 0.003735863 | 0.004994714 |
| *CALML6* | ENSG00000169885.8 | 1.592019803 | 5.36E-16 | 2.16E-15 |
| *CKMT2* | ENSG00000131730.14 | 1.59204013 | 1.15E-28 | 1.71E-27 |
| *PANX2* | ENSG00000073150.12 | 1.592217048 | 1.61E-17 | 7.40E-17 |
| *MSH5* | ENSG00000204410.13 | 1.594602007 | 4.31E-34 | 1.07E-32 |
| *COL26A1* | ENSG00000160963.12 | 1.595137367 | 2.05E-08 | 4.21E-08 |
| *DAPL1* | ENSG00000163331.9 | 1.595620368 | 1.42E-13 | 4.56E-13 |
| *HIST1H3B* | ENSG00000274267.1 | 1.598880298 | 1.71E-09 | 3.85E-09 |
| *DNAH14* | ENSG00000185842.13 | 1.600409479 | 3.80E-54 | 3.34E-52 |
| *MYEOV* | ENSG00000172927.6 | 1.600824561 | 1.44E-31 | 2.84E-30 |
| *AUNIP* | ENSG00000127423.9 | 1.604264058 | 2.00E-52 | 1.65E-50 |
| *ULBP3* | ENSG00000131019.9 | 1.605180182 | 1.69E-41 | 7.53E-40 |
| *FAM169B* | ENSG00000185087.10 | 1.60638257 | 1.03E-06 | 1.83E-06 |
| *TIGD4* | ENSG00000169989.2 | 1.606662214 | 8.80E-50 | 6.42E-48 |
| *C11orf87* | ENSG00000185742.6 | 1.60741359 | 6.70E-10 | 1.56E-09 |
| *CELSR1* | ENSG00000075275.15 | 1.607650548 | 3.38E-32 | 7.13E-31 |
| *BFSP1* | ENSG00000125864.10 | 1.60865697 | 1.72E-57 | 1.76E-55 |
| *REG3A* | ENSG00000172016.14 | 1.609465885 | 1.66E-09 | 3.75E-09 |
| *C3orf67* | ENSG00000163689.17 | 1.609488168 | 2.55E-30 | 4.43E-29 |
| *SLN* | ENSG00000170290.3 | 1.609511476 | 1.25E-10 | 3.10E-10 |
| *FANCD2OS* | ENSG00000163705.11 | 1.610265503 | 1.32E-11 | 3.57E-11 |
| *FBXW12* | ENSG00000164049.13 | 1.614183266 | 5.17E-15 | 1.89E-14 |
| *TREML4* | ENSG00000188056.10 | 1.615888153 | 7.80E-07 | 1.40E-06 |
| *CXCL11* | ENSG00000169248.11 | 1.616238151 | 4.43E-14 | 1.49E-13 |
| *BMP7* | ENSG00000101144.11 | 1.61664983 | 3.58E-56 | 3.44E-54 |
| *SMC1B* | ENSG00000077935.15 | 1.617479287 | 2.73E-08 | 5.55E-08 |
| *PALM2* | ENSG00000243444.6 | 1.618615334 | 1.25E-24 | 1.21E-23 |
| *SFTPB* | ENSG00000168878.15 | 1.619468904 | 0.000211099 | 0.000311473 |
| *GRM2* | ENSG00000164082.13 | 1.624837164 | 2.51E-19 | 1.37E-18 |
| *KCTD19* | ENSG00000168676.9 | 1.629724792 | 1.12E-15 | 4.34E-15 |
| *FAM83A* | ENSG00000147689.15 | 1.634036344 | 4.79E-13 | 1.47E-12 |
| *WIF1* | ENSG00000156076.8 | 1.634102693 | 2.40E-10 | 5.82E-10 |
| *CPNE9* | ENSG00000144550.11 | 1.642595226 | 1.78E-24 | 1.69E-23 |
| *SLC22A2* | ENSG00000112499.11 | 1.643994125 | 2.83E-09 | 6.25E-09 |
| *FANCB* | ENSG00000181544.12 | 1.646541354 | 6.48E-48 | 4.45E-46 |
| *MOGAT1* | ENSG00000124003.12 | 1.647351678 | 1.04E-09 | 2.38E-09 |
| *IGFN1* | ENSG00000163395.15 | 1.648763752 | 2.40E-09 | 5.34E-09 |
| *CDSN* | ENSG00000204539.3 | 1.649582108 | 5.66E-14 | 1.88E-13 |
| *HOMER1* | ENSG00000152413.13 | 1.651502605 | 6.32E-30 | 1.06E-28 |
| *RASSF10* | ENSG00000189431.7 | 1.653056041 | 2.86E-19 | 1.55E-18 |
| *PRL* | ENSG00000172179.10 | 1.65665736 | 7.92E-07 | 1.42E-06 |
| *AKAP14* | ENSG00000186471.11 | 1.657209916 | 1.71E-11 | 4.60E-11 |
| *CLCN1* | ENSG00000188037.9 | 1.66092664 | 4.69E-09 | 1.01E-08 |
| *GAS2* | ENSG00000148935.9 | 1.661443872 | 7.81E-50 | 5.72E-48 |
| *CFAP74* | ENSG00000142609.16 | 1.661514205 | 2.19E-12 | 6.35E-12 |
| *TSACC* | ENSG00000163467.10 | 1.6632022 | 4.20E-42 | 1.97E-40 |
| *ANXA8* | ENSG00000265190.5 | 1.663337355 | 0.010662097 | 0.013673085 |
| *GDPD5* | ENSG00000158555.13 | 1.667227951 | 1.56E-76 | 3.02E-74 |
| *MYBPH* | ENSG00000133055.7 | 1.672754078 | 1.92E-09 | 4.31E-09 |
| *SLCO5A1* | ENSG00000137571.9 | 1.6728812 | 2.16E-56 | 2.13E-54 |
| *CXCL6* | ENSG00000124875.8 | 1.673452059 | 5.88E-20 | 3.44E-19 |
| *MEGF11* | ENSG00000157890.16 | 1.678535024 | 3.00E-11 | 7.89E-11 |
| *FGF17* | ENSG00000158815.9 | 1.679360299 | 6.60E-17 | 2.87E-16 |
| *OPRD1* | ENSG00000116329.9 | 1.679856248 | 2.09E-09 | 4.68E-09 |
| *SLC24A2* | ENSG00000155886.10 | 1.680060989 | 7.48E-10 | 1.74E-09 |
| *KREMEN2* | ENSG00000131650.12 | 1.68023362 | 4.04E-46 | 2.52E-44 |
| *KIAA1549* | ENSG00000122778.8 | 1.680562563 | 2.46E-65 | 3.28E-63 |
| *KCP* | ENSG00000135253.12 | 1.684096723 | 2.52E-20 | 1.53E-19 |
| *IRX2* | ENSG00000170561.11 | 1.685580332 | 2.55E-18 | 1.26E-17 |
| *KLK12* | ENSG00000186474.14 | 1.687071 | 4.95E-21 | 3.27E-20 |
| *MMP1* | ENSG00000196611.4 | 1.687367132 | 1.91E-30 | 3.37E-29 |
| *TRIM54* | ENSG00000138100.12 | 1.690659928 | 1.71E-24 | 1.63E-23 |
| *HIST1H4D* | ENSG00000277157.1 | 1.691664479 | 2.02E-11 | 5.39E-11 |
| *SLC28A3* | ENSG00000197506.7 | 1.693084601 | 1.30E-40 | 5.42E-39 |
| *GRP* | ENSG00000134443.8 | 1.702646562 | 1.61E-15 | 6.16E-15 |
| *CHRNG* | ENSG00000196811.10 | 1.705086697 | 1.22E-11 | 3.31E-11 |
| *TRIB3* | ENSG00000101255.9 | 1.708675151 | 7.98E-64 | 1.02E-61 |
| *SLC13A5* | ENSG00000141485.14 | 1.709801798 | 5.25E-05 | 8.13E-05 |
| *KCNJ14* | ENSG00000182324.6 | 1.711528421 | 1.43E-54 | 1.27E-52 |
| *CFAP46* | ENSG00000171811.11 | 1.713125376 | 6.52E-08 | 1.28E-07 |
| *ZNRF3* | ENSG00000183579.14 | 1.713440055 | 6.70E-41 | 2.87E-39 |
| *XKRX* | ENSG00000182489.8 | 1.716411501 | 2.71E-76 | 5.11E-74 |
| *MLXIPL* | ENSG00000009950.14 | 1.719875698 | 3.20E-27 | 4.12E-26 |
| *RNFT2* | ENSG00000135119.13 | 1.720213042 | 7.24E-69 | 1.13E-66 |
| *CYP2W1* | ENSG00000073067.12 | 1.720392706 | 3.10E-23 | 2.59E-22 |
| *FOSL1* | ENSG00000175592.7 | 1.723304357 | 2.17E-18 | 1.08E-17 |
| *LMTK3* | ENSG00000142235.7 | 1.730797166 | 5.41E-29 | 8.31E-28 |
| *CSMD2* | ENSG00000121904.16 | 1.731448682 | 1.44E-20 | 8.97E-20 |
| *HTRA4* | ENSG00000169495.4 | 1.732369769 | 9.24E-15 | 3.30E-14 |
| *COL22A1* | ENSG00000169436.15 | 1.732648333 | 4.46E-18 | 2.16E-17 |
| *TMEM52B* | ENSG00000165685.7 | 1.733698667 | 3.90E-25 | 3.99E-24 |
| *SERPINB5* | ENSG00000206075.12 | 1.738679688 | 5.13E-28 | 7.18E-27 |
| *CTHRC1* | ENSG00000164932.11 | 1.739424107 | 1.75E-76 | 3.34E-74 |
| *TDGF1* | ENSG00000241186.6 | 1.742512111 | 1.55E-57 | 1.61E-55 |
| *RASL10B* | ENSG00000270885.1 | 1.743044642 | 1.07E-23 | 9.36E-23 |
| *NXF3* | ENSG00000147206.15 | 1.745166605 | 5.40E-11 | 1.39E-10 |
| *PABPC1L* | ENSG00000101104.11 | 1.755762641 | 1.27E-50 | 9.63E-49 |
| *KLK11* | ENSG00000167757.12 | 1.76181979 | 3.16E-33 | 7.26E-32 |
| *CCDC33* | ENSG00000140481.12 | 1.76602719 | 1.66E-12 | 4.87E-12 |
| *ZNF670* | ENSG00000135747.10 | 1.766540864 | 1.12E-47 | 7.56E-46 |
| *SLC6A14* | ENSG00000268104.2 | 1.766867075 | 4.67E-14 | 1.57E-13 |
| *DNAAF3* | ENSG00000167646.12 | 1.778405572 | 5.78E-37 | 1.82E-35 |
| *SPACA3* | ENSG00000141316.11 | 1.780790501 | 7.49E-28 | 1.03E-26 |
| *ROBO2* | ENSG00000185008.16 | 1.783062264 | 6.70E-32 | 1.37E-30 |
| *SIGLEC15* | ENSG00000197046.10 | 1.783934227 | 1.45E-14 | 5.08E-14 |
| *PPP1R27* | ENSG00000182676.4 | 1.78421984 | 8.87E-12 | 2.45E-11 |
| *DUSP4* | ENSG00000120875.7 | 1.784757031 | 1.11E-62 | 1.36E-60 |
| *KCNV2* | ENSG00000168263.8 | 1.785104679 | 5.59E-13 | 1.70E-12 |
| *CLDN9* | ENSG00000213937.3 | 1.787064972 | 1.83E-37 | 6.00E-36 |
| *AIRE* | ENSG00000160224.15 | 1.790418295 | 7.92E-06 | 1.31E-05 |
| *GRM4* | ENSG00000124493.12 | 1.790890718 | 2.73E-06 | 4.68E-06 |
| *CAPN14* | ENSG00000214711.8 | 1.792915692 | 8.39E-14 | 2.75E-13 |
| *SLC11A1* | ENSG00000018280.15 | 1.795641417 | 1.00E-38 | 3.66E-37 |
| *IL36G* | ENSG00000136688.9 | 1.796669471 | 2.12E-10 | 5.16E-10 |
| *OLAH* | ENSG00000152463.13 | 1.800013486 | 4.58E-11 | 1.18E-10 |
| *DOK7* | ENSG00000175920.14 | 1.800936881 | 1.71E-38 | 6.14E-37 |
| *SULT2B1* | ENSG00000088002.10 | 1.801581244 | 1.12E-30 | 2.04E-29 |
| *NEK5* | ENSG00000197168.10 | 1.803002443 | 1.28E-63 | 1.61E-61 |
| *CHI3L1* | ENSG00000133048.11 | 1.805044525 | 9.54E-19 | 4.92E-18 |
| *DAW1* | ENSG00000123977.8 | 1.806669469 | 1.93E-10 | 4.72E-10 |
| *C17orf99* | ENSG00000187997.10 | 1.815755592 | 4.02E-19 | 2.15E-18 |
| *NODAL* | ENSG00000156574.8 | 1.816057471 | 9.81E-18 | 4.60E-17 |
| *SHOX2* | ENSG00000168779.18 | 1.816599144 | 1.29E-14 | 4.55E-14 |
| *AKNAD1* | ENSG00000162641.17 | 1.817553052 | 5.07E-22 | 3.74E-21 |
| *SYT8* | ENSG00000149043.15 | 1.818146945 | 4.63E-19 | 2.46E-18 |
| *HNRNPCL1* | ENSG00000179172.8 | 1.818313587 | 8.57E-11 | 2.16E-10 |
| *DUSP15* | ENSG00000149599.14 | 1.823464631 | 1.41E-41 | 6.36E-40 |
| *VNN3* | ENSG00000093134.12 | 1.826396642 | 6.32E-16 | 2.52E-15 |
| *UCN* | ENSG00000163794.6 | 1.828103192 | 7.04E-52 | 5.70E-50 |
| *KCNJ15* | ENSG00000157551.16 | 1.830372446 | 2.29E-17 | 1.04E-16 |
| *SYT12* | ENSG00000173227.12 | 1.835102923 | 1.72E-13 | 5.48E-13 |
| *PRAME* | ENSG00000185686.16 | 1.837037983 | 4.56E-21 | 3.02E-20 |
| *ATP6V1B1* | ENSG00000116039.10 | 1.837537366 | 1.27E-20 | 7.98E-20 |
| *NACA2* | ENSG00000253506.2 | 1.839077361 | 8.47E-36 | 2.43E-34 |
| *CYP2F1* | ENSG00000197446.7 | 1.842714914 | 1.47E-24 | 1.41E-23 |
| *KRT13* | ENSG00000171401.13 | 1.845813312 | 3.24E-06 | 5.53E-06 |
| *PADI1* | ENSG00000142623.9 | 1.846784107 | 4.40E-25 | 4.48E-24 |
| *DLX5* | ENSG00000105880.4 | 1.847333973 | 3.30E-13 | 1.03E-12 |
| *ASB4* | ENSG00000005981.11 | 1.84806514 | 2.11E-11 | 5.62E-11 |
| *C10orf55* | ENSG00000222047.7 | 1.85023468 | 1.74E-43 | 8.82E-42 |
| *UNC5A* | ENSG00000113763.9 | 1.852081687 | 1.13E-14 | 4.01E-14 |
| *GOLGA7B* | ENSG00000155265.9 | 1.853168722 | 2.00E-74 | 3.48E-72 |
| *ASIC4* | ENSG00000072182.11 | 1.857633089 | 4.48E-13 | 1.38E-12 |
| *GABRB1* | ENSG00000163288.12 | 1.861737186 | 6.74E-14 | 2.23E-13 |
| *SLC7A11* | ENSG00000151012.12 | 1.865822192 | 1.86E-52 | 1.55E-50 |
| *KCNH4* | ENSG00000089558.7 | 1.866106811 | 2.18E-30 | 3.81E-29 |
| *CDKN2A* | ENSG00000147889.15 | 1.867483622 | 2.34E-51 | 1.83E-49 |
| *TTLL9* | ENSG00000131044.15 | 1.868268342 | 1.45E-16 | 6.12E-16 |
| *CCNO* | ENSG00000152669.8 | 1.869554053 | 3.42E-43 | 1.70E-41 |
| *SLC6A20* | ENSG00000163817.14 | 1.871807086 | 6.83E-16 | 2.71E-15 |
| *BEAN1* | ENSG00000166546.12 | 1.875604558 | 7.86E-21 | 5.06E-20 |
| *IL23A* | ENSG00000110944.7 | 1.876782141 | 5.38E-76 | 9.79E-74 |
| *SLX1A* | ENSG00000132207.16 | 1.877937319 | 4.35E-09 | 9.43E-09 |
| *KCNT1* | ENSG00000107147.10 | 1.879683893 | 4.16E-12 | 1.18E-11 |
| *ERVMER34-1* | ENSG00000226887.6 | 1.884213347 | 4.23E-36 | 1.26E-34 |
| *NPW* | ENSG00000183971.5 | 1.885760745 | 4.24E-24 | 3.89E-23 |
| *ASGR1* | ENSG00000141505.10 | 1.893832746 | 1.61E-55 | 1.52E-53 |
| *RGS20* | ENSG00000147509.12 | 1.896104025 | 1.95E-14 | 6.77E-14 |
| *TP73* | ENSG00000078900.13 | 1.902290547 | 5.04E-45 | 2.87E-43 |
| *PTRH1* | ENSG00000187024.12 | 1.903209328 | 6.47E-28 | 8.99E-27 |
| *CCDC150* | ENSG00000144395.16 | 1.903803808 | 7.08E-62 | 8.36E-60 |
| *GABRP* | ENSG00000094755.15 | 1.905391699 | 8.79E-24 | 7.76E-23 |
| *GCKR* | ENSG00000084734.7 | 1.909098095 | 6.77E-21 | 4.38E-20 |
| *ITGBL1* | ENSG00000198542.12 | 1.9110613 | 2.86E-29 | 4.53E-28 |
| *MMP11* | ENSG00000099953.8 | 1.915028359 | 6.70E-126 | 6.91E-123 |
| *KCTD16* | ENSG00000183775.9 | 1.916003358 | 6.12E-11 | 1.56E-10 |
| *LRRC43* | ENSG00000158113.11 | 1.916571414 | 2.53E-41 | 1.11E-39 |
| *TMPRSS3* | ENSG00000160183.12 | 1.919298174 | 5.51E-67 | 7.89E-65 |
| *DNAH12* | ENSG00000174844.13 | 1.920148611 | 7.66E-27 | 9.46E-26 |
| *CNDP1* | ENSG00000150656.13 | 1.922890612 | 1.35E-07 | 2.58E-07 |
| *RFPL2* | ENSG00000128253.12 | 1.923095402 | 4.13E-05 | 6.45E-05 |
| *LHB* | ENSG00000104826.10 | 1.925570932 | 1.54E-17 | 7.10E-17 |
| *HYDIN* | ENSG00000157423.16 | 1.927209116 | 6.35E-11 | 1.62E-10 |
| *RSPO4* | ENSG00000101282.7 | 1.929266295 | 2.34E-08 | 4.78E-08 |
| *RHPN1* | ENSG00000158106.11 | 1.93236195 | 1.68E-75 | 2.99E-73 |
| *FBN3* | ENSG00000142449.11 | 1.933862187 | 0.000290666 | 0.000424739 |
| *PLK5* | ENSG00000185988.10 | 1.943223285 | 1.19E-11 | 3.24E-11 |
| *RHCG* | ENSG00000140519.11 | 1.943261212 | 1.42E-34 | 3.68E-33 |
| *ADAMTS6* | ENSG00000049192.13 | 1.944221086 | 9.55E-36 | 2.73E-34 |
| *UBE2NL* | ENSG00000276380.1 | 1.948134597 | 3.92E-12 | 1.11E-11 |
| *CARD14* | ENSG00000141527.15 | 1.949824344 | 8.79E-37 | 2.75E-35 |
| *DCDC1* | ENSG00000170959.13 | 1.953427381 | 3.94E-08 | 7.89E-08 |
| *PRSS50* | ENSG00000206549.11 | 1.958469236 | 2.75E-09 | 6.08E-09 |
| *BIRC7* | ENSG00000101197.11 | 1.960539901 | 1.64E-26 | 1.97E-25 |
| *TESC* | ENSG00000088992.16 | 1.961383272 | 5.64E-113 | 3.63E-110 |
| *CCR8* | ENSG00000179934.6 | 1.965398376 | 3.02E-30 | 5.21E-29 |
| *MEX3A* | ENSG00000254726.2 | 1.972136078 | 4.69E-76 | 8.74E-74 |
| *PALM3* | ENSG00000187867.7 | 1.972247973 | 7.63E-46 | 4.57E-44 |
| *TMEM74B* | ENSG00000125895.5 | 1.983888684 | 8.20E-35 | 2.16E-33 |
| *ABCC2* | ENSG00000023839.9 | 1.986590584 | 3.99E-10 | 9.50E-10 |
| *HGFAC* | ENSG00000109758.7 | 1.990009568 | 1.24E-07 | 2.38E-07 |
| *NPBWR1* | ENSG00000183729.3 | 1.994412322 | 2.63E-05 | 4.17E-05 |
| *STRIP2* | ENSG00000128578.8 | 2.008537745 | 3.98E-34 | 9.91E-33 |
| *XIRP1* | ENSG00000168334.8 | 2.013003128 | 1.91E-07 | 3.61E-07 |
| *GRHL1* | ENSG00000134317.16 | 2.013370874 | 1.83E-66 | 2.53E-64 |
| *IL17F* | ENSG00000112116.9 | 2.014312075 | 2.56E-11 | 6.77E-11 |
| *SPOCD1* | ENSG00000134668.11 | 2.015279558 | 5.98E-46 | 3.63E-44 |
| *AJUBA* | ENSG00000129474.14 | 2.017891914 | 2.17E-80 | 4.73E-78 |
| *SERPINA10* | ENSG00000140093.8 | 2.01883513 | 1.83E-21 | 1.27E-20 |
| *SULT1C2* | ENSG00000198203.8 | 2.020190448 | 8.39E-32 | 1.70E-30 |
| *COL7A1* | ENSG00000114270.14 | 2.020910557 | 4.58E-73 | 7.70E-71 |
| *TNFSF9* | ENSG00000125657.4 | 2.02180779 | 9.91E-78 | 2.02E-75 |
| *CFAP45* | ENSG00000213085.8 | 2.022024553 | 3.29E-66 | 4.50E-64 |
| *RNF151* | ENSG00000179580.8 | 2.023797767 | 1.79E-14 | 6.24E-14 |
| *MATN3* | ENSG00000132031.11 | 2.024165863 | 2.63E-44 | 1.41E-42 |
| *CELSR3* | ENSG00000008300.13 | 2.031150549 | 2.03E-90 | 6.83E-88 |
| *WFDC10B* | ENSG00000182931.8 | 2.032471096 | 2.57E-30 | 4.46E-29 |
| *KIAA2012* | ENSG00000182329.9 | 2.034703115 | 9.97E-28 | 1.35E-26 |
| *MPP4* | ENSG00000082126.16 | 2.044323126 | 4.28E-18 | 2.07E-17 |
| *HIST1H1E* | ENSG00000168298.5 | 2.045194373 | 9.64E-16 | 3.77E-15 |
| *C9orf50* | ENSG00000179058.6 | 2.047844305 | 7.57E-39 | 2.79E-37 |
| *SPTBN2* | ENSG00000173898.10 | 2.050394804 | 5.37E-78 | 1.12E-75 |
| *CLDN6* | ENSG00000184697.6 | 2.052688637 | 6.62E-08 | 1.30E-07 |
| *SEMG1* | ENSG00000124233.11 | 2.056728819 | 2.33E-13 | 7.35E-13 |
| *C20orf144* | ENSG00000149609.5 | 2.056989801 | 7.52E-44 | 3.89E-42 |
| *KCNH8* | ENSG00000183960.7 | 2.057391606 | 1.21E-66 | 1.70E-64 |
| *ANKUB1* | ENSG00000206199.8 | 2.057922661 | 2.77E-11 | 7.30E-11 |
| *AQP6* | ENSG00000086159.11 | 2.06936772 | 5.18E-21 | 3.41E-20 |
| *TREM1* | ENSG00000124731.11 | 2.07214751 | 6.80E-30 | 1.13E-28 |
| *CST6* | ENSG00000175315.2 | 2.072348949 | 2.14E-28 | 3.11E-27 |
| *HCN1* | ENSG00000164588.4 | 2.078073062 | 3.06E-18 | 1.50E-17 |
| *WDR72* | ENSG00000166415.13 | 2.091594145 | 3.53E-43 | 1.74E-41 |
| *SRD5A2* | ENSG00000277893.1 | 2.092008818 | 6.83E-07 | 1.23E-06 |
| *CYSRT1* | ENSG00000197191.4 | 2.093404285 | 4.37E-75 | 7.68E-73 |
| *NKX6-3* | ENSG00000165066.12 | 2.097511203 | 8.72E-07 | 1.56E-06 |
| *HIST1H4C* | ENSG00000197061.4 | 2.098995502 | 3.17E-10 | 7.62E-10 |
| *PRR7* | ENSG00000131188.10 | 2.099143544 | 1.62E-116 | 1.32E-113 |
| *DRD2* | ENSG00000149295.12 | 2.099215762 | 2.00E-27 | 2.64E-26 |
| *WNT3* | ENSG00000108379.8 | 2.104074917 | 3.28E-44 | 1.74E-42 |
| *SPATA21* | ENSG00000187144.10 | 2.112485711 | 2.32E-05 | 3.70E-05 |
| *VENTX* | ENSG00000151650.7 | 2.11257965 | 4.03E-27 | 5.14E-26 |
| *RDH16* | ENSG00000139547.7 | 2.115445931 | 1.49E-37 | 4.89E-36 |
| *BRSK2* | ENSG00000174672.14 | 2.11918996 | 1.90E-38 | 6.76E-37 |
| *SP5* | ENSG00000204335.3 | 2.129114042 | 5.90E-59 | 6.38E-57 |
| *TTC16* | ENSG00000167094.14 | 2.12966175 | 4.97E-23 | 4.07E-22 |
| *CALML3* | ENSG00000178363.4 | 2.130306774 | 1.44E-18 | 7.29E-18 |
| *KRT7* | ENSG00000135480.13 | 2.130957149 | 8.11E-39 | 2.99E-37 |
| *CYP2D7* | ENSG00000205702.9 | 2.137386669 | 7.35E-41 | 3.13E-39 |
| *EYA1* | ENSG00000104313.16 | 2.14885992 | 6.17E-16 | 2.46E-15 |
| *FUT1* | ENSG00000174951.9 | 2.150674082 | 2.11E-108 | 1.17E-105 |
| *HIST1H4A* | ENSG00000278637.1 | 2.151471154 | 1.34E-09 | 3.05E-09 |
| *CA5A* | ENSG00000174990.4 | 2.153300583 | 3.15E-11 | 8.27E-11 |
| *NT5DC4* | ENSG00000144130.10 | 2.158845392 | 1.33E-29 | 2.15E-28 |
| *LAIR2* | ENSG00000167618.8 | 2.160357286 | 5.61E-36 | 1.65E-34 |
| *KIAA1549L* | ENSG00000110427.13 | 2.173402563 | 1.14E-21 | 8.11E-21 |
| *MMP10* | ENSG00000166670.8 | 2.175098131 | 1.09E-15 | 4.23E-15 |
| *TACSTD2* | ENSG00000184292.6 | 2.175235639 | 7.29E-82 | 1.71E-79 |
| *WNT8B* | ENSG00000075290.7 | 2.178811873 | 1.79E-31 | 3.50E-30 |
| *PMCH* | ENSG00000183395.4 | 2.182419001 | 1.74E-21 | 1.21E-20 |
| *DNASE1L2* | ENSG00000167968.11 | 2.185145596 | 7.53E-68 | 1.11E-65 |
| *GSC* | ENSG00000133937.4 | 2.187985093 | 1.61E-22 | 1.25E-21 |
| *TMEM213* | ENSG00000214128.9 | 2.18858208 | 0.003110776 | 0.004187593 |
| *CGREF1* | ENSG00000138028.13 | 2.190003531 | 6.06E-48 | 4.18E-46 |
| *CITED1* | ENSG00000125931.9 | 2.190579669 | 9.06E-42 | 4.11E-40 |
| *GJB6* | ENSG00000121742.14 | 2.195763458 | 2.71E-11 | 7.15E-11 |
| *GRPR* | ENSG00000126010.5 | 2.200032748 | 2.82E-19 | 1.53E-18 |
| *FJX1* | ENSG00000179431.6 | 2.204271188 | 2.39E-81 | 5.52E-79 |
| *DPEP1* | ENSG00000015413.8 | 2.206151282 | 1.64E-24 | 1.56E-23 |
| *DKK1* | ENSG00000107984.8 | 2.208480667 | 1.55E-21 | 1.08E-20 |
| *DLX3* | ENSG00000064195.7 | 2.210110555 | 3.61E-22 | 2.71E-21 |
| *SLC38A3* | ENSG00000188338.13 | 2.211093079 | 3.87E-20 | 2.30E-19 |
| *PLA2G2F* | ENSG00000158786.4 | 2.211878085 | 7.44E-11 | 1.88E-10 |
| *ZNF750* | ENSG00000141579.6 | 2.215501474 | 3.64E-14 | 1.24E-13 |
| *NKD1* | ENSG00000140807.5 | 2.219775558 | 1.06E-51 | 8.54E-50 |
| *IL1A* | ENSG00000115008.5 | 2.22006575 | 7.79E-34 | 1.89E-32 |
| *FXYD4* | ENSG00000150201.13 | 2.220389758 | 1.04E-29 | 1.70E-28 |
| *TNNT2* | ENSG00000118194.17 | 2.220882123 | 7.52E-23 | 6.04E-22 |
| *ANKRD1* | ENSG00000148677.6 | 2.22231566 | 5.31E-10 | 1.25E-09 |
| *GPR3* | ENSG00000181773.6 | 2.237460968 | 4.27E-32 | 8.92E-31 |
| *MCEMP1* | ENSG00000183019.6 | 2.24036221 | 1.03E-23 | 9.03E-23 |
| *CYP26A1* | ENSG00000095596.10 | 2.243196618 | 1.85E-19 | 1.03E-18 |
| *CCDC148* | ENSG00000153237.16 | 2.245979134 | 1.79E-46 | 1.13E-44 |
| *CYP4Z1* | ENSG00000186160.4 | 2.246838094 | 8.48E-15 | 3.04E-14 |
| *FRMPD2* | ENSG00000170324.18 | 2.247913806 | 2.01E-11 | 5.37E-11 |
| *IL17A* | ENSG00000112115.5 | 2.24949347 | 6.74E-15 | 2.44E-14 |
| *EGR4* | ENSG00000135625.7 | 2.251458982 | 2.86E-21 | 1.95E-20 |
| *APLN* | ENSG00000171388.11 | 2.252647399 | 5.84E-59 | 6.36E-57 |
| *CALHM3* | ENSG00000183128.7 | 2.253311344 | 9.31E-17 | 4.00E-16 |
| *MSX1* | ENSG00000163132.6 | 2.255346317 | 1.04E-85 | 2.73E-83 |
| *LRP8* | ENSG00000157193.13 | 2.263748924 | 1.08E-55 | 1.02E-53 |
| *LY6G6C* | ENSG00000204421.2 | 2.263834066 | 6.32E-43 | 3.08E-41 |
| *DLL3* | ENSG00000090932.9 | 2.274658521 | 9.01E-25 | 8.86E-24 |
| *PLAC1* | ENSG00000170965.8 | 2.284511023 | 1.53E-27 | 2.04E-26 |
| *POU5F2* | ENSG00000248483.5 | 2.28533095 | 0.000195509 | 0.000289298 |
| *DSG1* | ENSG00000134760.5 | 2.285743628 | 5.08E-24 | 4.62E-23 |
| *HS6ST2* | ENSG00000171004.16 | 2.285947973 | 2.33E-71 | 3.79E-69 |
| *TFR2* | ENSG00000106327.11 | 2.294060203 | 6.55E-86 | 1.75E-83 |
| *CILP2* | ENSG00000160161.8 | 2.297865857 | 4.87E-32 | 1.01E-30 |
| *CHRNA6* | ENSG00000147434.7 | 2.310598159 | 2.26E-08 | 4.62E-08 |
| *APOA2* | ENSG00000158874.10 | 2.312418345 | 0.000129791 | 0.000195115 |
| *INHA* | ENSG00000123999.4 | 2.313836618 | 2.49E-11 | 6.60E-11 |
| *IZUMO1* | ENSG00000182264.7 | 2.314834484 | 1.45E-22 | 1.13E-21 |
| *HIST1H1B* | ENSG00000184357.4 | 2.316309194 | 9.93E-14 | 3.23E-13 |
| *DLX4* | ENSG00000108813.10 | 2.321474484 | 2.23E-57 | 2.27E-55 |
| *CACNG4* | ENSG00000075461.5 | 2.323637822 | 5.24E-30 | 8.88E-29 |
| *CLDN1* | ENSG00000163347.5 | 2.326073366 | 3.44E-80 | 7.39E-78 |
| *HIST1H2AL* | ENSG00000276903.1 | 2.328565572 | 6.06E-12 | 1.69E-11 |
| *MUCL1* | ENSG00000172551.9 | 2.329222284 | 8.37E-08 | 1.63E-07 |
| *PGLYRP4* | ENSG00000163218.13 | 2.331037605 | 8.88E-18 | 4.18E-17 |
| *MDFI* | ENSG00000112559.12 | 2.335718869 | 1.19E-59 | 1.32E-57 |
| *CREG2* | ENSG00000175874.8 | 2.338960085 | 1.29E-24 | 1.24E-23 |
| *ONECUT2* | ENSG00000119547.5 | 2.339190912 | 1.06E-35 | 3.01E-34 |
| *MS4A15* | ENSG00000166961.13 | 2.341523203 | 3.13E-16 | 1.28E-15 |
| *MAT1A* | ENSG00000151224.11 | 2.341937152 | 3.01E-44 | 1.61E-42 |
| *SIX2* | ENSG00000170577.7 | 2.344557874 | 2.98E-20 | 1.79E-19 |
| *DSG4* | ENSG00000175065.10 | 2.349384736 | 1.51E-68 | 2.27E-66 |
| *EVX1* | ENSG00000106038.11 | 2.358571032 | 1.67E-42 | 8.00E-41 |
| *RP1L1* | ENSG00000183638.5 | 2.362524788 | 4.89E-24 | 4.46E-23 |
| *TMPRSS13* | ENSG00000137747.13 | 2.363653579 | 8.22E-64 | 1.04E-61 |
| *ANXA10* | ENSG00000109511.9 | 2.367245747 | 1.11E-10 | 2.77E-10 |
| *PSORS1C2* | ENSG00000204538.3 | 2.385778034 | 5.98E-26 | 6.69E-25 |
| *ARMC3* | ENSG00000165309.12 | 2.3916931 | 1.13E-19 | 6.42E-19 |
| *KISS1* | ENSG00000170498.8 | 2.395805015 | 1.41E-22 | 1.10E-21 |
| *RIPPLY1* | ENSG00000147223.5 | 2.398787514 | 3.83E-27 | 4.90E-26 |
| *SLC6A3* | ENSG00000142319.17 | 2.40052361 | 1.14E-06 | 2.02E-06 |
| *DNAH5* | ENSG00000039139.9 | 2.405743775 | 1.00E-44 | 5.50E-43 |
| *MUC5AC* | ENSG00000215182.8 | 2.411454901 | 6.60E-13 | 2.01E-12 |
| *FAM131C* | ENSG00000185519.8 | 2.415691147 | 9.04E-44 | 4.64E-42 |
| *LRRC8E* | ENSG00000171017.9 | 2.42303355 | 5.47E-74 | 9.40E-72 |
| *ITIH6* | ENSG00000102313.8 | 2.435918524 | 1.11E-17 | 5.18E-17 |
| *SHISA2* | ENSG00000180730.4 | 2.438324846 | 2.82E-93 | 9.69E-91 |
| *FAP* | ENSG00000078098.12 | 2.443191798 | 8.79E-42 | 4.01E-40 |
| *FGA* | ENSG00000171560.13 | 2.450813077 | 1.08E-06 | 1.92E-06 |
| *MYADML2* | ENSG00000185105.4 | 2.452746802 | 1.34E-40 | 5.57E-39 |
| *STC2* | ENSG00000113739.9 | 2.454650231 | 2.44E-50 | 1.82E-48 |
| *FABP6* | ENSG00000170231.14 | 2.456134134 | 9.99E-12 | 2.74E-11 |
| *NTF4* | ENSG00000225950.6 | 2.459193232 | 8.66E-07 | 1.55E-06 |
| *NKD2* | ENSG00000145506.12 | 2.464543949 | 1.57E-113 | 1.06E-110 |
| *OXTR* | ENSG00000180914.9 | 2.467320413 | 3.67E-77 | 7.28E-75 |
| *NEB* | ENSG00000183091.18 | 2.468351918 | 2.98E-23 | 2.49E-22 |
| *GABRD* | ENSG00000187730.7 | 2.469297422 | 2.98E-99 | 1.18E-96 |
| *PRSS22* | ENSG00000005001.8 | 2.474754369 | 5.32E-40 | 2.14E-38 |
| *SIX4* | ENSG00000100625.8 | 2.479189345 | 4.54E-35 | 1.22E-33 |
| *TMPRSS5* | ENSG00000166682.9 | 2.479759368 | 4.21E-36 | 1.26E-34 |
| *GCM1* | ENSG00000137270.10 | 2.480984305 | 9.47E-13 | 2.84E-12 |
| *S100A2* | ENSG00000196754.9 | 2.48804321 | 1.40E-101 | 6.37E-99 |
| *CDH7* | ENSG00000081138.12 | 2.500573859 | 2.77E-10 | 6.68E-10 |
| *MMP3* | ENSG00000149968.10 | 2.502598037 | 4.61E-36 | 1.36E-34 |
| *ORM2* | ENSG00000228278.3 | 2.505721438 | 3.04E-26 | 3.50E-25 |
| *IGFL2* | ENSG00000204866.7 | 2.507645731 | 1.05E-37 | 3.49E-36 |
| *FOLR1* | ENSG00000110195.10 | 2.514002458 | 7.06E-45 | 3.94E-43 |
| *CA9* | ENSG00000107159.11 | 2.5205444 | 4.74E-88 | 1.47E-85 |
| *KAAG1* | ENSG00000146049.1 | 2.520936227 | 9.74E-22 | 6.98E-21 |
| *WNT7A* | ENSG00000154764.5 | 2.522342535 | 7.41E-07 | 1.33E-06 |
| *GLS2* | ENSG00000135423.11 | 2.523252079 | 2.63E-87 | 7.82E-85 |
| *SPAG17* | ENSG00000155761.12 | 2.535396472 | 6.30E-19 | 3.31E-18 |
| *LCN1* | ENSG00000160349.8 | 2.538000947 | 1.85E-10 | 4.53E-10 |
| *FGL1* | ENSG00000104760.15 | 2.548481219 | 4.26E-06 | 7.19E-06 |
| *ADAMTS12* | ENSG00000151388.9 | 2.560649465 | 1.27E-84 | 3.22E-82 |
| *FOXD1* | ENSG00000251493.3 | 2.570328885 | 9.33E-20 | 5.34E-19 |
| *MYO18B* | ENSG00000133454.14 | 2.572728098 | 6.79E-12 | 1.89E-11 |
| *OLR1* | ENSG00000173391.7 | 2.573099783 | 1.04E-66 | 1.48E-64 |
| *POU4F1* | ENSG00000152192.7 | 2.575926299 | 5.13E-24 | 4.66E-23 |
| *WDR66* | ENSG00000158023.8 | 2.578117781 | 5.65E-74 | 9.60E-72 |
| *HAGHL* | ENSG00000103253.16 | 2.587116461 | 2.45E-110 | 1.40E-107 |
| *OBP2A* | ENSG00000122136.12 | 2.591666725 | 3.54E-08 | 7.12E-08 |
| *RNF183* | ENSG00000165188.12 | 2.59350926 | 4.51E-87 | 1.32E-84 |
| *TAS2R38* | ENSG00000257138.1 | 2.599517043 | 9.57E-41 | 4.02E-39 |
| *TRIM29* | ENSG00000137699.15 | 2.60191614 | 1.21E-87 | 3.67E-85 |
| *SPTBN5* | ENSG00000137877.9 | 2.602641785 | 1.77E-60 | 2.04E-58 |
| *CYP4F8* | ENSG00000186526.11 | 2.604250866 | 7.91E-10 | 1.83E-09 |
| *KLK10* | ENSG00000129451.10 | 2.60601905 | 6.11E-95 | 2.15E-92 |
| *HBQ1* | ENSG00000086506.2 | 2.60646483 | 6.67E-18 | 3.17E-17 |
| *CPN1* | ENSG00000120054.10 | 2.608984392 | 3.10E-21 | 2.10E-20 |
| *C2orf70* | ENSG00000173557.13 | 2.612128873 | 4.80E-81 | 1.06E-78 |
| *CBX2* | ENSG00000173894.9 | 2.620986631 | 1.34E-115 | 9.87E-113 |
| *ETV4* | ENSG00000175832.11 | 2.630309309 | 2.50E-86 | 6.90E-84 |
| *TBX15* | ENSG00000092607.12 | 2.645068102 | 1.70E-38 | 6.11E-37 |
| *CXCL5* | ENSG00000163735.6 | 2.652082008 | 2.01E-30 | 3.54E-29 |
| *CLDN14* | ENSG00000159261.9 | 2.66219793 | 3.20E-38 | 1.11E-36 |
| *PRSS33* | ENSG00000103355.11 | 2.667492115 | 1.73E-42 | 8.26E-41 |
| *C19orf81* | ENSG00000235034.5 | 2.670868743 | 2.41E-15 | 9.08E-15 |
| *C2CD4A* | ENSG00000198535.5 | 2.677866884 | 1.11E-56 | 1.11E-54 |
| *DNAH2* | ENSG00000183914.13 | 2.67870857 | 1.66E-55 | 1.56E-53 |
| *MYH4* | ENSG00000264424.1 | 2.67918274 | 4.03E-13 | 1.25E-12 |
| *HOGA1* | ENSG00000241935.7 | 2.680614296 | 2.45E-43 | 1.23E-41 |
| *EVA1A* | ENSG00000115363.12 | 2.681526508 | 2.97E-119 | 2.55E-116 |
| *CDC20B* | ENSG00000164287.11 | 2.688741715 | 6.52E-05 | 0.000100267 |
| *LRRC36* | ENSG00000159708.16 | 2.692045411 | 5.10E-76 | 9.39E-74 |
| *NR5A1* | ENSG00000136931.8 | 2.696389021 | 1.02E-05 | 1.67E-05 |
| *HIST1H3J* | ENSG00000197153.4 | 2.701933329 | 4.77E-13 | 1.46E-12 |
| *KIF26B* | ENSG00000162849.14 | 2.704265469 | 1.45E-59 | 1.60E-57 |
| *ISM2* | ENSG00000100593.16 | 2.711910901 | 2.25E-25 | 2.36E-24 |
| *SBSN* | ENSG00000189001.9 | 2.714105963 | 2.71E-08 | 5.51E-08 |
| *CSF2* | ENSG00000164400.5 | 2.722577856 | 5.94E-38 | 2.01E-36 |
| *TMPRSS11E* | ENSG00000087128.8 | 2.743681213 | 3.66E-06 | 6.21E-06 |
| *FGF8* | ENSG00000107831.11 | 2.748641772 | 4.11E-13 | 1.27E-12 |
| *NANOS3* | ENSG00000187556.7 | 2.751914233 | 1.75E-62 | 2.11E-60 |
| *NPC1L1* | ENSG00000015520.13 | 2.756035111 | 3.07E-28 | 4.37E-27 |
| *ACAN* | ENSG00000157766.14 | 2.756559516 | 4.81E-33 | 1.08E-31 |
| *PLEKHN1* | ENSG00000187583.9 | 2.772049242 | 1.23E-129 | 1.46E-126 |
| *GLYATL1* | ENSG00000166840.12 | 2.776853969 | 9.65E-56 | 9.21E-54 |
| *HIST1H3I* | ENSG00000275379.1 | 2.777900116 | 2.03E-10 | 4.95E-10 |
| *PKP1* | ENSG00000081277.10 | 2.777950119 | 8.17E-66 | 1.10E-63 |
| *KISS1R* | ENSG00000116014.8 | 2.780730904 | 1.04E-23 | 9.11E-23 |
| *F7* | ENSG00000057593.12 | 2.785064685 | 9.66E-27 | 1.18E-25 |
| *TNNT1* | ENSG00000105048.15 | 2.786196001 | 6.94E-39 | 2.58E-37 |
| *TBX4* | ENSG00000121075.8 | 2.786269892 | 4.65E-11 | 1.20E-10 |
| *SERPINA7* | ENSG00000123561.13 | 2.786474981 | 9.20E-21 | 5.87E-20 |
| *TSPEAR* | ENSG00000175894.13 | 2.790371417 | 2.24E-42 | 1.06E-40 |
| *POU6F2* | ENSG00000106536.18 | 2.792796005 | 9.42E-21 | 6.01E-20 |
| *GSDMC* | ENSG00000147697.7 | 2.796122954 | 3.06E-37 | 9.86E-36 |
| *IGFBP1* | ENSG00000146678.8 | 2.798258915 | 1.72E-20 | 1.06E-19 |
| *IL17C* | ENSG00000124391.4 | 2.811789834 | 5.42E-25 | 5.47E-24 |
| *PERM1* | ENSG00000187642.8 | 2.814070785 | 6.80E-96 | 2.50E-93 |
| *TFAP2A* | ENSG00000137203.9 | 2.816401675 | 1.17E-58 | 1.25E-56 |
| *CLDN16* | ENSG00000113946.3 | 2.819082757 | 2.20E-39 | 8.49E-38 |
| *ANKRD33* | ENSG00000167612.11 | 2.820965478 | 7.31E-13 | 2.21E-12 |
| *PAX9* | ENSG00000198807.11 | 2.822934952 | 3.42E-66 | 4.64E-64 |
| *KCNK9* | ENSG00000169427.5 | 2.82477467 | 2.91E-29 | 4.60E-28 |
| *CEMIP* | ENSG00000103888.14 | 2.826895709 | 6.31E-128 | 6.97E-125 |
| *GJB5* | ENSG00000189280.3 | 2.830925616 | 4.38E-44 | 2.30E-42 |
| *A2ML1* | ENSG00000166535.18 | 2.832056455 | 0.000201928 | 0.000298425 |
| *EGFL6* | ENSG00000198759.10 | 2.837443146 | 6.21E-87 | 1.78E-84 |
| *IRX3* | ENSG00000177508.11 | 2.838543582 | 8.51E-33 | 1.88E-31 |
| *PRKCG* | ENSG00000126583.9 | 2.845280027 | 1.89E-37 | 6.18E-36 |
| *PAX2* | ENSG00000075891.20 | 2.852371722 | 4.58E-07 | 8.40E-07 |
| *RNF182* | ENSG00000180537.11 | 2.855902438 | 4.29E-21 | 2.86E-20 |
| *NOX4* | ENSG00000086991.11 | 2.858991892 | 1.23E-49 | 8.93E-48 |
| *CLEC5A* | ENSG00000258227.5 | 2.869124163 | 2.66E-56 | 2.59E-54 |
| *DSC3* | ENSG00000134762.15 | 2.873398502 | 4.39E-33 | 9.93E-32 |
| *DCSTAMP* | ENSG00000164935.5 | 2.876271229 | 9.46E-22 | 6.80E-21 |
| *IZUMO2* | ENSG00000161652.11 | 2.897551444 | 2.03E-25 | 2.14E-24 |
| *ALDH3B2* | ENSG00000132746.13 | 2.911562996 | 1.53E-49 | 1.11E-47 |
| *KRT17* | ENSG00000128422.14 | 2.912893228 | 2.58E-49 | 1.84E-47 |
| *UNC93A* | ENSG00000112494.8 | 2.915233166 | 6.27E-41 | 2.71E-39 |
| *HCRT* | ENSG00000161610.1 | 2.916030503 | 1.37E-32 | 2.96E-31 |
| *EN1* | ENSG00000163064.6 | 2.921517969 | 3.37E-10 | 8.08E-10 |
| *ADAM12* | ENSG00000148848.13 | 2.92167623 | 1.51E-76 | 2.96E-74 |
| *CALB1* | ENSG00000104327.6 | 2.924426909 | 8.50E-12 | 2.35E-11 |
| *UPK1A* | ENSG00000105668.6 | 2.925996006 | 1.03E-14 | 3.67E-14 |
| *LRRN4* | ENSG00000125872.7 | 2.930215736 | 8.41E-34 | 2.04E-32 |
| *ATG9B* | ENSG00000181652.17 | 2.939070347 | 3.05E-95 | 1.10E-92 |
| *TEX19* | ENSG00000182459.4 | 2.943753094 | 2.56E-14 | 8.79E-14 |
| *SLC35D3* | ENSG00000182747.4 | 2.950716709 | 1.48E-63 | 1.85E-61 |
| *CLDN2* | ENSG00000165376.9 | 2.950887833 | 1.08E-47 | 7.33E-46 |
| *PPBP* | ENSG00000163736.3 | 2.953920618 | 1.70E-30 | 3.03E-29 |
| *ZNF556* | ENSG00000172000.6 | 2.956605662 | 6.92E-14 | 2.28E-13 |
| *PRDM12* | ENSG00000130711.3 | 2.963423213 | 4.61E-64 | 5.94E-62 |
| *UROC1* | ENSG00000159650.7 | 2.969341737 | 4.74E-10 | 1.12E-09 |
| *C1orf105* | ENSG00000180999.9 | 2.977178997 | 2.55E-14 | 8.76E-14 |
| *ORM1* | ENSG00000229314.5 | 2.983836697 | 2.88E-12 | 8.27E-12 |
| *HCAR1* | ENSG00000196917.5 | 2.990265213 | 8.27E-32 | 1.68E-30 |
| *PPEF1* | ENSG00000086717.17 | 2.991190637 | 3.03E-53 | 2.59E-51 |
| *SH3TC2* | ENSG00000169247.10 | 2.995220942 | 1.96E-121 | 1.78E-118 |
| *SERPIND1* | ENSG00000099937.9 | 3.001681075 | 2.82E-31 | 5.40E-30 |
| *HTR1D* | ENSG00000179546.4 | 3.005290878 | 7.18E-24 | 6.40E-23 |
| *MIOX* | ENSG00000100253.11 | 3.008981234 | 3.68E-23 | 3.05E-22 |
| *SLC4A11* | ENSG00000088836.11 | 3.011515577 | 1.06E-101 | 4.97E-99 |
| *NMUR2* | ENSG00000132911.4 | 3.026222813 | 6.22E-12 | 1.74E-11 |
| *TH* | ENSG00000180176.13 | 3.028786354 | 4.16E-35 | 1.12E-33 |
| *LY6G6D* | ENSG00000244355.6 | 3.037850649 | 8.89E-51 | 6.81E-49 |
| *GJA3* | ENSG00000121743.3 | 3.046420634 | 1.33E-19 | 7.51E-19 |
| *HIST1H2AB* | ENSG00000278463.1 | 3.04776619 | 5.78E-10 | 1.36E-09 |
| *HIST1H4B* | ENSG00000278705.1 | 3.050648568 | 1.55E-11 | 4.18E-11 |
| *SSTR5* | ENSG00000162009.8 | 3.054963247 | 4.63E-45 | 2.65E-43 |
| *TBX20* | ENSG00000164532.10 | 3.060021982 | 1.54E-41 | 6.90E-40 |
| *POU3F2* | ENSG00000184486.8 | 3.062966664 | 2.21E-08 | 4.52E-08 |
| *KLHL35* | ENSG00000149243.14 | 3.06393931 | 1.18E-89 | 3.72E-87 |
| *CCDC78* | ENSG00000162004.15 | 3.065303093 | 7.84E-84 | 1.96E-81 |
| *GPRC6A* | ENSG00000173612.8 | 3.068595569 | 1.79E-10 | 4.38E-10 |
| *HIST1H2BI* | ENSG00000278588.1 | 3.071050185 | 3.59E-08 | 7.21E-08 |
| *GRHL3* | ENSG00000158055.14 | 3.076228578 | 1.01E-99 | 4.11E-97 |
| *AMH* | ENSG00000104899.5 | 3.07670813 | 2.03E-56 | 2.01E-54 |
| *DSG3* | ENSG00000134757.4 | 3.08442649 | 7.95E-23 | 6.36E-22 |
| *HES7* | ENSG00000179111.7 | 3.090587228 | 9.58E-19 | 4.94E-18 |
| *PDX1* | ENSG00000139515.5 | 3.092409988 | 2.17E-100 | 9.32E-98 |
| *PRR36* | ENSG00000183248.10 | 3.109373986 | 1.07E-100 | 4.73E-98 |
| *SLC13A3* | ENSG00000158296.12 | 3.115373343 | 1.75E-46 | 1.11E-44 |
| *PNPLA3* | ENSG00000100344.9 | 3.115829287 | 1.65E-45 | 9.70E-44 |
| *NKPD1* | ENSG00000179846.8 | 3.122716542 | 4.31E-53 | 3.64E-51 |
| *CORIN* | ENSG00000145244.10 | 3.130764938 | 1.99E-62 | 2.39E-60 |
| *SH2D5* | ENSG00000189410.10 | 3.142382772 | 8.22E-28 | 1.13E-26 |
| *GNGT1* | ENSG00000127928.11 | 3.142728284 | 1.74E-41 | 7.73E-40 |
| *NPFFR1* | ENSG00000148734.7 | 3.143873756 | 4.35E-81 | 9.75E-79 |
| *MSX2* | ENSG00000120149.8 | 3.163802981 | 3.41E-135 | 4.79E-132 |
| *GRIN2B* | ENSG00000273079.3 | 3.167110697 | 1.82E-82 | 4.33E-80 |
| *PRSS1* | ENSG00000204983.11 | 3.169441441 | 6.54E-18 | 3.11E-17 |
| *VTCN1* | ENSG00000134258.15 | 3.171718732 | 1.04E-15 | 4.05E-15 |
| *UPK2* | ENSG00000110375.2 | 3.171821855 | 5.85E-17 | 2.56E-16 |
| *GRIN2D* | ENSG00000105464.3 | 3.172604098 | 3.12E-147 | 6.03E-144 |
| *ALOXE3* | ENSG00000179148.8 | 3.177010933 | 5.25E-32 | 1.09E-30 |
| *CSTL1* | ENSG00000125823.11 | 3.180116247 | 2.63E-30 | 4.56E-29 |
| *F2* | ENSG00000180210.13 | 3.183573117 | 3.78E-26 | 4.31E-25 |
| *KIAA1257* | ENSG00000114656.9 | 3.183573716 | 2.55E-131 | 3.29E-128 |
| *RIPPLY3* | ENSG00000183145.7 | 3.194316188 | 1.58E-57 | 1.63E-55 |
| *SPATA12* | ENSG00000186451.1 | 3.198397958 | 5.86E-116 | 4.53E-113 |
| *CEL* | ENSG00000170835.13 | 3.200202457 | 1.37E-66 | 1.91E-64 |
| *C6orf223* | ENSG00000181577.14 | 3.202127337 | 2.31E-55 | 2.15E-53 |
| *GJB4* | ENSG00000189433.5 | 3.203797578 | 5.54E-80 | 1.17E-77 |
| *KRT4* | ENSG00000170477.11 | 3.22897164 | 2.02E-10 | 4.92E-10 |
| *SLAMF9* | ENSG00000162723.8 | 3.230024274 | 2.72E-43 | 1.36E-41 |
| *AQP5* | ENSG00000161798.6 | 3.246307677 | 1.58E-17 | 7.27E-17 |
| *MAPK15* | ENSG00000181085.13 | 3.256735461 | 6.89E-100 | 2.88E-97 |
| *RHBG* | ENSG00000132677.11 | 3.259357475 | 2.31E-18 | 1.15E-17 |
| *SMKR1* | ENSG00000240204.2 | 3.263062639 | 8.65E-108 | 4.61E-105 |
| *STK31* | ENSG00000196335.11 | 3.275321696 | 1.08E-64 | 1.40E-62 |
| *ACSL6* | ENSG00000164398.11 | 3.277598136 | 1.95E-68 | 2.90E-66 |
| *BTBD16* | ENSG00000138152.8 | 3.285733069 | 4.66E-83 | 1.13E-80 |
| *HOXC11* | ENSG00000123388.4 | 3.286959707 | 1.30E-16 | 5.50E-16 |
| *ULBP2* | ENSG00000131015.4 | 3.301141498 | 2.60E-81 | 5.91E-79 |
| *SERPINB2* | ENSG00000197632.7 | 3.304786597 | 4.49E-18 | 2.17E-17 |
| *AHSG* | ENSG00000145192.11 | 3.310952828 | 3.65E-10 | 8.73E-10 |
| *APELA* | ENSG00000248329.4 | 3.31830687 | 2.48E-62 | 2.95E-60 |
| *CALCA* | ENSG00000110680.11 | 3.325022738 | 1.43E-23 | 1.23E-22 |
| *NAT16* | ENSG00000167011.7 | 3.338870249 | 2.26E-18 | 1.13E-17 |
| *SAA4* | ENSG00000148965.7 | 3.342848003 | 1.41E-20 | 8.79E-20 |
| *GRK1* | ENSG00000185974.6 | 3.350028377 | 7.23E-30 | 1.20E-28 |
| *RGR* | ENSG00000148604.12 | 3.35094425 | 8.53E-15 | 3.06E-14 |
| *PADI3* | ENSG00000142619.4 | 3.354050459 | 1.02E-20 | 6.47E-20 |
| *IGF2BP3* | ENSG00000136231.12 | 3.366848653 | 1.79E-34 | 4.59E-33 |
| *FGF19* | ENSG00000162344.3 | 3.369711617 | 1.10E-13 | 3.57E-13 |
| *FGB* | ENSG00000171564.10 | 3.372265979 | 9.02E-15 | 3.23E-14 |
| *TLX1* | ENSG00000107807.11 | 3.384572853 | 5.46E-85 | 1.41E-82 |
| *CACNG8* | ENSG00000142408.2 | 3.403871183 | 1.06E-43 | 5.43E-42 |
| *SIX1* | ENSG00000126778.8 | 3.420239071 | 1.41E-31 | 2.79E-30 |
| *HS3ST4* | ENSG00000182601.6 | 3.436561657 | 1.34E-06 | 2.36E-06 |
| *IL11* | ENSG00000095752.5 | 3.445196112 | 8.21E-69 | 1.26E-66 |
| *EPHX4* | ENSG00000172031.6 | 3.445289103 | 3.52E-156 | 9.07E-153 |
| *KRT6A* | ENSG00000205420.9 | 3.461309676 | 1.29E-21 | 9.10E-21 |
| *ASCL5* | ENSG00000232237.3 | 3.471581447 | 2.73E-72 | 4.54E-70 |
| *FOXQ1* | ENSG00000164379.5 | 3.486905944 | 7.94E-177 | 4.09E-173 |
| *PLA2G3* | ENSG00000100078.3 | 3.49645361 | 4.98E-35 | 1.33E-33 |
| *LRP2* | ENSG00000081479.11 | 3.505067355 | 8.23E-10 | 1.91E-09 |
| *C9orf57* | ENSG00000204669.8 | 3.51819305 | 6.50E-23 | 5.26E-22 |
| *DIRC1* | ENSG00000174325.4 | 3.525225728 | 2.41E-24 | 2.27E-23 |
| *CLDN18* | ENSG00000066405.11 | 3.533425957 | 2.95E-15 | 1.10E-14 |
| *IGF2BP1* | ENSG00000159217.8 | 3.535705625 | 1.02E-16 | 4.36E-16 |
| *NXPH4* | ENSG00000182379.9 | 3.54910988 | 7.29E-47 | 4.74E-45 |
| *SIX3* | ENSG00000138083.4 | 3.558285705 | 9.83E-16 | 3.84E-15 |
| *ABCA12* | ENSG00000144452.13 | 3.559766216 | 1.46E-22 | 1.14E-21 |
| *LY6D* | ENSG00000167656.4 | 3.567332505 | 9.12E-21 | 5.82E-20 |
| *TG* | ENSG00000042832.10 | 3.568730076 | 1.99E-69 | 3.14E-67 |
| *DLX6* | ENSG00000006377.10 | 3.580976382 | 2.85E-33 | 6.58E-32 |
| *CPNE7* | ENSG00000178773.13 | 3.58515314 | 1.57E-164 | 6.07E-161 |
| *KRT6B* | ENSG00000185479.5 | 3.592007277 | 1.11E-40 | 4.65E-39 |
| *DHRS2* | ENSG00000100867.13 | 3.608879067 | 5.65E-40 | 2.27E-38 |
| *SIM2* | ENSG00000159263.14 | 3.614431313 | 5.70E-156 | 1.26E-152 |
| *PGC* | ENSG00000096088.15 | 3.61968202 | 1.85E-35 | 5.12E-34 |
| *CNTD2* | ENSG00000105219.7 | 3.640832092 | 9.04E-112 | 5.38E-109 |
| *IL36RN* | ENSG00000136695.13 | 3.650351793 | 6.28E-10 | 1.47E-09 |
| *CAMKV* | ENSG00000164076.15 | 3.699276846 | 2.00E-26 | 2.37E-25 |
| *TCP11* | ENSG00000124678.16 | 3.716971806 | 3.14E-14 | 1.07E-13 |
| *INHBA* | ENSG00000122641.9 | 3.729542758 | 1.33E-144 | 2.06E-141 |
| *BAAT* | ENSG00000136881.10 | 3.736842319 | 2.66E-13 | 8.36E-13 |
| *ULBP1* | ENSG00000111981.4 | 3.745241634 | 3.37E-71 | 5.43E-69 |
| *GATA4* | ENSG00000136574.16 | 3.748830692 | 3.08E-18 | 1.51E-17 |
| *PIWIL1* | ENSG00000125207.6 | 3.754665209 | 4.27E-39 | 1.62E-37 |
| *CDH3* | ENSG00000062038.12 | 3.76051337 | 5.41E-86 | 1.47E-83 |
| *ATP6V0A4* | ENSG00000105929.14 | 3.78996501 | 1.97E-11 | 5.26E-11 |
| *SOX1* | ENSG00000182968.4 | 3.791667237 | 2.21E-31 | 4.28E-30 |
| *MMP20* | ENSG00000137674.3 | 3.807930068 | 1.01E-19 | 5.77E-19 |
| *CXCL17* | ENSG00000189377.7 | 3.813749467 | 3.46E-29 | 5.42E-28 |
| *S100A7* | ENSG00000143556.7 | 3.816756401 | 1.48E-13 | 4.74E-13 |
| *COMP* | ENSG00000105664.9 | 3.827455146 | 2.09E-77 | 4.20E-75 |
| *CST5* | ENSG00000170367.4 | 3.87565948 | 1.63E-17 | 7.49E-17 |
| *DUSP9* | ENSG00000130829.16 | 3.883769306 | 6.00E-34 | 1.47E-32 |
| *PRDM13* | ENSG00000112238.11 | 3.893604094 | 1.82E-24 | 1.73E-23 |
| *WFDC10A* | ENSG00000180305.4 | 3.902710491 | 6.71E-42 | 3.10E-40 |
| *CHST4* | ENSG00000140835.9 | 3.909405602 | 1.14E-36 | 3.51E-35 |
| *GAST* | ENSG00000184502.3 | 3.922237878 | 1.18E-22 | 9.30E-22 |
| *AQP2* | ENSG00000167580.6 | 3.966178744 | 5.01E-14 | 1.67E-13 |
| *SLC22A11* | ENSG00000168065.14 | 3.968063507 | 1.33E-50 | 1.00E-48 |
| *KRT16* | ENSG00000186832.7 | 3.980222308 | 3.44E-29 | 5.39E-28 |
| *SLCO1A2* | ENSG00000084453.15 | 3.985192911 | 1.35E-14 | 4.75E-14 |
| *MUC16* | ENSG00000181143.14 | 3.986238684 | 1.87E-07 | 3.54E-07 |
| *MUC6* | ENSG00000184956.14 | 3.997771773 | 1.40E-16 | 5.91E-16 |
| *KRT23* | ENSG00000108244.15 | 3.998635779 | 6.71E-107 | 3.46E-104 |
| *TMEM40* | ENSG00000088726.14 | 4.001699254 | 6.81E-19 | 3.56E-18 |
| *BEST3* | ENSG00000127325.17 | 4.017978418 | 1.92E-11 | 5.14E-11 |
| *SEC14L4* | ENSG00000133488.13 | 4.028539281 | 1.95E-22 | 1.50E-21 |
| *AFP* | ENSG00000081051.6 | 4.048405475 | 1.12E-12 | 3.33E-12 |
| *KRT83* | ENSG00000170523.3 | 4.057042208 | 1.88E-25 | 1.99E-24 |
| *SERPINB7* | ENSG00000166396.11 | 4.089455325 | 2.48E-14 | 8.53E-14 |
| *LHX5* | ENSG00000089116.3 | 4.125825863 | 4.08E-20 | 2.43E-19 |
| *TCN1* | ENSG00000134827.6 | 4.131084279 | 7.84E-69 | 1.21E-66 |
| *SALL4* | ENSG00000101115.11 | 4.167301905 | 1.29E-97 | 4.99E-95 |
| *TNNI3* | ENSG00000129991.11 | 4.170079723 | 7.27E-65 | 9.53E-63 |
| *DDN* | ENSG00000181418.7 | 4.179041927 | 5.68E-123 | 5.49E-120 |
| *MMP7* | ENSG00000137673.7 | 4.179435076 | 7.36E-107 | 3.67E-104 |
| *IFNE* | ENSG00000184995.7 | 4.198981467 | 7.57E-16 | 2.99E-15 |
| *OFCC1* | ENSG00000181355.19 | 4.20972652 | 1.08E-18 | 5.53E-18 |
| *DMRT3* | ENSG00000064218.4 | 4.214142993 | 1.42E-16 | 6.00E-16 |
| *SP8* | ENSG00000164651.15 | 4.21651218 | 2.22E-19 | 1.22E-18 |
| *IRX5* | ENSG00000176842.13 | 4.241428042 | 8.33E-69 | 1.26E-66 |
| *MMP8* | ENSG00000118113.10 | 4.272513595 | 5.39E-14 | 1.80E-13 |
| *WT1* | ENSG00000184937.11 | 4.339666337 | 2.76E-49 | 1.96E-47 |
| *SPRR3* | ENSG00000163209.13 | 4.371754772 | 1.77E-14 | 6.17E-14 |
| *WNT2* | ENSG00000105989.7 | 4.384739851 | 2.29E-163 | 7.08E-160 |
| *TRIM72* | ENSG00000177238.12 | 4.407126636 | 1.91E-22 | 1.47E-21 |
| *LPO* | ENSG00000167419.9 | 4.420886193 | 7.92E-24 | 7.03E-23 |
| *TCF24* | ENSG00000261787.1 | 4.421937922 | 4.54E-52 | 3.70E-50 |
| *EPHA8* | ENSG00000070886.9 | 4.427668925 | 1.59E-05 | 2.57E-05 |
| *MMP13* | ENSG00000137745.10 | 4.480168369 | 9.46E-37 | 2.95E-35 |
| *STRA6* | ENSG00000137868.17 | 4.551922904 | 9.40E-76 | 1.69E-73 |
| *ZIC2* | ENSG00000043355.9 | 4.6545964 | 2.27E-53 | 1.95E-51 |
| *DUSP27* | ENSG00000198842.8 | 4.69121774 | 2.07E-53 | 1.80E-51 |
| *COL11A1* | ENSG00000060718.17 | 4.726749684 | 3.94E-112 | 2.44E-109 |
| *ESM1* | ENSG00000164283.11 | 4.730110408 | 3.66E-192 | 2.83E-188 |
| *DMRTA2* | ENSG00000142700.10 | 4.731287943 | 6.36E-39 | 2.39E-37 |
| *AKR1C4* | ENSG00000198610.9 | 4.767155477 | 1.40E-37 | 4.62E-36 |
| *KRT80* | ENSG00000167767.12 | 4.798199235 | 1.29E-235 | 2.00E-231 |
| *PGLYRP3* | ENSG00000159527.3 | 4.815028128 | 1.82E-17 | 8.32E-17 |
| *PAEP* | ENSG00000122133.15 | 4.859404628 | 1.42E-17 | 6.57E-17 |
| *CST2* | ENSG00000170369.3 | 4.928395271 | 1.13E-89 | 3.64E-87 |
| *PAH* | ENSG00000171759.7 | 4.944758336 | 9.14E-40 | 3.62E-38 |
| *CST1* | ENSG00000170373.7 | 4.968006081 | 1.21E-144 | 2.06E-141 |
| *SLC26A9* | ENSG00000174502.17 | 4.994982213 | 8.78E-12 | 2.42E-11 |
| *LEMD1* | ENSG00000186007.8 | 5.041355252 | 3.24E-90 | 1.07E-87 |
| *AMELX* | ENSG00000125363.13 | 5.046460619 | 1.41E-50 | 1.06E-48 |
| *GAD1* | ENSG00000128683.12 | 5.087880623 | 1.55E-62 | 1.89E-60 |
| *FGF20* | ENSG00000078579.8 | 5.095592784 | 3.51E-26 | 4.02E-25 |
| *SPRR2A* | ENSG00000241794.1 | 5.220994395 | 2.36E-24 | 2.22E-23 |
| *HABP2* | ENSG00000148702.13 | 5.235183622 | 3.42E-24 | 3.16E-23 |
| *SPRR1B* | ENSG00000169469.8 | 5.247037047 | 7.15E-25 | 7.11E-24 |
| *MAGEA11* | ENSG00000185247.13 | 5.28094242 | 1.13E-17 | 5.27E-17 |
| *AKAP4* | ENSG00000147081.13 | 5.308166042 | 4.09E-10 | 9.73E-10 |
| *OTX1* | ENSG00000115507.8 | 5.359034227 | 1.57E-113 | 1.06E-110 |
| *SLCO1B3* | ENSG00000111700.11 | 5.360534407 | 2.20E-53 | 1.90E-51 |
| *TRIM71* | ENSG00000206557.5 | 5.361432219 | 3.97E-13 | 1.23E-12 |
| *WNT7B* | ENSG00000188064.8 | 5.364104568 | 4.21E-27 | 5.36E-26 |
| *ZNF280A* | ENSG00000169548.3 | 5.368600482 | 1.45E-37 | 4.77E-36 |
| *EN2* | ENSG00000164778.4 | 5.487545645 | 8.00E-37 | 2.51E-35 |
| *NEUROG2* | ENSG00000178403.3 | 5.500004669 | 5.55E-24 | 5.01E-23 |
| *NXPH1* | ENSG00000122584.11 | 5.559927978 | 1.66E-21 | 1.16E-20 |
| *GBX2* | ENSG00000168505.6 | 5.602263878 | 4.88E-25 | 4.95E-24 |
| *C6orf15* | ENSG00000204542.2 | 5.658076562 | 7.55E-39 | 2.79E-37 |
| *COL10A1* | ENSG00000123500.8 | 5.686111924 | 6.23E-97 | 2.35E-94 |
| *C5orf46* | ENSG00000178776.4 | 5.701402708 | 2.58E-43 | 1.30E-41 |
| *NOTUM* | ENSG00000185269.10 | 5.725174114 | 3.37E-83 | 8.27E-81 |
| *KLC3* | ENSG00000104892.15 | 5.725951572 | 1.21E-47 | 8.14E-46 |
| *DKK4* | ENSG00000104371.4 | 5.764487124 | 3.08E-48 | 2.16E-46 |
| *ONECUT3* | ENSG00000205922.4 | 5.805788163 | 6.75E-18 | 3.21E-17 |
| *EPYC* | ENSG00000083782.6 | 5.837518656 | 2.62E-34 | 6.64E-33 |
| *RP1* | ENSG00000104237.6 | 5.849748844 | 4.93E-21 | 3.26E-20 |
| *IBSP* | ENSG00000029559.6 | 5.855407036 | 2.42E-51 | 1.88E-49 |
| *DMBX1* | ENSG00000197587.9 | 5.891522874 | 4.16E-46 | 2.58E-44 |
| *SPRR1A* | ENSG00000169474.4 | 5.96495293 | 3.10E-30 | 5.34E-29 |
| *OBP2B* | ENSG00000171102.13 | 6.005572963 | 3.22E-16 | 1.32E-15 |
| *SFTA2* | ENSG00000196260.3 | 6.012624864 | 1.49E-86 | 4.19E-84 |
| *SERPINA4* | ENSG00000100665.10 | 6.046835683 | 4.38E-44 | 2.30E-42 |
| *VGLL1* | ENSG00000102243.11 | 6.105832378 | 1.53E-16 | 6.44E-16 |
| *CST4* | ENSG00000101441.4 | 6.458182044 | 2.16E-47 | 1.43E-45 |
| *KRT75* | ENSG00000170454.5 | 6.494656509 | 6.35E-41 | 2.74E-39 |
| *KLK6* | ENSG00000167755.12 | 6.551945953 | 1.11E-102 | 5.36E-100 |
| *KLK7* | ENSG00000169035.10 | 6.647203018 | 4.53E-50 | 3.34E-48 |
| *SOX14* | ENSG00000168875.2 | 6.737841782 | 7.77E-45 | 4.31E-43 |
| *ELF5* | ENSG00000135374.8 | 6.918904784 | 4.68E-29 | 7.20E-28 |
| *SPERT* | ENSG00000174015.8 | 7.50777876 | 1.17E-69 | 1.87E-67 |
| *KLK8* | ENSG00000129455.14 | 7.663145805 | 2.64E-46 | 1.66E-44 |
| *ZIC5* | ENSG00000139800.8 | 7.864971708 | 2.77E-42 | 1.31E-40 |
| *IGFL1* | ENSG00000188293.5 | 8.336893047 | 5.87E-24 | 5.28E-23 |
| *C8orf74* | ENSG00000171060.9 | 8.412809648 | 5.78E-32 | 1.19E-30 |
| FC: fold change; FDR: false discovery rate | | | | |
|  |  |  |  |  |

**Table S2 Differences in immune cell proportions between three subtypes**

| **Immune cells** | **Sum Sq** | **Mean Sq** | ***P* value** |
| --- | --- | --- | --- |
| naive B cell | 0.100 | 0.069 | 7.62E-01 |
| memory B cell | 1.900 | 1.949 | 1.07E-01 |
| plasma B cell | 12.870 | 12.869 | **3.02E-05** |
| CD8+ T cell | 6.600 | 6.626 | **2.89E-03** |
| naive CD4+ T cell | 0.100 | 0.119 | 6.91E-01 |
| resting memory T cell CD4+ | 0.000 | 0.011 | 9.03E-01 |
| activated memory T cell CD4+ | 0.400 | 0.356 | 4.92E-01 |
| T cell follicular helper | 0.700 | 0.701 | 3.35E-01 |
| regulatory T cell (Tregs) | 6.800 | 6.786 | **2.57E-03** |
| T cell gamma delta | 0.200 | 0.226 | 5.84E-01 |
| resting NK cell | 8.700 | 8.673 | **6.41E-04** |
| activated NK cell | 6.000 | 6.027 | **4.51E-03** |
| Monocyte | 3.400 | 3.436 | 3.24E-01 |
| M0 macrophage | 3.200 | 3.167 | **4.00E-02** |
| M1 macrophage | 10.200 | 10.220 | **2.07E-04** |
| M2 macrophage | 0.700 | 0.744 | 3.20E-01 |
| resting myeloid dendritic cell | 0.100 | 0.098 | 7.18E-01 |
| activated myeloid dendritic cell | 0.200 | 0.247 | 5.67E-01 |
| activated mast cell | 0.000 | 0.019 | 8.75E-01 |
| resting mast cell | 0.900 | 0.880 | 2.80E-01 |
| Eosinophil | 1.000 | 1.029 | 2.42E-01 |
| Neutrophil | 4.400 | 4.409 | **1.53E-02** |

Bold *P* value < 0.05 indicates statistical significance.

**Table S3 Selection of m6A-related DEGs with prognostic independence**

| **Symbol** | **coef** | **se(coef)** | **z** | **Pr(>\|z\|)** | **Hazard Ratio** | **lower.95** | **upper.95** |
| --- | --- | --- | --- | --- | --- | --- | --- |
| *SIGLEC16* | 2.96E-01 | 8.70E-02 | 3.398 | 0.000679 | 1.344 | 1.1333 | 1.5938 |
| *P2RX2* | -8.30E-01 | 2.62E-01 | -3.165 | 0.00155 | 0.436 | 0.2608 | 0.729 |
| *HOXC6* | 0.1083854 | 0.0343793 | 3.153 | 0.00162 | 1.1145 | 1.04186 | 1.1922 |
| *SPOCK3* | 0.433261 | 0.1477952 | 2.931 | 0.00337 | 1.5423 | 1.15441 | 2.0605 |
| *DIAPH3* | -1.18E-01 | 4.21E-02 | -2.812 | 0.004916 | 0.8884 | 0.818 | 0.9648 |
| *RNF32* | 1.19E-01 | 4.64E-02 | 2.566 | 0.010294 | 1.1265 | 1.0285 | 1.2338 |
| *DNAJC28* | -2.88E-01 | 1.14E-01 | -2.54 | 0.011073 | 0.7496 | 0.6001 | 0.9363 |
| *TRPV3* | 2.25E-01 | 8.88E-02 | 2.528 | 0.011482 | 1.2518 | 1.0517 | 1.4898 |
| *CILP2* | -0.1206524 | 0.0488829 | -2.468 | 0.01358 | 0.8863 | 0.80536 | 0.9755 |
| *PIP5KL1* | -0.2507904 | 0.1020126 | -2.458 | 0.01395 | 0.7782 | 0.63716 | 0.9504 |
| *ASIP* | 2.00E-01 | 8.14E-02 | 2.454 | 0.014117 | 1.2211 | 1.0411 | 1.4323 |
| *MESP2* | 0.0832316 | 0.0343111 | 2.426 | 0.01528 | 1.0868 | 1.01611 | 1.1624 |
| *NRXN1* | 1.11E+00 | 4.58E-01 | 2.419 | 0.015562 | 3.0264 | 1.2339 | 7.423 |
| *GRP* | 5.00E-02 | 2.09E-02 | 2.39 | 0.016828 | 1.0512 | 1.009 | 1.0952 |
| *STC2* | 3.41E-02 | 1.46E-02 | 2.34 | 0.019299 | 1.0347 | 1.0055 | 1.0646 |
| *PAK3* | 1.23E+00 | 5.29E-01 | 2.326 | 0.020013 | 3.4201 | 1.2136 | 9.6387 |
| *SSTR3* | 7.38E-01 | 3.18E-01 | 2.326 | 0.020021 | 2.0926 | 1.1232 | 3.8986 |
| *LHB* | -0.1909915 | 0.0837857 | -2.28 | 0.02264 | 0.8261 | 0.70103 | 0.9736 |
| *TEX22* | 2.94E-01 | 1.29E-01 | 2.271 | 0.023126 | 1.3416 | 1.0411 | 1.7287 |
| *JMJD7* | 0.3041335 | 0.1349958 | 2.253 | 0.02426 | 1.3554 | 1.04034 | 1.766 |
| *EEF1A2* | 0.0545604 | 0.0246915 | 2.21 | 0.02713 | 1.0561 | 1.00619 | 1.1084 |
| *AQP7* | 0.1140471 | 0.051643 | 2.208 | 0.02722 | 1.1208 | 1.01291 | 1.2402 |
| *FOXD1* | 4.50E-02 | 2.04E-02 | 2.208 | 0.027227 | 1.046 | 1.0051 | 1.0886 |
| *KLC3* | 0.0799865 | 0.0374089 | 2.138 | 0.0325 | 1.0833 | 1.00669 | 1.1657 |
| *A2ML1* | 8.73E-02 | 4.09E-02 | 2.134 | 0.032872 | 1.0913 | 1.0071 | 1.1824 |
| *NKX6-3* | 4.31E-02 | 2.07E-02 | 2.084 | 0.037179 | 1.044 | 1.0026 | 1.0872 |
| *SLC6A1* | 0.2383268 | 0.1173842 | 2.03 | 0.04232 | 1.2691 | 1.00829 | 1.5974 |
| *SLC6A15* | 0.2304718 | 0.113629 | 2.028 | 0.04253 | 1.2592 | 1.00779 | 1.5733 |
| *CLDN9* | 0.048355 | 0.0240414 | 2.011 | 0.04429 | 1.0495 | 1.00124 | 1.1002 |
| *B3GNT4* | 1.26E-01 | 6.27E-02 | 2.003 | 0.045199 | 1.1337 | 1.0027 | 1.2818 |

DEGs: differential expressed genes.
